# Supplementary material for: Towards general network architecture design criteria for negative gas adsorption transitions in ultraporous frameworks
Source: Nat Commun. 2019 Aug 12;10:3632. doi: 10.1038/s41467-019-11565-3 (PMC6690989; doi:10.1038/s41467-019-11565-3)
Supplement: Supplementary file 1 — Supplementary Information [file 41467_2019_11565_MOESM1_ESM.pdf]

Supplementary Information for

Towards general network architecture design criteria for negative gas adsorption transitions in ultraporous frameworks

Simon Krause[1], Jack D. Evans[1,2], Volodymyr Bon[1], Irena Senkovska[1], Paul Iacomi[3], Felicitas Kolbe[1], Sebastian Ehrling[1], Erik Troschke[1], Jürgen Getzschmann[1], Daniel M. Többens[4], Alexandra Franz[4], Dirk Wallacher[4], Pascal G. Yot[5], Guillaume Maurin[5], Eike Brunner[1], Philip L. Llewellyn[3], François-Xavier Coudert[2], Stefan Kaskel[1*]

Supplementary Methods

Supplementary Table 1. List of chemicals used for the synthesis of ligands and MOFs.

Name	CAS	purity	Manufacturer
1,1′-Ferrocenediyl-bis(diphenylphosphine)	12150-46-8	99%	Alfa Aesar
2,6-Dibromonaphthalene	13720-06-4	99%	ABCR
4,4″-Dibromo-*p*-terphenyl	17788-94-2	n.a.	Sigma Aldrich
4,4′-Diiodobiphenyl	3001-15-8	99%	Alfa Aesar
4-Bromophenylboronic acid	5467-74-3	99%	TCI
9*H*-Carbazole	86-74-8	>95%	Sigma Aldrich
Acetic acid	64-19-7	99.9%	Roth
Bis(dibenzylideneacetone)palladium(II)	32005-36-0	99%	Sigma Aldrich
Copper(I) iodide	7681-65-4	99%	Riedel-de Haen
Copper(II) nitrate trihydrate	10031-43-3	98%	Sigma Aldrich
Hydrochloric acid	7647-01-0	37%	Sigma Aldrich
L-Proline	147-85-3	99%	Sigma Aldrich
N,N′-Ethylenediamine	107-15-3	99%	Alfa Aesar
N,N′-Dimethylethylenediamine	110-70-3	99%	Alfa Aesar
N,N-Dimethylformamide (DMF)	68-12-2	99%	Fischer Scientific
N-Bromosuccinimide	128-08-5	99%	Sigma Aldrich
Potassium hydroxide	1310-58-3	95%	Fischer Scientific
Potassium carbonate	584-08-7	99%	Grüssing
Sodium hydroxide	1310-73-2	98.5%	Fischer Scientific
Sulfuric acid	7664-93-9	99%	Alfa Aesar
Tetrakis(triphenylphosphine)palladium(0)	14221-01-3	99%	Sigma Aldrich
Zinc cyanide	557-21-1	98%	Sigma Aldrich

Supplementary Note 1: Synthesis of organic ligands

Attempts to synthesize L_3-derived ligands by using the synthesis conditions previously used to synthesize L_3 involving lithiation and subsequent carboxylation proved difficult for synthesizing the proposed series of ligands mainly due to low solubility of the bromides and low yields. Thus, we decided to apply a different synthetic pathway (Supplementary Figure 1).

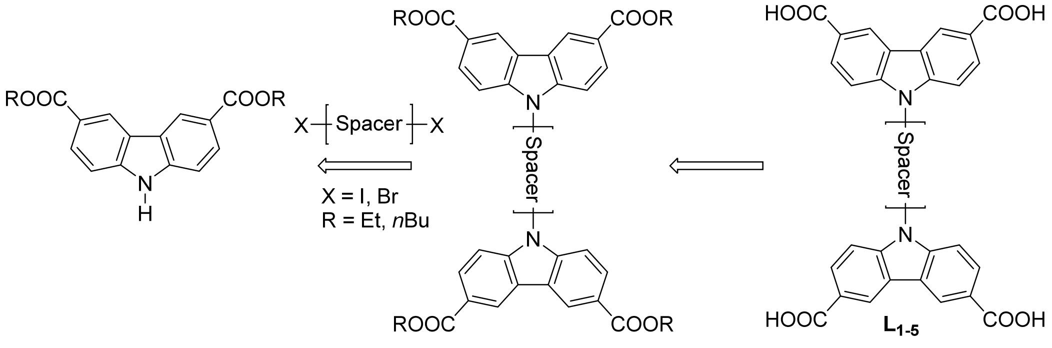

Supplementary Figure 1. Retrosynthesis for the construction of L_1-derrived ligands with different spacer units.

The same approach was previously used in the synthesis of L_1 in DUT-48[2] and PCN-81[3,4] (Supplementary Figure 2).

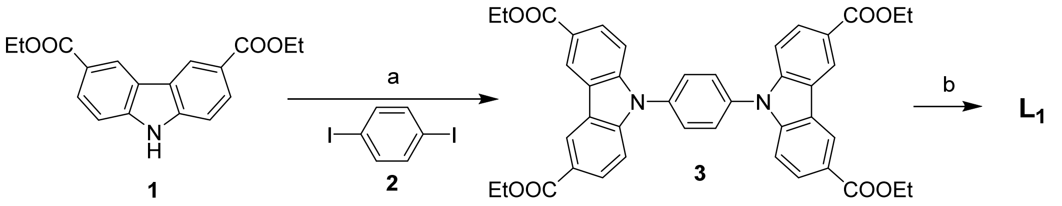

Supplementary Figure 2. Reaction sequence used to synthesize L_1: a) CuI, K_2CO_3, *L*-proline, 90-110°C,1-8 d, b) KOH, THF, H_2O, 90 °C, 24 h.

However, the initially used procedure to synthesise ester **1** showed low yield and difficulties towards upscaling required for the synthesis of 5 different derivatives. Thus, we decided to use a protocol established by Eddaoudi et al.[5] developed to synthesize the carboxylic acid **7** in large quantities and high yields by hydrolysis of the corresponding cyanide which can be synthesized on large scale in two steps based on the commercially available, inexpensive 9*H*-carbazole (**4**).

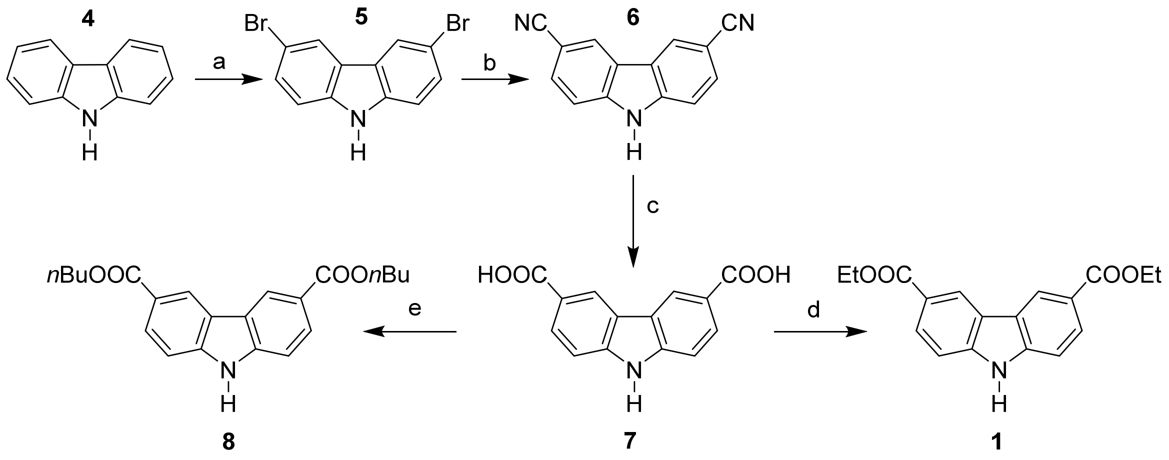

Supplementary Figure 3. Reaction sequence for the synthesis of ester **1** and **8**. a) *N*-Bromosuccinimide, THF, 30 °C, 24 h, b) $ZnCN_2$, ZnOAc, Pd(dba), 1,1′-Bis(diphenylphosphino)ferrocene, DMF, 100 °C, 72 h, c) CuI (cat.), NaOH, H_2O, 130 °C, 24 h, d) ethanol, H_2SO_4 (cat.), 85 °C, 18 h, e) *n*-butanol, H_2SO_4 (cat.), 130 °C, 48 h.

By esterification with ethanol ester **1** could be obtained in overall higher yields than the previously reported procedure and the reaction sequence allows for easier upscaling. However, attempts to synthesize L_2 and L_3 by using the ethyl ester **1** as starting material lead to the formation of the mono-coupled product which precipitated from the reaction mixture and demonstrated low solubility in DMSO and DMF. To overcome these limits of solubility we decided to substitute ethyl by *n*-butyl groups which should in general support solubility. By esterification with *n*-butanol, the *n*-butyl ester **8** could be obtained on the scale of 45 g product and overall yield of 71% over four steps. When reacted with iodide **11** via Cu-catalysed Ullmann-coupling in DMF, potassium carbonate as base and *N,N′*-dimethylethylenediamine to stabilize the catalyst at 120 °C the *n*-butyl ester **12** could be obtained with 43% yield in contrast to the previous unsuccessful synthesis using the ethyl ester **1**. The Cu-based Ullman coupling reaction was found to be the most efficient and cheapest method in contrast to Pd-based catalysts. The following reaction sequences were used to synthesize L_2-L_5 (Supplementary Figure 4).

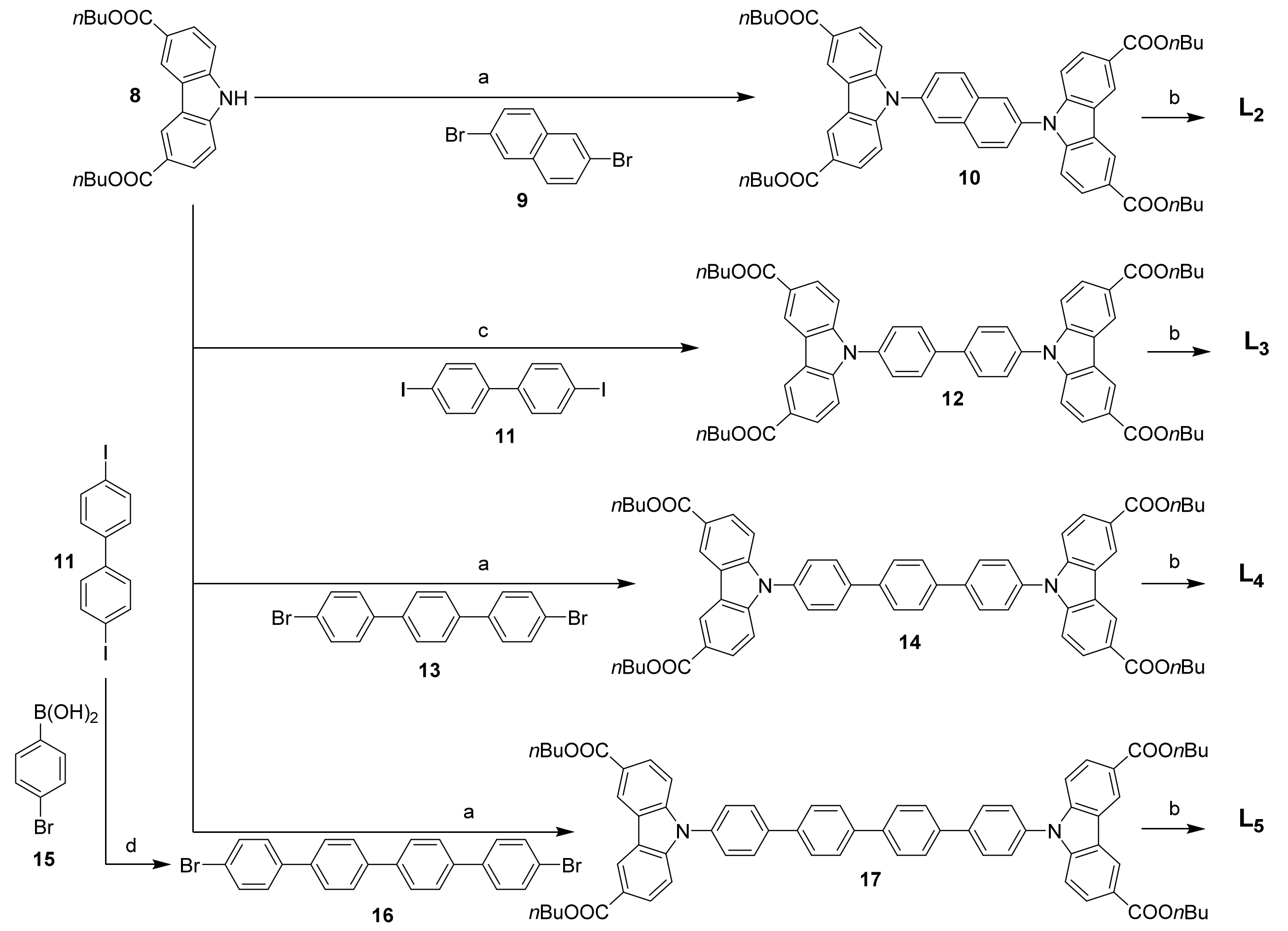

Supplementary Figure 4. Scheme for the synthesis of ligands L_1 – L_5. a) CuI, K_2CO_3, DMF, *N,N'*-dimethyl ethylenediamine, 90-110°C,1-8 d, b) KOH, THF, H_2O, 90 °C, 24 h, c) CuI, K_2CO_3, DMSO, *L*-proline, 90-110°C,1-8 d, d) tetrakis(triphenylphosphine)palladium(0), K_2CO_3, toluene, H_2O, 100 °C, 24 h.

Based on whether the coupling reaction was performed with an iodide or bromide, reaction conditions were adjusted. Detailed description on all reactions can be found in the following:

3,6-Dibromo-9*H*-carbazole

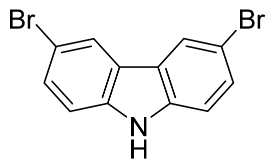

A 1 l flask was charged with 60 g (0.36 mol) 9*H*-carbazole which was previously recrystallized from a mixture of 900 ml toluene and 100 ml ethanol and dissolved in 800 ml anhydrous THF. To the solution 140 g (0.79 mol) *N*-bromosuccinimide was added over 30 min at room temperature and the solution was stirred for 28 h at 30 °C. The THF was removed in vacuum, the remaining solid dissolved in diethyl ether, and the solution extracted with water. The organic phases were collected, dried over $MgSO_4$ and the solvent removed in vacuum. The yellow powder was recrystallized from a mixture of 800 ml chloroform and 50 ml diethyl ether and dried in vacuum to yield 103 g (89%) of white product.

^{1}H NMR (500 MHz, CHLOROFORM-*d*) δ (ppm): 7.33 (d, *J*=8.51 Hz, 2 H) 7.53 (dd, *J*=8.51, 1.89 Hz, 2 H) 8.15 (d, *J*=1.89 Hz, 2 H) 8.33 (br. s., 1 H).

^{13}C NMR (151 MHz, CHLOROFORM-*d*) δ ppm 111.34 (s, 1 C) 113.69 (s, 1 CH) 123.45 (s, 1 CH) 123.99 (s, 1 C) 128.87 (s, 1 CH) 138.91 (s, 1 CH).

ASAP-MS (m/z): Calculated for $C_{12}H_7Br_2N$: 324; found: 324 $[M-H]^+$.

9*H*-Carbazole-3,6-dicarbonitrile

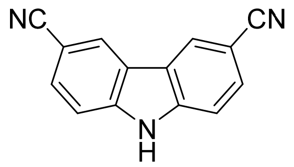

Due to the high toxicity of cyanide the reaction was performed in a sealed fume hood. Deactivation of glassware and tools used for the reaction were performed in a 5% H_2O_2 solution containing ammonia with a pH higher than 11. A 500 ml Schlenk flask was charged with 80 g (246 mmol) of bromide **5** and 656 mg (0.59 mmol) 1,1′-bis(diphenylphosphino)ferrocene. The flask was evacuated, flushed with Ar, and 250 ml DMF and 2.4 ml water were added. In a glovebox a flask with 640 mg (9.9 mmol) Zn powder, 34.5 g (294 mmol) anhydrous zink cyanide, 1.8 g (9.8 mmol) zinc acetate, and 460 mg (0.5 mmol) bis(dibenzylideneacetone)palladium(0) was prepared and the reactants added to the Schlenk flask. The reaction mixture was stirred at 100 °C for 72 h. Afterwards the brown suspension was cooled to room temperature and poured into an aqueous solution of 400 ml saturated ammonium chloride solution and 400 ml concentrated ammonia solution. The off-white precipitate was filtered off and washed with the same amount of previously described aqueous solution followed by thorough washing with water. The waste solutions were collected and deactivated as described above. The off-white powder was dried, washed twice with 20 ml methanol, twice with 30 ml toluene, and dried in vacuum. The solid was recrystallized from DMF to yield 48.2 g (90%) of the white product.

^{1}H NMR (500 MHz, DMSO-d_6) δ (ppm): 7.72 (d, *J*=8.51 Hz, 2 H) 7.85 (dd, *J*=8.20, 1.58 Hz, 2 H) 8.79 (s, 2 H) 12.38 (s, 1 H).

^{13}C NMR (151 MHz, DMSO-d_6) δ ppm 101.59 (s, 1 CH) 112.69 (s, 1 CH) 119.95 (s, 1 C) 121.69 (s, 1 C) 126.26 (s, 1 CH) 129.77 (s, 1 CH) 142.16 (s, 1 C).

MS-ASAP (m/z): Calculated for $C_{14}H_7N_3$: 218; found: 218 $[M+H]^+$.

Elemental analysis: Calculated: C: 77.41%; H: 3.25%; N: 19.34%; found: C: 78.19%; H: 3.056%; N: 19.47%.

9*H*-Carbazole-3,6-dicarboxylic acid

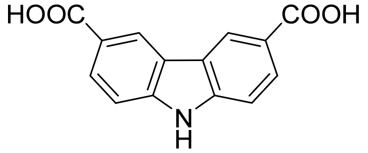

In a 2 l flask 30 g (0.13 mol) cyanide **6**, 90 g (2.25 mol) sodium hydroxide, and 300 mg (1.58 mmol) copper (I) iodide were dissolved in 1 L water and stirred under reflux for 24 h. the solution was cooled down to room temperature and filtered over Celite®. The solution was neutralized with 6 M hydrochloric acid, the white precipitate was filtered off, washed thoroughly with water, and dried at 80 °C to yield 33 g (93%) off-white powder.

^{1}H NMR (500 MHz, DMSO-d_6) δ ppm 7.57 (d, *J*=8.51 Hz, 2 H) 8.03 (dd, *J*=8.51, 1.89 Hz, 2 H) 8.79 - 8.85 (m, 2 H) 12.02 (s, 1 H)

^{13}C NMR (126 MHz, DMSO-d_6) δ ppm 11.121 (s, 1 CH), 122.03 (s, 1 C) 122.33 (s, 1 C) 122.95 (s, 1 CH) 127.80 (s, 1 CH) 143.26 (s, 1 C) 167.07 (s, 1 C) 168.82 (s, 1 C).

MS-ASAP (m/z): Calculated for $C_{14}H_9NO_4$: 256; found: 256 $[M+H]^+$.

Elemental analysis: Calculated ($C_{14}H_9NO_4$ 0.55·H_2O): C: 63.42%; H: 3.84%; N: 5.28%; found: C: 63.44%; H: 3.681%; N: 5.08%.

DRIFT, KBr, 298 K (cm^{-1}): 3471 (w) , 2823 (w) , 2644 (w) , 1913 (w) , 1789 (m) , 1681 (s) , 1633 (m) , 1606 (s) , 1585 (m) , 1491 (w) , 1470 (m) , 1451 (m) , 1419 (s) , 1354 (m) , 1288 (s) , 1254 (s) , 1217 (m) , 1137 (w) , 1024 (m) , 931 (m) , 898 (m) , 851 (w) , 828 (m) , 802 (w) , 769 (s) , 724 (m) , 682 (w).

Diethyl 9*H*-carbazole-3,6-dicarboxylate

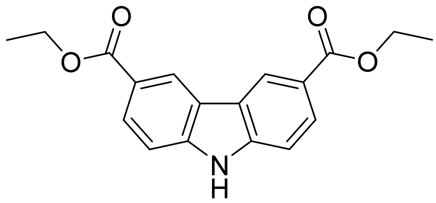

In a 250 ml flask 3 g (11.7 mmol) H_2CDC **1** was suspended in 150 ml ethanol and 1 ml of sulfuric acid was added. The mixture was refluxed at 85 °C for 18 h to form a clear yellow solution. The ethanol was removed in vacuum, the resulting solid dissolved in chloroform, and extracted with diluted aqueous potassium carbonate solution. The organic phases were combined, dried over $MgSO_4$ and the solvent removed in vacuum to obtain 3.3 g (91%) white powder.

^{1}H NMR (500 MHz, CHLOROFORM-*d*) δ (ppm): 1.48 (t, *J*=7.09 Hz, 3 H) 4.46 (q, *J*=7.25 Hz, 2 H) 7.48 (d, *J*=8.51 Hz, 2 H) 8.20 (dd, *J*=8.51, 1.58 Hz, 2 H) 8.58 (br. s., 1 H) 8.88 (d, *J*=1.26 Hz, 2 H).

^{13}C-NMR (150 MHz, DMSO-d_6): δ (in ppm): 14.38 (1 CH_3), 60.39 (1 CH_2), 111.31 (1 CH), 121.24 (1 C), 122.29 (1 C), 122.77 (1 CH), 127.46 (1 CH), 143.25 (1 C), 166.33 (1 C).

MALDI-TOF-MS (m/z): Calculated for $C_{18}H_{17}NO_4$: 266; found 266 $[M-C_2H_5OH]^+$.

Elemental analysis: Calculated: C: 69.44%; H: 5.5%; N: 4.5%; found: C: 68.73%; H: 5.299%; N: 4.5%.

Dibutyl 9*H*-carbazole-3,6-dicarboxylate

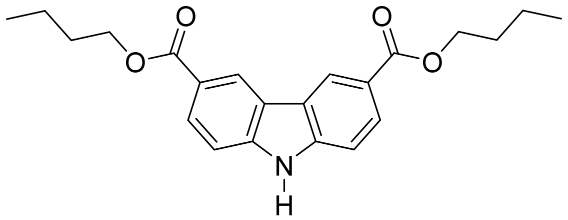

In a 1 l flask 33 g (0.13 mmol) H_2CDC **1** was suspended in 750 ml 1-butanol and 3 ml of sulfuric acid was added. The mixture was refluxed at 130 °C for 48 h to form a clear yellow solution. The 1-butanol was removed in vacuum, the resulting solid dissolved in chloroform, and extracted with diluted aqueous potassium carbonate solution. The organic phases were combined, dried over $MgSO_4$ and the solvent removed in vacuum. The obtained solid was recrystallized from ethyl acetate to obtain 45 g (95%) white powder.

^{1}H NMR (600 MHz, CHLOROFORM-*d*) δ (ppm): 1.04 (t, *J*=7.34 Hz, 6 H) 1.51 - 1.60 (m, 4 H) 1.81 - 1.88 (m, 4 H) 4.41 (t, *J*=6.59 Hz, 4 H) 7.48 (d, *J*=8.28 Hz, 2 H) 8.19 (dd, *J*=8.47, 1.69 Hz, 2 H) 8.57 (br. s., 1 H) 8.87 (d, *J*=1.51 Hz, 2 H).

^{13}C NMR (151 MHz, CHLOROFORM-*d*) δ (ppm): 13.83 (s, 1 CH_3) 19.36 (s, 1 CH_2) 30.93 (s, 1 CH_2) 64.82 (s, 1 CH_2) 110.47 (s, 1 CH) 122.75 (s, 1 C) 123.04 (s, 1 CH) 123.14 (s, 1 C) 128.13 (s, 1 CH) 142.66 (s, 1 C) 167.20 (s, 1 C).

MALDI-TOF-MS (m/z): Calculated for $C_{36}H_{20}Br_4N_2$: 294; found 294 $[M-C_4H_9OH]^+$.

Elemental analysis: Calculated: C: 71.91%; H: 6.86%; N: 3.81%; found: C: 72.01%; H: 6.443%; N: 3.68%.

Synthesis of L_3 and analogues

General procedure of Ullman coupling

The general synthesis of ligands derived from H_2CDC is based on Ullmann coupling of the esters **8** or **1** with different iodides and bromines. The following procedures were used:

Ullmann Coupling with iodides

This procedure is based on syntheses previously used for carbazole based ligands[6]. A Schlenk flask was charged with indicated amounts of ester **8**, the corresponding iodide, potassium carbonate, copper (I) iodide, and *L*-proline under inert atmosphere. To the mixture indicated amounts of degassed DMSO or DMF were given and Ar was bubbled through the suspension for 30 min. The reaction mixture was stirred at 90 – 120 °C for 24 h to 10 d and the reaction was cooled down to room temperature. The suspension was quenched with diluted (< 0.02 M) hydrochloric acid and extracted with chloroform. The organic phases were collected, dried over $MgSO_4$, and the solvent removed in vacuum. The crude product was purified by flash column chromatography using indicated mixtures of chloroform, DCM, *iso*-hexane and ethyl acetate. Corresponding amounts of the chemicals added and used for the synthesis and purification are provided for each coupling product.

Ullmann Coupling with bromides

This procedure is based on syntheses previously used for carbazole based ligands[4]. A Schlenk flask was charged with indicated amounts of ester **8**, the corresponding bromide, potassium carbonate, copper (I) iodide, and *N,N'*-dimethylethylenediamine under inert atmosphere. To the mixture indicated amounts of degassed anhydrous 1,4-dioxane were given and Ar was bubbled through the suspension for 30 min. The reaction mixture was stirred at 80 – 110 °C for 24 h to 12 d and the reaction was cooled down to room temperature. The solvent was removed in vacuum and the obtained powder was dissolved in chloroform and extracted with diluted (< 0.02 M) hydrochloric acid. The organic phases were collected, dried over $MgSO_4$, and the solvent removed in vacuum. The crude product was purified by flash column chromatography using indicated mixtures of chloroform, DCM, *iso*-hexane and ethyl acetate. Corresponding amounts of the chemicals added and used for the synthesis and purification, reaction times and temperatures are provided for each coupling product.

General procedure for ester hydrolysis

To hydrolyse the ester groups the corresponding coupling products were dissolved in indicated volumes of THF, methanol, and water at 85 °C. To the solution potassium hydroxide was added and the mixture was stirred at 85 °C for 12 h– 5 d. In case a precipitate formed from the previous clear solution (most

likely the potassium salt of the hydrolysed ester which is insoluble in THF) small amounts of water were added until a clear solution formed again. After the indicated reaction time THF and methanol were removed in vacuum, the resulting solution was filtered, and neutralized with 2 M hydrochloric acid. The precipitate was filtered off and dried in vacuum at room temperature. Corresponding amounts of the chemicals added and reaction times are provided for each hydrolysis product.

Variation of ligand length

Tetraethyl 9,9'-(1,4-phenylene)bis(9H-carbazole-3,6-dicarboxylate)

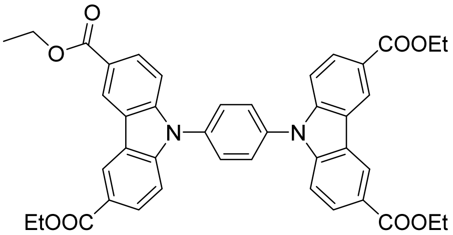

Synthesis conditions: 4.5 g (14.4 mmol) ethylester **1**, 2.16 mg (0.74 mmol) 4,4′-diiodophenyl, 3.63 g (26.3 mmol) potassium carbonate, 250 mg (1.31 mmol) copper(I) iodide, 104 mg (2.62 mmol) *L*-proline, 80 ml DMSO, 85 °C for 24 h and 95 °C for 48 h; Recrystallized from ethyl acetate.

Yield: 3 g (65%) white powder.

^{1}H-NMR (500 MHz, DMSO-d_6): δ (ppm): 1.38 (tr, J = 7.0 Hz, 6 H), 4.33 (q, J = 7.0 Hz, 4 H), 7.65 (dd, J = 8.5 Hz, J = 0.6 Hz, 2 H), 8.11 (dd, J = 8.5 Hz, J = 1.7, 2 H), 8.89 (dd, J = 1.7 Hz, J = 0.6 Hz, 2 H), 12 (s, 1 H).

^{13}C-NMR (150 MHz, DMSO-d_6): δ (ppm): 14.34 (1 CH_3), 60.39 (1 CH_2), 111.35 (1 CH), 121.25 (1 C), 122.29 (1 C), 122.71 (1 CH), 127.486 (1 CH), 143.25 (1 C), 166.39 (1 C).

9,9'-(1,4-phenylene)bis(9H-carbazole-3,6-dicarboxylic acid)

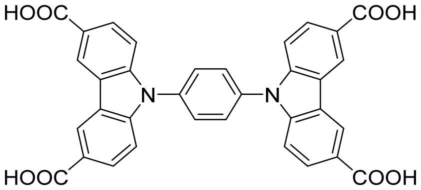

Synthesis conditions: 3 g (4.31 mmol) ester **3**, 1.44 g (20.7 mmol) potassium hydroxide, 115 ml THF, 3 ml methanol, 50 ml H_2O, 75 °C for 2 d.

Yield: 2.35 g (93%) white powder.

^{1}H NMR (500 MHz, DMSO-d_6) δ (ppm): 7.68 (d, *J*=8.51 Hz, 1 H) 8.03 (s, 1 H) 8.15 (dd, *J*=8.83, 1.58 Hz, 1 H) 9.01 (d, *J*=1.58 Hz, 1 H) 12.69 (s, 1 H)

^{13}C-NMR (150 MHz, DMSO-d_6) δ (ppm):110.04 (1 CH), 123.1 (1 C), 123.51 (1 CH), 124.02 (1 C), 128.79 (1 CH), 129.29 (1 CH), 136.03 (1 C), 143.85 (1 C), 168 (1 C).

Elemental analysis: Calculated ($C_{34}H_{20}N_2O_8$ 1.76·H_2O): C: 66.27%; H: 3.85%; N: 4.55%; found: C: 66.22%; H: 3.85%; N: 4.61%.

DRIFT, KBr, 298 K (cm^{-1}): 3070 (w), 2647 (w), 1694 (w), 1627 (s), 1602 (m), 1518 (s), 1475 (m), 1416 (m), 1339 (w), 1292 (s), 1266 (s), 1179 (m), 1137 (w), 1027 (s), 950 (w), 904 (m), 818 (m), 805 (w), 768 (s), 722 (w), 703 (w), 666 (w).

Tetrabutyl 9,9'-(naphthalene-2,6-diyl)bis(9H-carbazole-3,6-dicarboxylate)

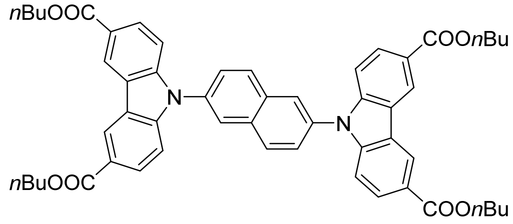

Synthesis conditions: 3.76 g (10.2 mmol) *n*-butylester **8**, 0.98 g (3.41 mmol) 2,6-dibromonaphtalene, 1.88 g (13.6 mmol) potassium carbonate, 428 mg (2.12 mmol) copper(I) iodide, 0.1 ml (0.91 mmol) *N,N'*-dimethylethylenediamine, 30 ml anhydr. 1,4 dioxane, 110 °C for 3 d; Flash column chromatography chloroform : *iso*-hexane : ethyl acetate = 1 : 0.03 : 0.06 (R_f 0.53); Yield: 2.81 g (96%) white powder.

^{1}H NMR (500 MHz, CHLOROFORM-*d*) δ (ppm): 1.05 (t, *J*=7.41 Hz, 6 H) 1.52 - 1.65 (m, 4 H) 1.83 - 1.92 (m, 4 H) 4.44 (t, *J*=6.78 Hz, 4 H) 7.51 (d, *J*=8.51 Hz, 2 H) 7.81 (dd, *J*=8.67, 1.73 Hz, 1 H) 8.21 (d, *J*=1.89 Hz, 1 H) 8.22 - 8.25 (m, 3 H) 8.99 (s, 2 H).

^{13}C NMR (126 MHz, CHLOROFORM-*d*) δ (ppm): 14.10 (s, 1 CH_3) 19.63 (s, 1 CH_2) 31.18 (s, 1 CH_2) 65.17 (s, 1 CH_2) 109.92 (s, 1 CH) 123.37 (s, 1 C) 123.58 (s, 1 CH) 123.73 (s, 1 C) 126.03 (s, 1 CH) 126.42 (s, 1 C) 128.61 (s, 1 CH) 130.66 (s, 1 CH) 133.54 (s, 1 C) 135.32 (s, 1 C) 144.44 (s, 1 C) 167.28 (s, 1 C) 171.41 (s, 1 C).

Elemental analysis: Calculated: C: 75.5%; H: 6.34%; N: 3.26%; found: C: 75.93%; H: 6.342%; N: 2.91%.

9,9'-(naphthalene-2,6-diyl)bis(9H-carbazole-3,6-dicarboxylic acid)

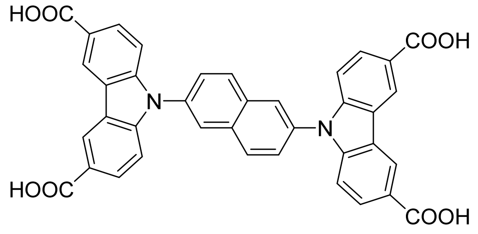

Synthesis conditions: 1.7 g (1.98 mmol) ester **10**, 1 g (17.6 mmol) potassium hydroxide, 33 ml THF, 5 ml methanol, 16 ml H_2O, 75 °C for 3 d.; Yield: 1.18 g (94%) white powder.

^{1}H NMR (500 MHz, DMSO-d_6) δ (ppm): 7.59 (d, *J*=8.51 Hz, 2 H) 7.91 - 7.99 (m, 1 H) 8.14 (dd, *J*=8.83, 1.26 Hz, 2 H) 8.44 (d, *J*=8.83 Hz, 1 H) 8.53 (s, 1 H) 9.03 (s, 2 H) 12.84 (br. s., 2 H).

^{13}C NMR (126 MHz, DMSO-d_6) δ (ppm): 110.09 (s, 1 CH) 122.66 (s, 1 C) 123.13 (s, 1 CH) 123.61 (s, 1 C) 125.78 (s, 1 CH) 125.95 (s, 1 C) 128.43 (s, 1 CH) 130.69 (s, 1 CH) 133.04 (s, 1 C) 134.26 (s, 1 C) 143.63 (s, 1 C) 167.63 (s, 1 C).

MALDI-TOF-MS (m/z): Calculated for $C_{38}H_{22}N_2O_8$: 634; found 634 $[M]^+$.

Elemental analysis: Calculated ($C_{38}H_{22}N_2O_8$ 1.15·H_2O): C: 70.29%; H3.84%; N: 4.41%; found: C: 70.29%; H: 3.84%; N: 3.56%.

DRIFT, KBr, 298 K (cm^{-1}): 3065(w), 2642 (w), 1694 (s), 1629 (m), 1601 (s), 1508 (w), 1477 (s), 1393 (s), 1342 (m), 1236 (s), 1159 (m), 1137 (m), 1027 (w), 923 (w), 904 (m), 828 (m), 768 (s), 737 (w), 724 (w), 687 (w).

Tetrabutyl 9,9'-([1,1'-biphenyl]-4,4'-diyl)bis(9H-carbazole-3,6-dicarboxylate)

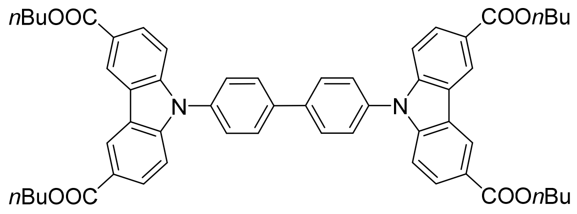

Synthesis conditions: 815 mg (2.22 mmol) *n*-butylester **8**, 300 mg (0.74 mmol) 4,4′-diiodobiphenyl, 224 mg (1.62 mmol) potassium carbonate, 90 mg (0.66 mmol) copper(I) iodide, 104 mg (0.9 mmol) *L*-proline, 15 ml anhydr. DMF, 120 °C for 48 h; Flash column chromatography DCM : chloroform : ethyl acetate – 0.1:1:0.010 (R_f 0.6); Yield: 280 mg (43%) white powder.

^{1}H NMR (300 MHz, CHLOROFORM-*d*) δ (ppm): 1.05 (t, *J*=7.37 Hz, 4 H) 1.57 (dq, *J*=14.94, 7.36 Hz, 3 H) 1.76 - 1.93 (m, 3 H) 4.43 (t, *J*=6.61 Hz, 3 H) 7.50 (d, *J*=8.69 Hz, 2 H) 7.73 (d, *J*=8.50 Hz, 2 H) 7.98 (d, *J*=8.50 Hz, 2 H) 8.21 (dd, *J*=8.69, 1.70 Hz, 2 H) 8.96 (d, *J*=1.32 Hz, 2 H).

^{13}C NMR (126 MHz, CHLOROFORM-*d*) δ (ppm): 13.81 (s, 1 CH_3) 19.34 (s, 1 CH_2) 30.88 (s, 1 C H_2) 64.85 (s, 1 C H_2) 109.72 (s, 1 CH) 122.98 (s, 1 CH) 123.19 (s, 1 C) 123.25 (s, 1 C) 127.52 (s, 1 CH) 128.20 (s, 1 CH) 128.85 (s, 1 CH) 136.14 (s, 1 C) 140.01 (s, 1 C) 144.02 (s, 1 C) 167.00 (s, 1 C).

Elemental analysis: Calculated: C: 76%; H: 6.38%; N: 3.17%; found: C: 75.69%; H: 6.172%; N: 2.98%.

9,9'-([1,1'-biphenyl]-4,4'-diyl)bis(9H-carbazole-3,6-dicarboxylic acid)

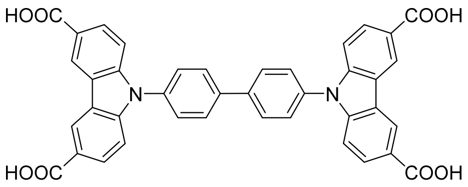

Synthesis conditions: 150 mg (0.17 mmol) ester **12**, 700 mg (12.3 mmol) potassium hydroxide, 18 ml THF, 1 ml methanol, 0.5 ml H_2O + 3 ml H_2O after 1 d, 85 °C for 72 h; Yield: 60 mg (54%) white powder.

^{1}H NMR (300 MHz, DMSO-d_6) δ (ppm): 7.55 (d, *J*=8.69 Hz, 1 H) 7.86 (d, *J*=8.50 Hz, 1 H) 8.09 - 8.20 (m, 2 H) 9.01 (d, *J*=1.51 Hz, 1 H) 12.85 (br. s., 1 H).

^{13}C NMR (75 MHz, DMSO-d_6) δ (ppm): 110.02 (s, 1 CH) 122.61 (s, 1 C) 123.06 (s, 1 CH) 123.51 (s, 1 C) 127.56 (s, 1 CH) 128.34 (s, 1 CH) 128.80 (s, 1 CH) 135.50 (s, 1 C) 139.17 (s, 1 C) 143.42 (s, 1 C) 167.61 (s, 1 C).

Elemental analysis: Calculated ($C_{40}H_{24}N_2O_8$ 3·H_2O): C: 67.22%; H: 4.23%; N: 3.92%; found: C: 66.78%; H: 3.73%; N: 3.96%.

DRIFT, KBr, 298 K (cm^{-1}): 3037 (w), 2619 (w), 1894 (m), 1657 (s), 1631 (s), 1602 (s), 1503 (s), 1403 (m), 1347 (m), 1298 (m), 1258 (s), 1186 (m), 1169 (m), 1135 (s), 1105 (w), 1062 (s), 1027 (m), 1007 (w), 915 (m), 899 (w), 855 (w), 813 (w), 757 (m), 715 (w), 669 (w).

Tetrabutyl 9,9'-([1,1':4',1''-terphenyl]-4,4''-diyl)bis(9H-carbazole-3,6-dicarboxylate)

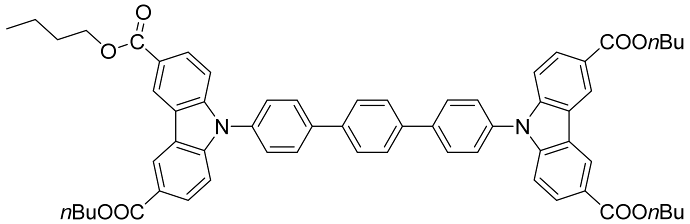

Synthesis conditions: 5.71 g (15.5 mmol) *n*-butylester **8**, 2 g (5.18 mmol) 4,4′′-dibromo-*p*-terphenyl, 2.86 g (20.7 mmol) potassium carbonate, 326 mg (1.71 mmol) copper(I) iodide, 0.1 ml (0.91 mmol) *N,N′*-dimethylethylenediamine, 80 ml anhydr. 1,4 dioxane, 110 °C for 8 d; Flash column chromatography chloroform : *iso*-hexane : ethyl acetate – 1 : 0.5 : 0.02 (R_f 0.55); Yield: 3.65 g (95%) white powder.

^{1}H NMR (500 MHz, CHLOROFORM-*d*) δ (ppm): 1.05 (t, *J*=7.41 Hz, 3 H) 1.57 (s, 2 H) 1.72 - 1.90 (m, 2 H) 4.43 (t, *J*=6.62 Hz, 2 H) 7.50 (d, *J*=8.83 Hz, 1 H) 7.69 (d, *J*=8.51 Hz, 1 H) 7.88 (s, 1 H) 7.96 (d, *J*=8.20 Hz, 1 H) 8.21 (dd, *J*=8.51, 1.58 Hz, 1 H) 8.96 (d, *J*=1.58 Hz, 1 H).

^{13}C NMR (126 MHz, CHLOROFORM-*d*) δ (ppm): 13.84 (s, 1 CH_3) 19.38 (s, 1 CH_2) 30.93 (s, 1 CH_2) 64.88 (s, 1 CH_2) 109.80 (s, 1 CH) 123.03 (s, 1 C) 123.20 (s, 1 C) 123.24 (s, 1 CH) 127.46 (s, 1 CH) 127.80 (s, 1 CH) 128.22 (s, 1 CH) 128.73 (s, 1 CH) 135.82 (s, 1 C) 139.43 (s, 1 C) 140.65 (s, 1 C) 144.16 (s, 1 C) 167.14 (s, 1 C).

MALDI-TOF-MS (m/z): Calculated for $C_{62}H_{60}N_2O_8$: 887; found: 887 $[M-C_4H_9OH]^+$.

Elemental analysis: Calculated: C: 77.48%; H: 6.29%; N: 2.91%; found: C: 77.57%; H: 5.46%; N: 2.93%.

9,9'-([1,1':4',1''-Terphenyl]-4,4''-diyl)bis(9H-carbazole-3,6-dicarboxylic acid)

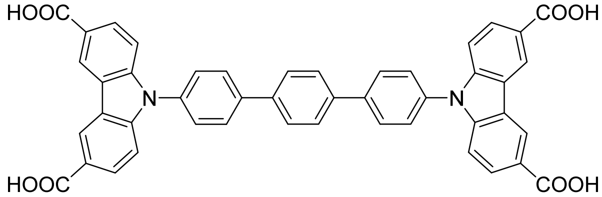

Synthesis conditions: 3.6 g (3.75 mmol) ester **14**, 8 g (144 mmol) potassium hydroxide, 350 ml THF, 30 ml methanol, 2 ml H_2O, 90 °C for 24 h; Yield: 2.65 g (96%) white powder.

^{1}H NMR (500 MHz, DMSO-d_6) δ (ppm): 7.53 (d, *J*=8.51 Hz, 1 H) 7.79 (d, *J*=7.88 Hz, 1 H) 7.96 (s, 1 H) 8.08 (d, *J*=8.20 Hz, 1 H) 8.12 (d, *J*=8.51 Hz, 1 H) 8.99 (s, 1 H) 12.79 (br. s., 1 H).

^{13}C NMR (126 MHz, DMSO-d_6) δ (ppm): 109.89 (s, 1 CH) 122.47 (s, 1 C) 122.93 (s, 1 C) 123.36 (s, 1 CH) 127.32 (s, 1 CH) 127.43 (s, 1 CH) 128.23 (s, 1 C) 128.35 (s, 1 CH) 135.09 (s, 1 C) 138.38 (s, 1 C) 139.40 (s, 1 C) 143.32 (s, 1 C) 167.53 (s, 1 C).

Elemental analysis: Calculated ($C_{46}H_{28}N_2O_8 \cdot 3.85H_2O$): C: 68.54%; H: 4.46%; N: 3.48%; found: C: 68.15%; H: 4.027%; N: 3.40%.

DRIFT, KBr, 298 K (cm^{-1}): 3069 (w), 2628 (w), 1907 (w), 1695 (s), 1630 (m), 1600 (s), 1533 (w), 1494 (s), 1365 (m), 1291 (s), 1235 (s), 1173 (m), 1137 (m), 1027 (w), 1005 (m), 949 (w), 905 (w), 815 (s), 770 (s), 726 (m), 680 (w).

4,4'''-Dibromo-1,1':4',1'':4'',1'''-quaterphenyl

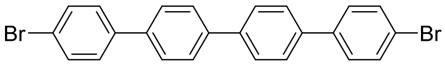

A flame-dried 500 ml Schlenk flask was charged with 1.7 g (3.36 mmol) iodide **11**, 1.45 g (7.22 mmol) 4-bromophenylboronic acid **15**, 2.7 g (19.5 mmol) potassium carbonate, 43 mg (0.037 mmol) tetrakis(triphenylphosphine)palladium(0), and a degassed mixture of 105 ml toluene, 15 ml water and 60 ml methanol. The brown suspension was refluxed under inert atmosphere at 110 °C for 3 d. The solvents were removed in vacuum and the remaining solid dissolved in chloroform, extracted with water, and the combined organic phases dried over $MgSO_4$. The obtained yellow solid was purified by flash column chromatography using an iso-hexane : chloroform (2 : 1) mixture (R_f 0.54) to obtain 850 mg (45 %) of white powder.

No NMR spectra could be collected due to low solubility in common organic solvents.

MALDI-TOF-MS (m/z): Calculated for $C_{24}H_{16}Br_2$: 463; found: 463 $[M]^+$.

Elemental analysis: Calculated: C: 62.1%; H: 3.47%; found: C: 62.22%; H: 3.54%.

DRIFT, KBr, 298 K (cm^{-1}): 2585 (w), 2297 (w), 1912 (m), 1898 (m), 1778 (w), 1681 (w), 1646 (w), 1596 (m), 1476 (s), 1436 (m), 1388 (s), 1347 (m), 1281 (m), 1181 (w), 1141 (m), 1121 (m), 1103 (m), 1075 (s), 1043 (w), 1009 (m), 1000 (s), 964 (w), 860 (m), 843 (m), 822 (s), 807 (s), 764 (m), 750 (m), 742 (m), 695 (m), 660 (w), 642 (m), 624 (w).

Tetrabutyl 9,9'-([1,1':4',1'':4'',1'''-quaterphenyl]-4,4'''-diyl)bis(9H-carbazole-3,6-dicarboxylate)

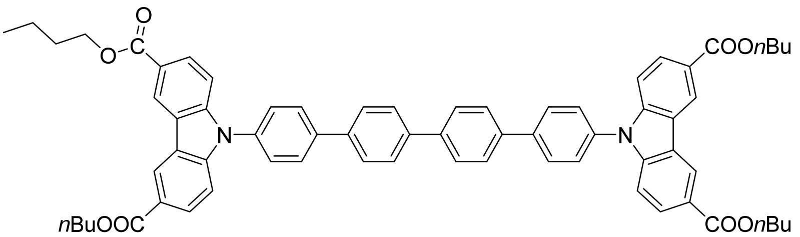

Synthesis conditions: 7.12 g (19.4 mmol) *n*-butylester **8**, 3 g (6.46 mmol) 4,4'''-dibromo-1,1':4',1'':4'',1'''-quaterphenyl **16**, 3.57 g (25.8 mmol) potassium carbonate, 613 mg (3.23 mmol) copper(I) iodide, 0.1 ml (0.91 mmol) *N,N'*-dimethylethylenediamine, 80 ml anhydr. 1,4 dioxane, 110 °C for 8 d; Flash column chromatography chloroform : *iso*-hexane : ethyl acetate – 1 : 0.3 : 0.01 (R_f 0.29); Yield: 1.96 g (29%) white powder.

^{1}H NMR (500 MHz, CHLOROFORM-*d*) δ (ppm): 1.05 (t, *J*=7.41 Hz, 3 H) 1.51 - 1.70 (m, 1 H) 1.74 - 1.91 (m, 1 H) 4.43 (t, *J*=6.62 Hz, 1 H) 7.49 (d, *J*=8.83 Hz, 1 H) 7.67 (d, *J*=8.51 Hz, 1 H) 7.84 (d, *J*=2.21 Hz, 2 H) 7.94 (d, *J*=8.51 Hz, 1 H) 8.20 (dd, *J*=8.51, 1.58 Hz, 1 H) 8.96 (d, *J*=1.58 Hz, 1 H).

^{13}C NMR (126 MHz, CHLOROFORM-*d*) δ (ppm): 14.10 (s, 1 CH_3) 19.63 (s, 1 CH_2) 31.17 (s, 1 C) CH_2) 65.12 (s, 1 C CH_2)) 110.06 (s, 1 CH) 123.26 (s, 1 CH) 123.43 (s, 1 C) 127.65 (s, 1 CH) 127.88 (s, 1 CH) 128.45 (s, 1 CH) 128.92 (s, 1 CH) 135.91 (s, 1 C) 139.28 (s, 1 C) 140.19 (s, 1 C) 141.03 (s, 1 C) 144.39 (s, 1 C) 167.33 (s, 1 C).

MALDI-TOF-MS (m/z): Calculated for $C_{68}H_{64}N_2O_8$: 963; found: 963 $[M-C_4H_9OH]^+$.

Elemental analysis: Calculated: C: 78.47%; H: 6.22%; N: 2.7%; found: C: 77.88%; H: 6.117%; N: 2.79%.

9,9'-([1,1':4',1'':4'',1'''-Quaterphenyl]-4,4'''-diyl)bis(9H-carbazole-3,6-dicarboxylic acid)

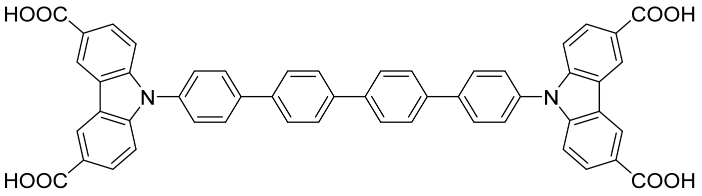

Synthesis conditions: 1.95 g (1.88 mmol) ester **17**, 1.4 g (24.6 mmol) potassium hydroxide, 120 ml THF, 2 ml methanol, 5 ml H_2O, 85 °C for 3 d; Yield: 1.5 g (98%) white powder.

^{1}H NMR (500 MHz, DMSO-d_6) δ (ppm): 7.53 (d, *J*=8.83 Hz, 1 H) 7.78 (d, *J*=8.20 Hz, 1 H) 7.93 (s, 2 H) 8.07 (d, *J*=8.51 Hz, 1 H) 8.12 (dd, *J*=8.51, 1.58 Hz, 1 H) 8.99 (s, 1 H) 12.73 (s, 1 H).

^{13}C NMR (126 MHz, DMSO-d_6) δ (ppm): 110.12 (s, 1 CH) 122.66 (s, 1 C) 123.14 (s, 1 CH) 123.54 (s, 1 C) 127.38 (s, 1 CH) 127.53 (s, 1 CH) 127.56 (s, 1 C) 128.44 (s, 1 CH) 128.53 (s, 1 CH) 135.22 (s, 1 C) 138.31 (s, 1 C) 138.94 (s, 1 C) 139.69 (s, 1 C) 143.53 (s, 1 C) 167.75 (s, 1 C).

Elemental analysis: Calculated ($C_{52}H_{32}N_2O_8$ 2.85·H_2O): C: 72.27%; H: 4.4%; N: 3.24%; found: C: 72.10%; H: 4.197%; N: 3.25%.

DRIFT, KBr, 298 K (cm^{-1}): 3627 (w, br), 2629 (w), 1907 (w), 1694 (s), 1630 (m), 1601 (m), 1523 (m), 1489 (s), 1401 (m), 1364 (m)1290 (s), 1250 (s), 1173 (m), 1137 (w), 1027 (w), 1003 (w), 949 (w), 907 (m), 813 (s), 770 (m), 735 (w), 725 (w), 692 (w).

Supplementary Note 2: Synthesis of microcrystalline MOF powders

In a previous study we have demonstrated that large crystals of DUT-49 show enhanced adsorption capacity and pronounced NGA[7]. Thus, the reaction conditions for the synthesis of DUT-48, -46, -50, -151 were chosen to produce crystals of average size above 2 µm, based on the reaction of DUT-49 by using acetic acid as modulator in a solvothermal reaction of ligands L_1 - L_5 at 80 °C. Because NMP was found to partially reduce Cu^{2+} if the reactions were carried out over 72 h or longer, we chose to use DMF instead. Due to the lower solubility especially of ligand L_1 and L_2 in DMF, larger amounts of DMF in comparison to NMP were used. The reaction conditions including the corresponding mean crystal size of the activated MOF powders are summarized in Supplementary Table 2.

Supplementary Table 2. Reaction conditions and corresponding mean crystal size.

Material ID	m_{ligand} (mg)	n_{ligand} (mmol)	$m_{Cu(NO3)2·3H2O}$ (mg)	$n_{Cu(NO3)2·3H2O}$ (mmol)	$V_{Acetic\ acid}$ (ml)	$n_{Acetic\ acid}$ (mmol)	*Eq.per ligand*	$V_{solvent}$ (ml)	$t_{Reaction}$ (h)	Yield (%)[a]	Mean crystal size/ RSD (nm)
DUT-46	800	1.26	760	3.15	16	279.7	222	400	48	83.8	2488±1709
DUT-48	1000	1.71	1030	4.26	12	209.8	123	800	72	82.7	4065±2194
DUT-49	1000	1.53	935	3.86	7.5	240	150	172	44	39.3	3488±1574
DUT-50	500	0.68	410	1.70	7.75	135.5	199	150	48	63.4	8016±6048
DUT-151	400	0.49	480	1.99	4	69.9	142	95	48	73.8	9380±3858

[a] yield based on L_3 after supercritical activation. Actual yields of reaction may be higher, [b] NMP used instead of DMF due to solubility limitations.

To obtain crystals with sizes large enough for single crystal X-ray diffraction (> 90 µm) we adapted the reaction conditions by diluting the reaction mixture with DMF and increasing the amount of acetic acid acting as modulator. The reaction conditions are summarized in Supplementary Table 3.

Supplementary Table 3. Synthesis conditions.

**Material ID**	***m*$_{Ligand}$ (mg)**	***n*$_{Ligand}$ (µmol)**	***m*$_{Cu(NO3)2·3H2O}$ (mg)**	***n*$_{Cu(NO3)2·3H2O}$ (µmol)**	***V*$_{Acetic\ acid}$ (µl)**	***n*$_{Acetic\ acid}$ (mmol)**	***Eq.per ligand***	***V*$_{DMF}$ (ml)**	***t*$_{Reaction}$ (d)**	**Crystal shape and size**
DUT-46	10	15.8	9.5	39.3	0.27	4.7	299	4	2	Cubes, 90 µm
DUT-48	10	17.1	9.8	40.6	0.11	1.9	112	5	3	Cubes, 90 µm
DUT-49	10	15.1	9.5	39.3	0.5	8.7	577	4	5	Cubes, 130 µm
DUT-50	10	13.6	8.2	33.9	0.16	2.8	206	4	3	Cuboctahedra, 110 µm
DUT-151*int*	10	12.3	7.4	30.6	0.14	2.4	199	4	1	Cubes, 100 µm

After the solvothermal reaction the blue precipitates were separated from the reaction solution by centrifugation and washed 6 times with fresh DMF over a period of two days at room temperature. DMF was exchanged with anhydr. acetone by washing 10 times over a period of at least four days. All samples were subjected to an activation procedure involving supercritical CO_2, as previously described for DUT-49[8]: In acetone suspended samples were placed on filter frits in a Jumbo Critical Point Dryer 13200J AB (SPI Supplies) which was subsequently filled with liquid CO_2 (99.995% purity) at 15 °C and 50 bar. To ensure a complete substitution of acetone by CO_2, the liquid in the autoclave was exchanged with fresh CO_2 18 times over a period of 5 days. Temperature and pressure were then raised beyond the supercritical point of CO_2 (35 °C and 100 bar) and kept until the temperature and pressure were constant at least for 1 h. Supercritical CO_2 was slowly released over 3 h and the dry powder was transferred and stored in an argon filled glove box. To ensure complete removal of the solvent from the pores (especially from the open metal sites of the Cu-paddle-wheels) additional thermal activation at 120 °C in a Schlenk-tube under dynamic vacuum of 10^{-4} kPa for at least 24 h was performed.

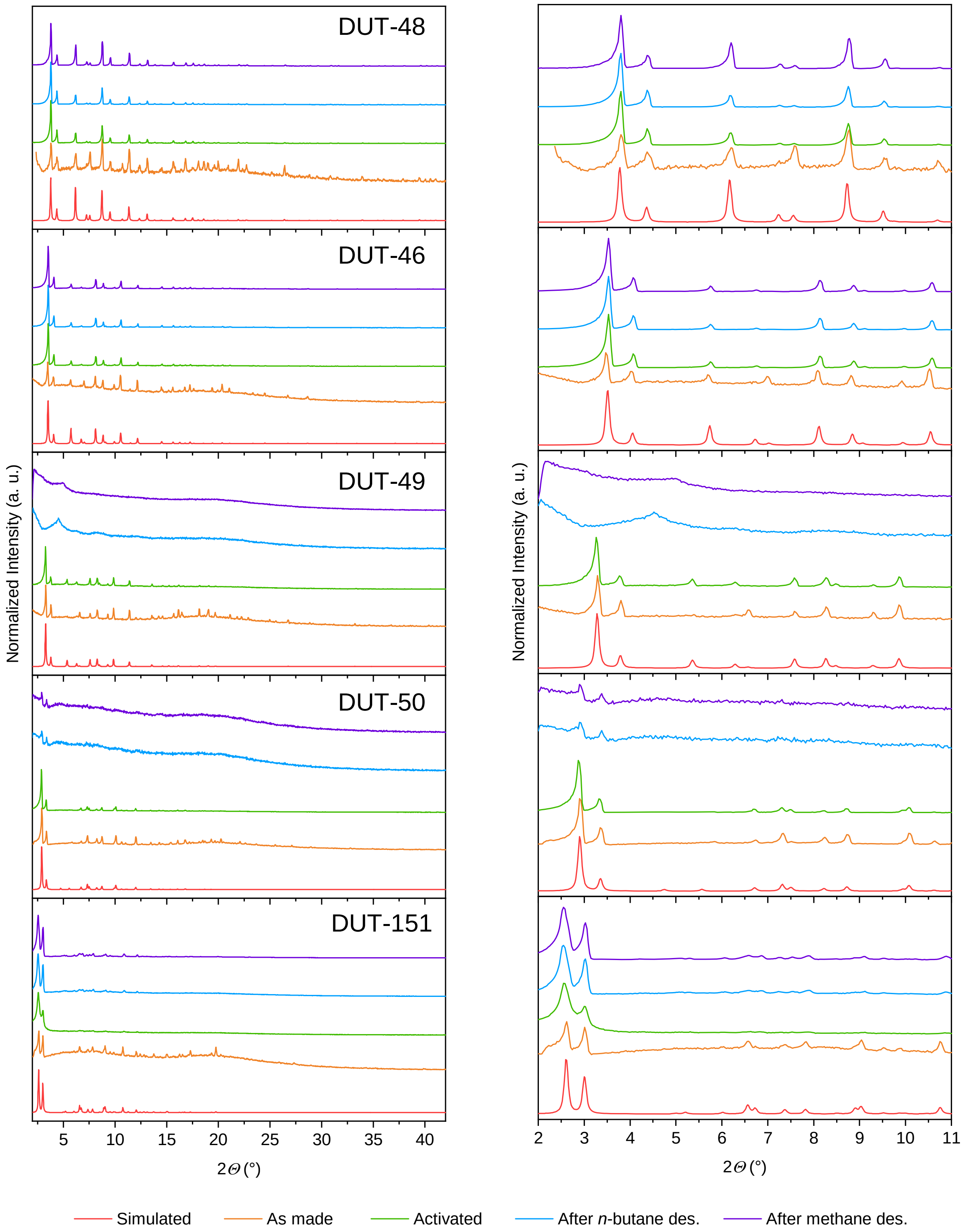

Supplementary Figure 5. PXRD patterns from top to bottom for DUT-48, -46, -49, -50, -151*int*. Left column: wide angle and on the right corresponding magnified regions.

Supplementary Note 3: Single crystal X-ray diffraction analysis of DUT-46, DUT-50, DUT-151*int*

Blue cubic single crystals of DUT-46, DUT-50 and DUT-151 with dimensions ranging from 30 µm to 80 µm were prepared in a borosilicate glass capillary (d = 0.3 mm) with small amount of the mother liquor. The capillaries were sealed with wax from both sides in order to avoid the contact with ambient atmosphere. The datasets were collected at BESSY MX BL14.3 beamline of Helmholtz-Zentrum Berlin für Materialien und Energie[9]. Monochromatic X-ray radiation with a wavelength of λ = 0.089499 nm (E = 13.85 keV) was used in experiments. All datasets were collected at room temperature. After short test scans, the crystal symmetry and scan range were determined in each particular case using iMosflm program.[10,11] The φ-scans with oscillation range of 0.5° were used for data collection. In the case of cubic structures of DUT-46 and DUT-50, 100 images were collected to reach the maximal completeness. For DUT-151, crystallizing in *C*-centered monoclinic lattice, 240 images were required. Further the datasets were processed automatically using XDSAPP 2.0 software.[12] Crystal structures were solved by direct methods and refined by full matrix least-squares on F^2 using SHELX-2016/4 program package.[13,14] All non-hydrogen atoms were refined in anisotropic approximation. Hydrogen atoms were refined in geometrically calculated positions using "riding model" with $U_{iso}(H)=1.2U_{iso}(C)$. Since the symmetry of the naphthalene core in DUT-46 is incompatible with the symmetry of its position in the unit cell, the molecular fragment was refined disordered over four symmetrically dependent positions. In the case of DUT-50, the disorder of both symmetrically independent phenyl rings was treated by splitting over two equally occupied positions. The lower symmetry of DUT-151*int*, with four symmetrically independent paddle wheels in the asymmetric unit prompted us to use the AFIX 66, SIMU and DELU instructions in order to constrain the geometry and anisotropic displacement parameters of all phenyl rings in the structure. The large pores, high crystal symmetry and high measurement temperature did not allow refining the disordered solvent molecules within the pores of the frameworks, therefore, SQUEEZE routine in PLATON was used to generate the reflection intensities with subtracted solvent contribution.[15] CCDC-1889257, 1889255 and 1889256 contain the supplementary crystallographic data for DUT-46, DUT-50 and DUT-151*int*, correspondingly. These data can be obtained free of charge from the Cambridge Crystallographic Data Centre via www.ccdc.cam.ac.uk/data_request/cif.

Supplementary Table 4. Single crystal data of DUT-46, DUT-50 and DUT-151*int*

	DUT-46	DUT-50	DUT-151*int*
Empirical formula	$C_{38} H_{18} O_{10} N_2 Cu_2$	$C_{46} H_{24} O_{10} N_2 Cu_2$	$C_{52} H_{28} O_{10} N_2 Cu_2$
Formula weight	789.66	891.75	967.84
Crystal system, space group	cubic, $Fm\bar{3}m$	cubic, $Fm\bar{3}m$	monoclinic, $C2/m$
Unit cell dimensions, *Å* or deg	a = 43.550(5)	a = 52.630(6)	a = 71.700(14)<br>b = 41.570(8)<br>c = 41.450(8)<br>β = 125.08(3)
Unit cell volume, $Å^3$	82597(28)	145781(51)	101103(46)
Z	24	24	24
Calculated density, $g\ cm^{-3}$	0.381	0.244	0.382
Absorption coefficient, 1/*mm*	0.604	0.345	0.500
F(000)	9536 (after SQUEEZE)	10848 (after SQUEEZE)	11808 (after SQUEEZE)
θ range, °	1.02 – 37.08	0.84 – 25.48	0.76 – 33.99
Limiting indices	$-32 \leq h \leq 56$<br>$-51 \leq k \leq 55$<br>$-58 \leq l \leq 17$	$-47 \leq h \leq 49$<br>$-50 \leq k \leq 45$<br>$-48 \leq l \leq 19$	$-88 \leq h \leq 25$<br>$-31 \leq k \leq 51$<br>$-46 \leq l \leq 51$
Reflections collected / unique	52664 / 4900	34672 / 3320	100606 / 71100
R(int)	0.0501	0.1122	0.0565
Data / parameters	4900 / 102	3320 / 110	71100 / 1574
GooF on F^2	1.621	1.111	1.034
Final R indices [I>2σ(I)]	R_1 = 0.0587<br>wR_2 = 0.1948	R_1 = 0.0597<br>wR_2 = 0.1864	R_1 = 0.0622<br>wR_2 = 0.1693
R indices (all data)	R_1 = 0.0804<br>wR_2 = 0.2198	R_1 = 0.0963<br>wR_2 = 0.2267	R_1 = 0.1018<br>wR_2 = 0.1942
Largest diff. peak / hole, $eÅ^{-3}$	0.52 / -0.35	0.47 / -0.36	0.37 / -0.30

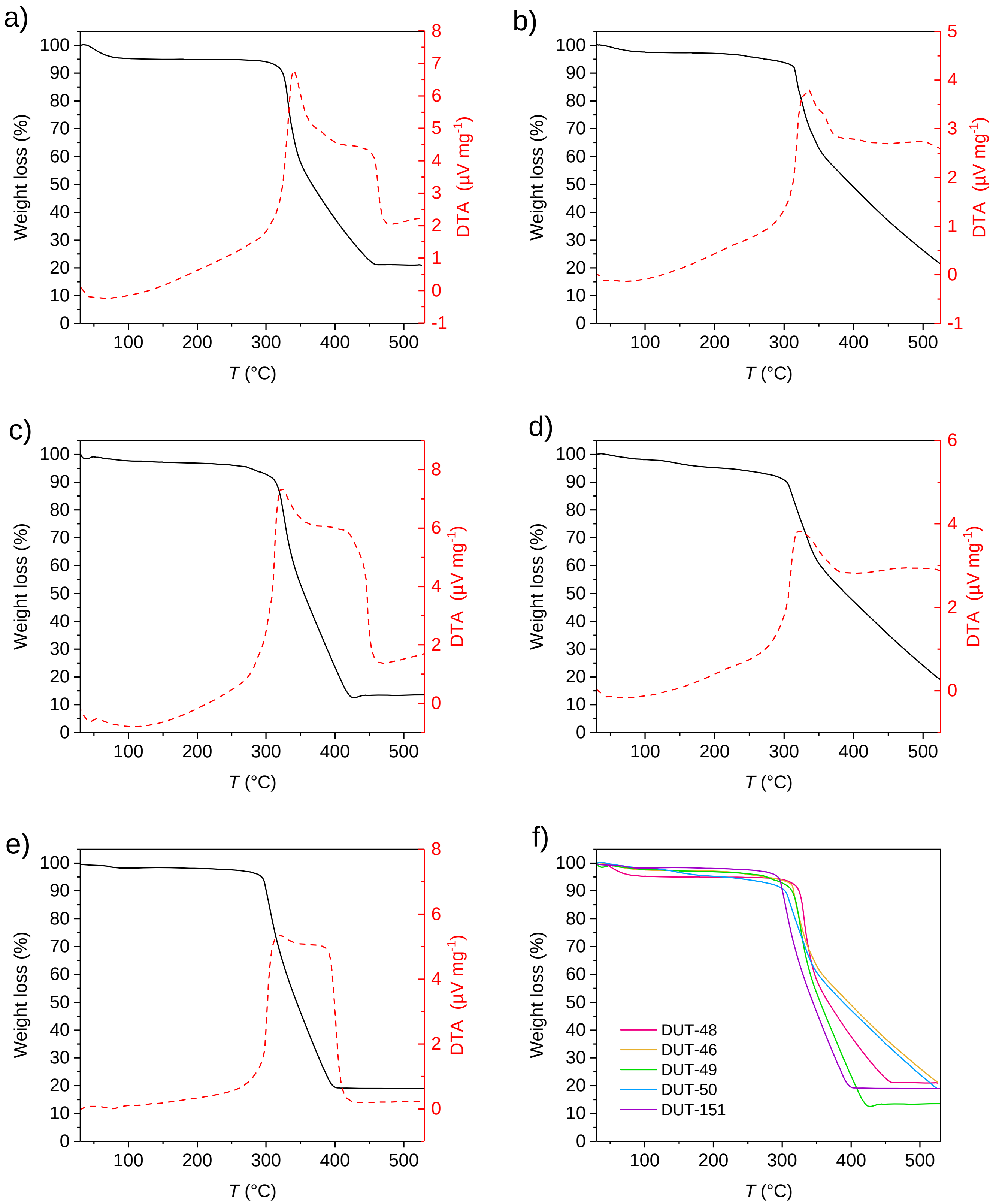

Supplementary Figure 6. Thermogravimetric analysis of a) DUT-48, b) DUT-46, c) DUT-49, d) DUT-50, e) DUT-151*int* and f) comparison of all materials.

Supplementary Table 5. CHN elemental analysis for activated MOFs.

	Calculated (%)			Experimental (%)		
Material ID	C	H	N	C	H	N
DUT-46	60.24	2.39	3.7	59.06	2.33	3.81
DUT-48	57.71	2.28	3.96	56.99	2.403	3.98
DUT-49	61.3	2.57	3.57	59.85	2.542	3.64
DUT-50	64.26	2.81	3.26	62.82	3.549	3.45
DUT-151*int*	66.73	3.02	2.99	66.58	3.077	3.37

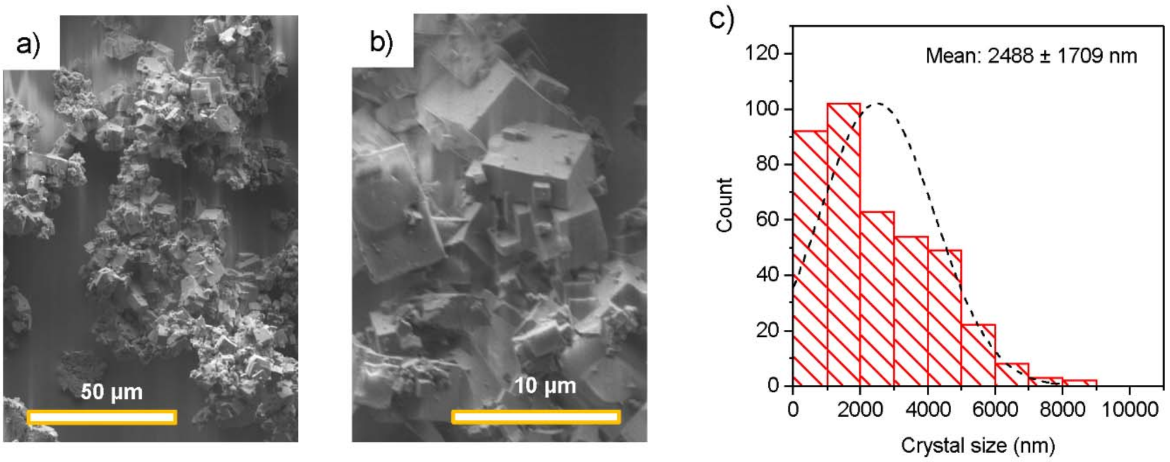

Supplementary Figure 7. a-b) Scanning electron microscopy images of DUT-48, scale bar a) 50 µm, b) 10 µm. c) Experimental crystal size distribution (red histogram) and distribution curve (black dashed line).

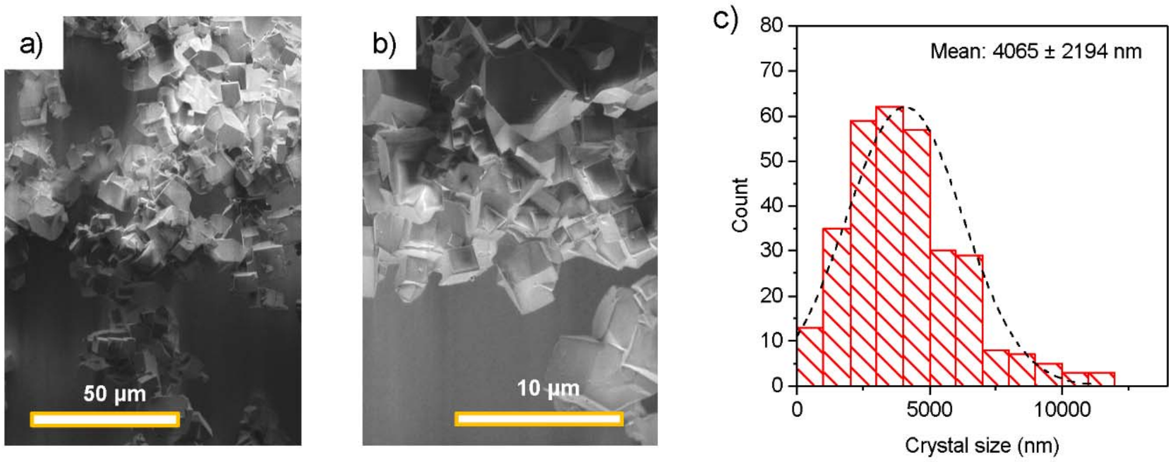

Supplementary Figure 8. a-b) Scanning electron microscopy images of DUT-46, scale bar a) 50 µm, b) 10 µm. c) Experimental crystal size distribution (red histogram) and distribution curve (black dashed line).

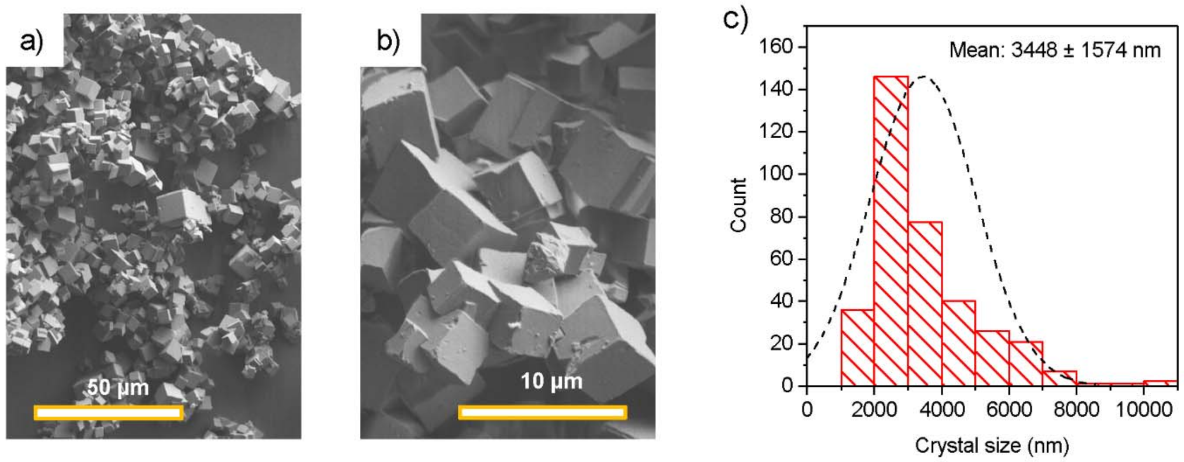

Supplementary Figure 9. a-b) Scanning electron microscopy images of DUT-49, scale bar a) 50 µm, b) 10 µm. c) Experimental crystal size distribution (red histogram) and distribution curve (black dashed line).

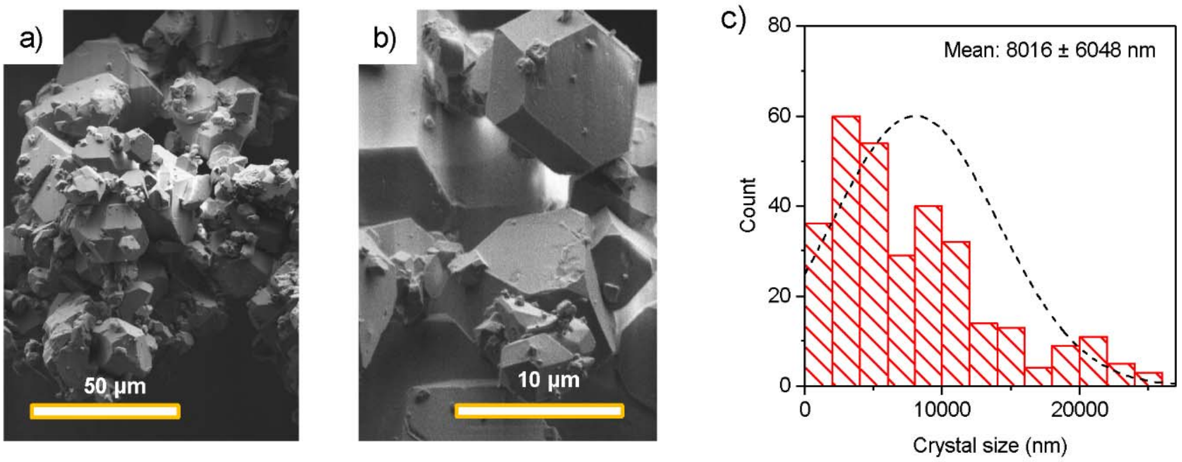

Supplementary Figure 10. a-b) Scanning electron microscopy images of DUT-50, scale bar a) 50 µm, b) 10 µm. c) Experimental crystal size distribution (red histogram) and distribution curve (black dashed line).

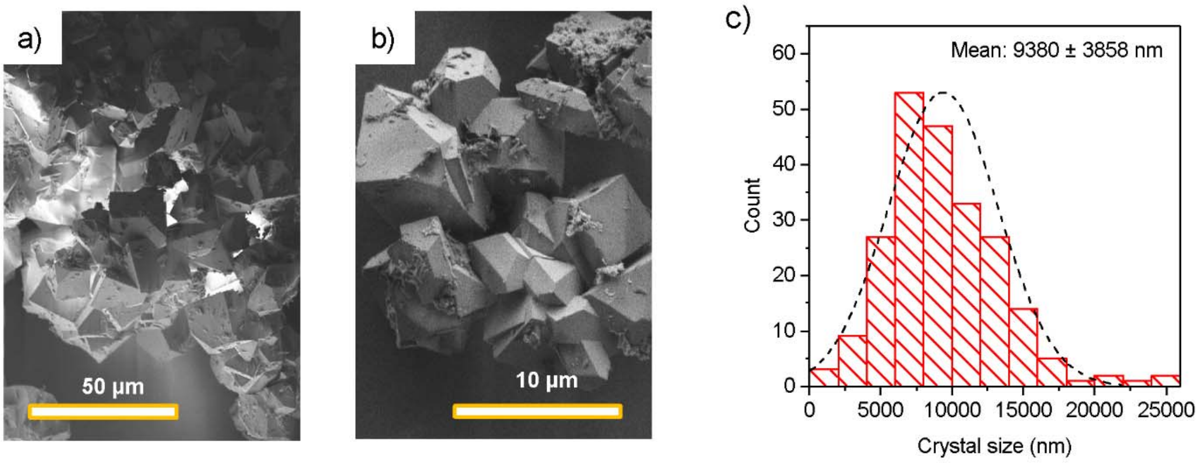

Supplementary Figure 11. a-b) Scanning electron microscopy images of DUT-151*int*, scale bar a) 50 µm, b) 10 µm. c) Experimental crystal size distribution (red histogram) and distribution curve (black dashed line).

Supplementary Table 6. Crystal size distribution extracted from SEM analysis.

Material ID	Mean crystal size (nm)	Standard deviation (nm)	Relative standard deviation (%)
DUT-48	2488	1709	68.7
DUT-46	4065	2194	53.9
DUT-49	3488	1574	45.1
DUT-50	8016	6048	75.4
DUT-151*int*	9380	3858	41.1

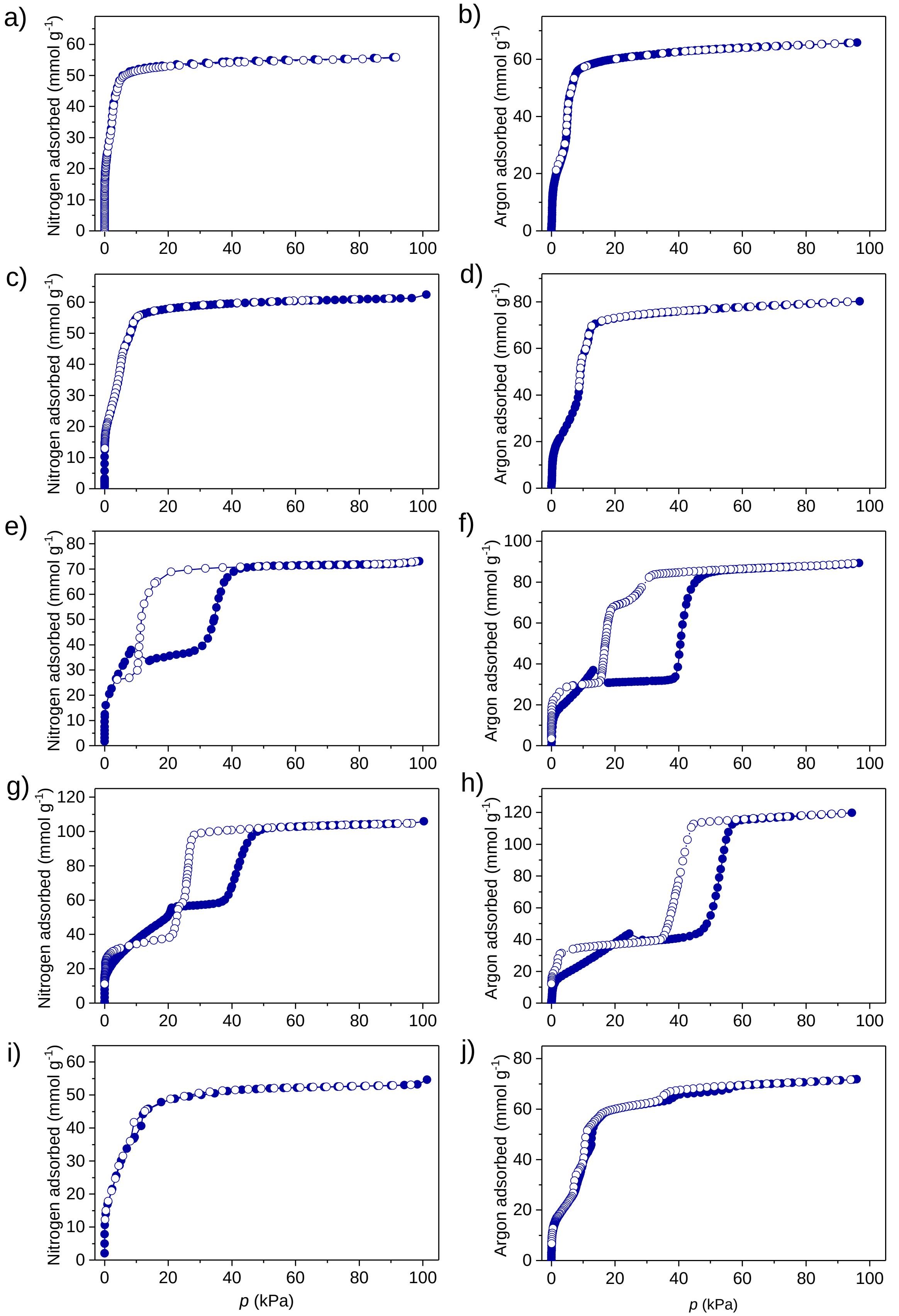

Supplementary Figure 12. Nitrogen at 77 K (left) and argon at 87 K (right) physisorption isotherms of DUT-48 (a,b), DUT-46 (c,d), DUT-49 (e,f), DUT-50 (g,h), DUT-151 (i,j). Filled symbols: adsorption, empty symbols: desorption.

Supplementary Note 4: Textural analysis of microcrystalline MOF powders

Textural properties, such as geometric surface area, pore volume and pore size distributions (PSD), were calculated for these structures using the Zeo++ code[16] on the basis of disorder-free simulated crystal structures for *op* phases and *in silico* determined crystal structures for the corresponding *cp* phases for DUT-48, -151, and experimental DUT-151*int* structure. The obtained results were compared to experimentally obtained values derived from nitrogen and argon adsorption isotherms at 77 K and 87 K, respectively.

Supplementary Table 7. Comparison of experimental and geometrical surface area and pore volumes.

Material ID	Pore Volume (N_2 ads.) (cm^3 g^{-1})	Pore Volume (Ar ads.) (cm^3 g^{-1})	Geometric surface area (m^2 g^{-1})	Geometric pore volume (cm^3 g^{-1})	Framework density (g cm^{-3})
DUT-48	1.98	1.88	4394	1.67	0.424
DUT-46	2.15	2.29	4736	1.89	0.365
DUT-49	2.73	2.81	5065	2.64	0.306
DUT-50	3.52	3.41	5386	3.45	0.235
DUT-151*int*	1.95	2.05	4688	1.81	0.368
DUT-151	n. a.	n. a.	5672	4.43	0.183

In general, geometric values for pore volume and surface area are expected to be inferior to experimental values. From the experimental adsorption data, accurate determination of specific surface areas for the investigated materials was not possible by using the BET method fulfilling all consistency criteria recently defined for the analysis of mesoporous MOFs[17]. Several linear regions in the BET-plot were analysed and although all of them exhibit a good correlation coefficient only a few of them (marked red in Supplementary Table 7) were found to fulfil all consistency criteria recommended for determination of specific surface area by the BET method. Mostly, the third criterion is not fulfilled: the relative pressure value (p/p_0) corresponding to the monolayer capacity (n_m) should be located within the selected BET range.

Only for DUT-151*int* a specific BET surface area in the range of the geometric surface area determined from the guest-free crystal structures was obtained. This might be due to the smaller pore size and microporous nature of DUT-151*int* in contrast to the mesoporous character of the other materials in this series. The only surface area observed to be within the selection criteria for DUT-50 is found to be drastically lower than the geometric surface area [18]. Due to the observed disagreement of experimental and simulated surface area, only the geometric surface area is considered for the following discussions.

Supplementary Table 8. BET analysis of argon adsorption isotherms of DUT-46, -48, -50, and DUT-151. Values printed in bold fulfill selection criteria for accurate experimental determination of specific surface area by the BET method.

Material ID	Relative pressure range	Slope	Intercept	Correlation coefficient R^2	BET constant, C	n_m (mol g^{-1})	p/p_0 n_m	BET surface area (m^2 g^{-1})
DUT-48	0.007519 – 0.0112	1.05205	0.00425	0.9994	248	0.0236	0.059	2026
	0.01718 – 0.02553	0.83839	0.00715	0.9994	118	0.0296	0.084	2531
	0.02854 – 0.03448	0.61778	0.01301	0.9989	49	0.0397	0.126	3393
	0.03587 – 0.03981	0.4329	0.0195	0.9986	23	0.0553	0.172	4731
	0.05707 – 0.06591	0.3001	0.0152	0.9999	20	0.0799	0.180	6791
DUT-46	0.009669 – 0.0234	0.9980	0.005699	0.9998	176	0.0249	0.070	2133
	0.02528 – 0.04116	0.780784	0.010995	0.9992	72	0.0316	0.110	2704
	0.09791 - 0.10996	0.2319	0.0257	0.9999	10	0.0971	0.240	8310
DUT-151*int*	0.00019 – 0.00709	1.5573	0.0027	0.9999	578	0.016	0.040	1372
	0.00981 – 0.0244	1.1950	0.0060	0.9995	201	0.020	0.066	1782
	0.00272 – 0.0419	0.9522	0.0122	0.9991	79	0.0259	0.101	2219
	0.0448 – 0.0635	0.7228	0.0222	0.9983	34	0.0336	0.147	2873
	0.104 – 0.115	0.4004	0.02971	0.9998	15	0.058	0.208	4977
	**0.14 – 0.164**	0.4279	0.01423	0.9999	31	0.0566	**0.152**	**4841**
DUT-50	0.00432 – 0.00639	1.1761	0.00703	0.9991	168	0.0211	0.072	1809
	0.00695 – 0.0112	1.5407	0.0048	0.9994	321	0.0169	0.053	1385
	0.0655 – 0.108	0.776	0.0341	0.9976	23	0.0309	0.170	2642
	0.119 – 0.17	0.5368	0.06909	0.9986	9.8	0.0419	0.240	3581
	**0.0182 – 0.245**	0.4553	0.07436	0.9999	62	0.0541	**0.113**	**4626**

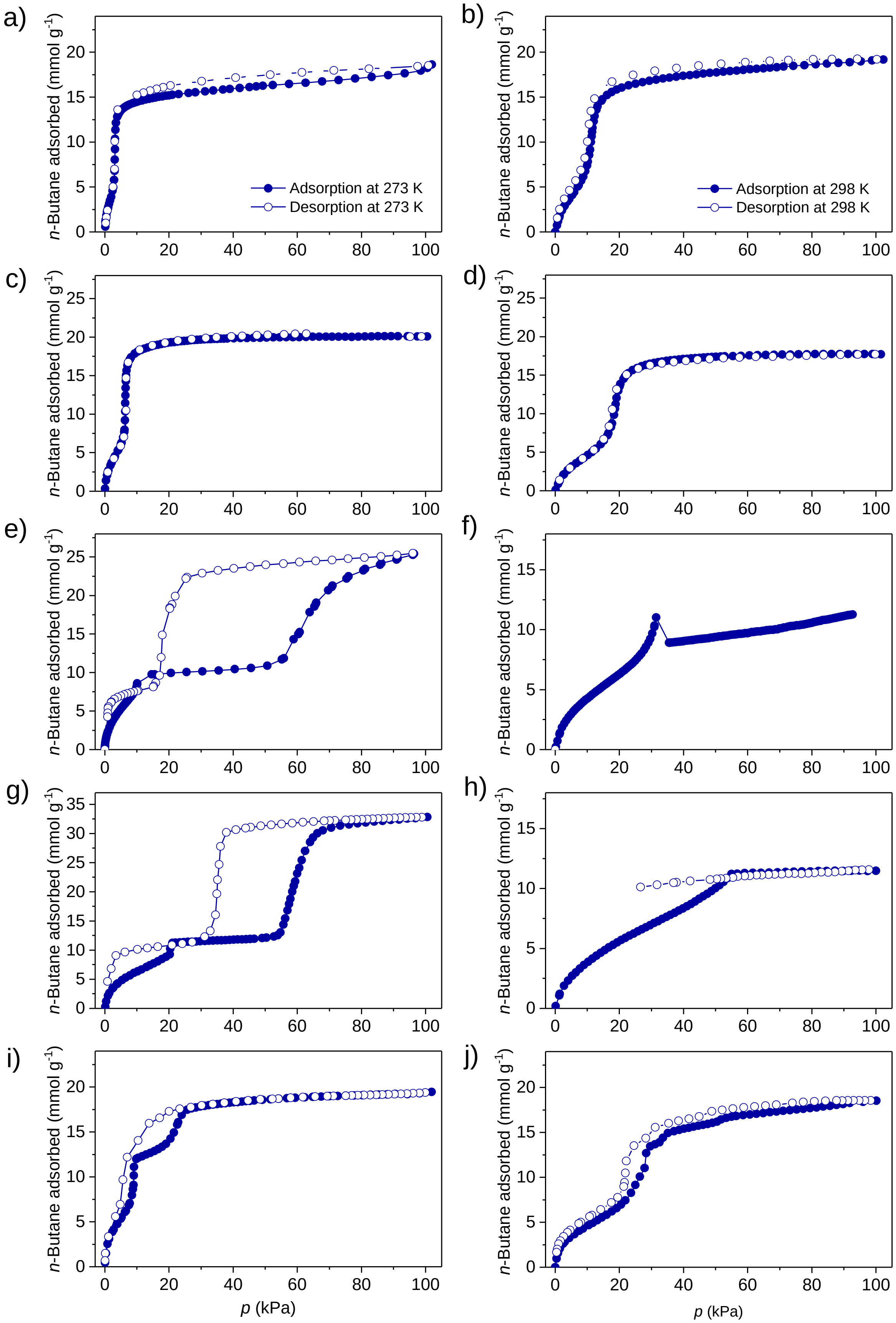

Supplementary Figure 13. *n*-Butane adsorption isotherms at 273 K (left) and 298 K (right) of DUT-48 (a,b), DUT-46 (c,d), DUT-49 (e,f), DUT-50 (g,h), DUT-151 (i,j). Filled symbols: adsorption, empty symbols: desorption.

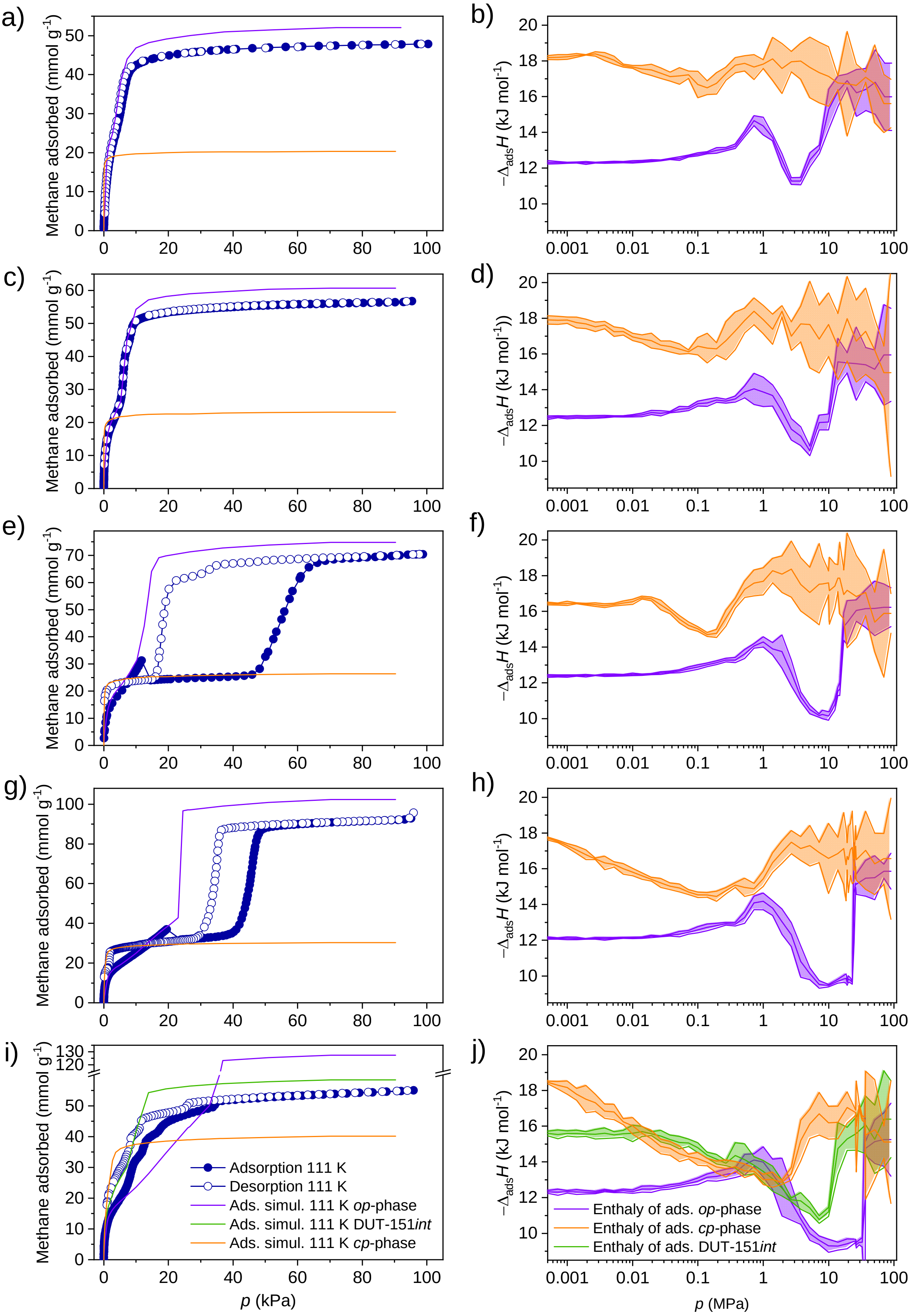

Supplementary Figure 14. Methane isotherms at 111 K and corresponding simulated enthalpy profile of corresponding *op* and *cp* phases for DUT-48 (a,b), DUT-46 (c,d), DUT-49 (e,f), DUT-50 (g,h), and DUT-151 (i,j). Filled symbols: adsorption, empty symbols: desorption.

Supplementary Table 9. NGA parameters for DUT-49 and DUT-50.

Gas	T (K)	DUT-49 Δn_{NGA} (mmol g^{-1})*	DUT-49 p_{Trans} (kPa)	DUT-50 Δn_{NGA} (mmol g^{-1})*	DUT-50 p_{Trans} (kPa)
Nitrogen	77	4.41	8.32	0	21.14
Argon	87	6.13 (+0.02)	13.12	4.03 (+0.02)	24.5
Methane	111	7.24 (+0.05)	11.72	5.17 (+0.03)	19.52
n-Butane	298	1.89 (+0.05)	31.46	0	54.98
n-Butane	303	0.74 (+0.5)	38.21	0.14 (+0.5)	65.2

*The accuracy of determination of Δn_{NGA} depends on the resolution of the isotherm. The errors provided in Supplementary Table 9 represent the step size and give an indication of what the maximum Δn_{NGA} could be for higher resolution isotherm.

Supplementary Note 5: Adsorption microcalorimetry analysis of microcrystalline MOF powders

For microcalorimetry, all isotherms and enthalpies were measured experimentally using a Tian-Calvet type microcalorimeter coupled with a home-made manometric gas dosing system[19]. This apparatus allows the simultaneous measurement of the adsorption isotherm and the corresponding differential enthalpies. Gas is introduced into the system using a step-by-step method and each dose is allowed to stabilize in a reference volume before being brought into contact with the adsorbent located in the microcalorimeter. The introduction of the adsorbate to the sample is accompanied by an exothermic thermal signal, measured by the thermopiles of the microcalorimeter. The peak in the calorimetric signal is integrated over time to give the total energy released during this adsorption step. Around 0.05 g of sample is used in each experiment. For each injection of gas, equilibrium was assumed to have been reached after 130 minutes. This was confirmed by the return of the calorimetric signal to its baseline (< 5 μW). The gases used for the adsorption experiment were obtained from Air Liquide and were of minimum N47 quality (99.997 % purity).

To obtain the experimental errors, the procedure described in the *Guide to the Expression of Uncertainty in Measurement* was used. The quantity, enthalpy or pressure, is first expressed as a function $f(y)$ of other physical measured quantities. The standard uncertainty ($u_c(y)$) is then calculated on the basis of, where $u_i(x_i)$ is the standard uncertainty in each input quantity. Here it is assumed that the input quantities are independent and uncorrelated. The error margins ($\mathrm{a_i}$) for each quantity were taken from manufacturer specifications of the equipment used for recording. They were then divided by a value k_i chosen to cover the expected variance in that quantity, as each variable is assumed to be characterized by a probability distribution. The error introduced by the equation of state used (NIST REFPROP[20]) were assumed to be minor compared to the error introduced by the physical quantities, with the same to be said regarding the error in the calorimetric heat signal, which represents less than 1% of the error in enthalpy. Finally, the expanded uncertainty was calculated by choosing a suitable coverage factor of 1.645, corresponding to a 95% confidence interval.

$$f(y) = f(N_1, N_2 \dots N_i) \tag{1}$$

$$u_c(y) = \sqrt{\sum_{i=1}^{N} \left(\frac{\partial f(y)}{\partial x_i} \times u_i(x_i) \right)^2} \tag{2}$$

Supplementary Table 10. Parameters used to estimate the errors of *in situ* calorimetric analysis.

Variable (i)	$\mathrm{a_i}$	k_i
Pressure, p (Pa)	20	$\sqrt{3}$
Temperature, T (K)	0.1	3
Sample mass, m (g)	1×10^{-4}	$\sqrt{3}$
Reference volume, V_r (m^3)	1×10^{-9}	$\sqrt{6}$
Cell volume, V_c (m^3)	6×10^{-7}	$\sqrt{6}$

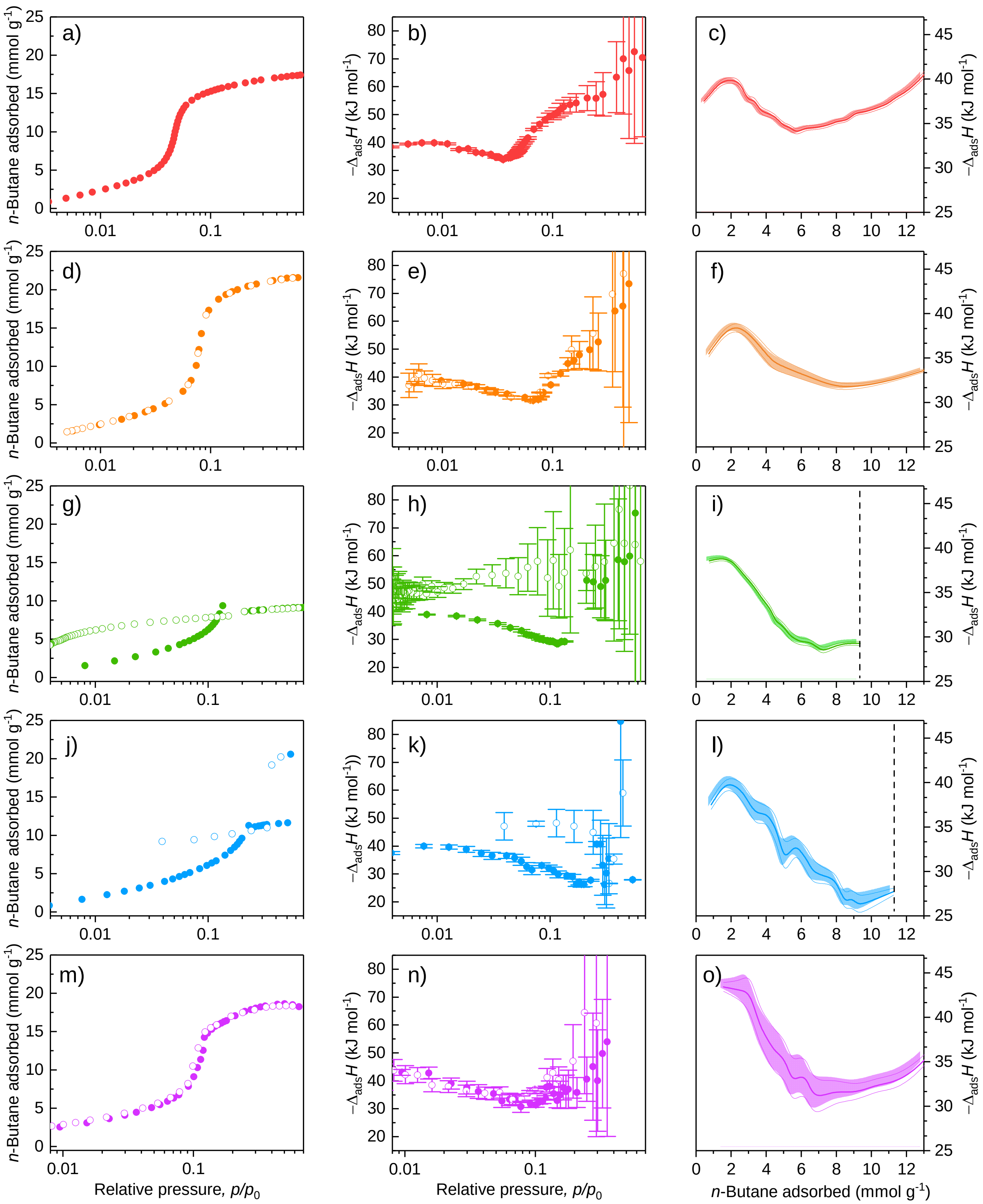

Supplementary Figure 15. Adsorption (filled symbol) and desorption (empty symbol) isotherm of *n*-butane at 303 K (left), corresponding adsorption/desorption enthalpies and error bars (middle), and isosteric adsorption enthalpy profiles with semi-transparent error region (right) of a-c) DUT-48, d-f) DUT-46, g-i) DUT-49, j-l) DUT-50, m-o) DUT-151*int*. Dashed lines in i) and l) indicate NGA transition and enthalpies beyond this loading were removed for clarity.

In DUT-46 and DUT-151*int* small hystereses were observed in the enthalpy profile. In the case of DUT-151*int* this can be correlated to a structural transition analysed by in situ PXRD and reflected by a hysteresis in the adsorption isotherm. No hysteresis in the adsorption/desorption isotherms and no structural transition can be observed in the pressure range in which a hysteresis is observed in the enthalpy profile.

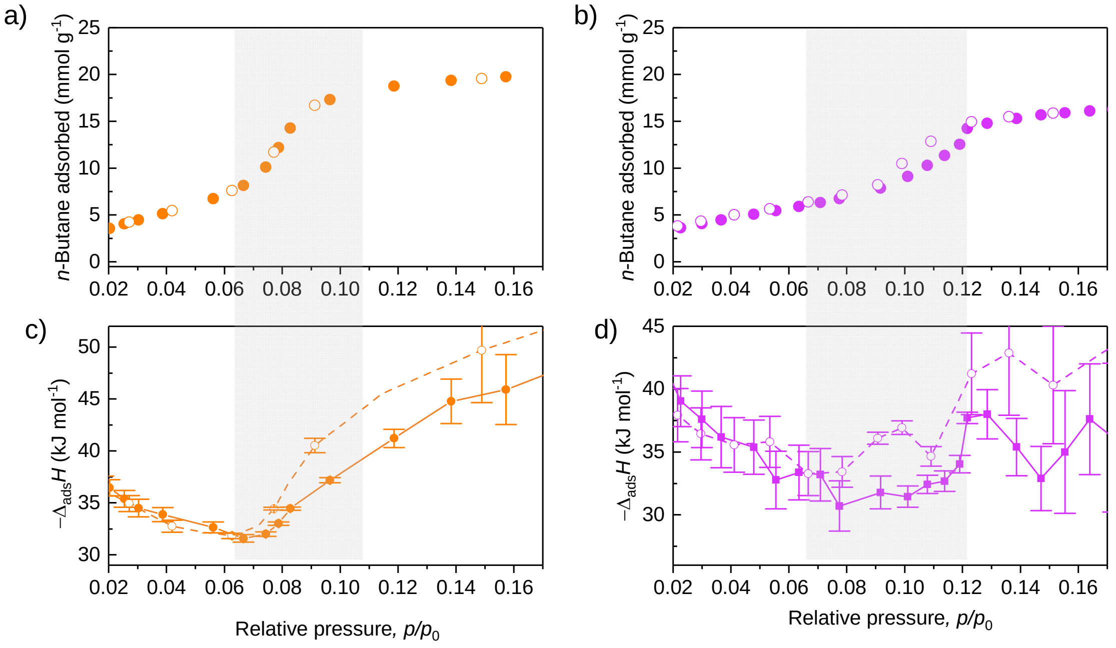

Supplementary Figure 16. Adsorption (filled symbol) and desorption (empty symbol) isotherm of *n*-butane at 303 K for a) DUT-46 and b) DUT-151*int* with corresponding adsorption/desorption enthalpies and error bars for c) DUT-46 and d) DUT-151*int*. Grey regions indicate hysteresis in enthalpy profiles.

Supplementary Note 6: Mercury intrusion porosimetry analysis of microcrystalline MOF powders

Mercury intrusion is a method frequently applied to investigate the mechanical properties and structural contraction of metal organic frameworks.[21-26] To begin with, the solids were activated at 115°C for 8 h in secondary vacuum. The obtained powder was then loaded into a powder penetrometer of 3.1126 cm^3 volume with a stem volume of 0.412 cm^3 using a glove box (Jacomex P-BOX) under argon atmosphere H_2O < 5 ppm. The mercury intrusion experiments were carried out using a Micromeritics AutoPore IV 9500 allowing a range of pressure applied from 0.003 to 300 MPa. Prior to the experiment the powder was outgassed in vacuum at ~6.5 Pa for 15 minutes. The collected volume of mercury intruded was corrected by a blank measurement recorded under the same conditions (temperature and pressure) using the same penetrometer to obtain the absolute contracted volume as a function of the pressure reported in Supplementary Figure 17. The resulting volume change upon structural contraction can be determined from the cumulative volume of mercury intruded obtained by mercury intrusion using equation (3):

$$V_{cp} = V_{op} - \frac{Z \times M \times \left(V_{final} - V_{initial}\right) \times 10^{24}}{N_A} \qquad (3)$$

where V_{cp} and V_{op} are the volumes of the contracted and open phases respectively in $Å^3$, Z the number of formula units per unit cell (in the case of the investigated materials Z = 24), M is the molar mass of a formula unit in g mol^{-1} derived from the crystal structure, V_{final} and $V_{initial}$ the cumulative volumes of mercury intruded at the end (last point of the second plateau) and at the beginning of the transition (end of the first plateau) in ml g^{-1} and N_A is Avogadro's constant in mol^{-1}.

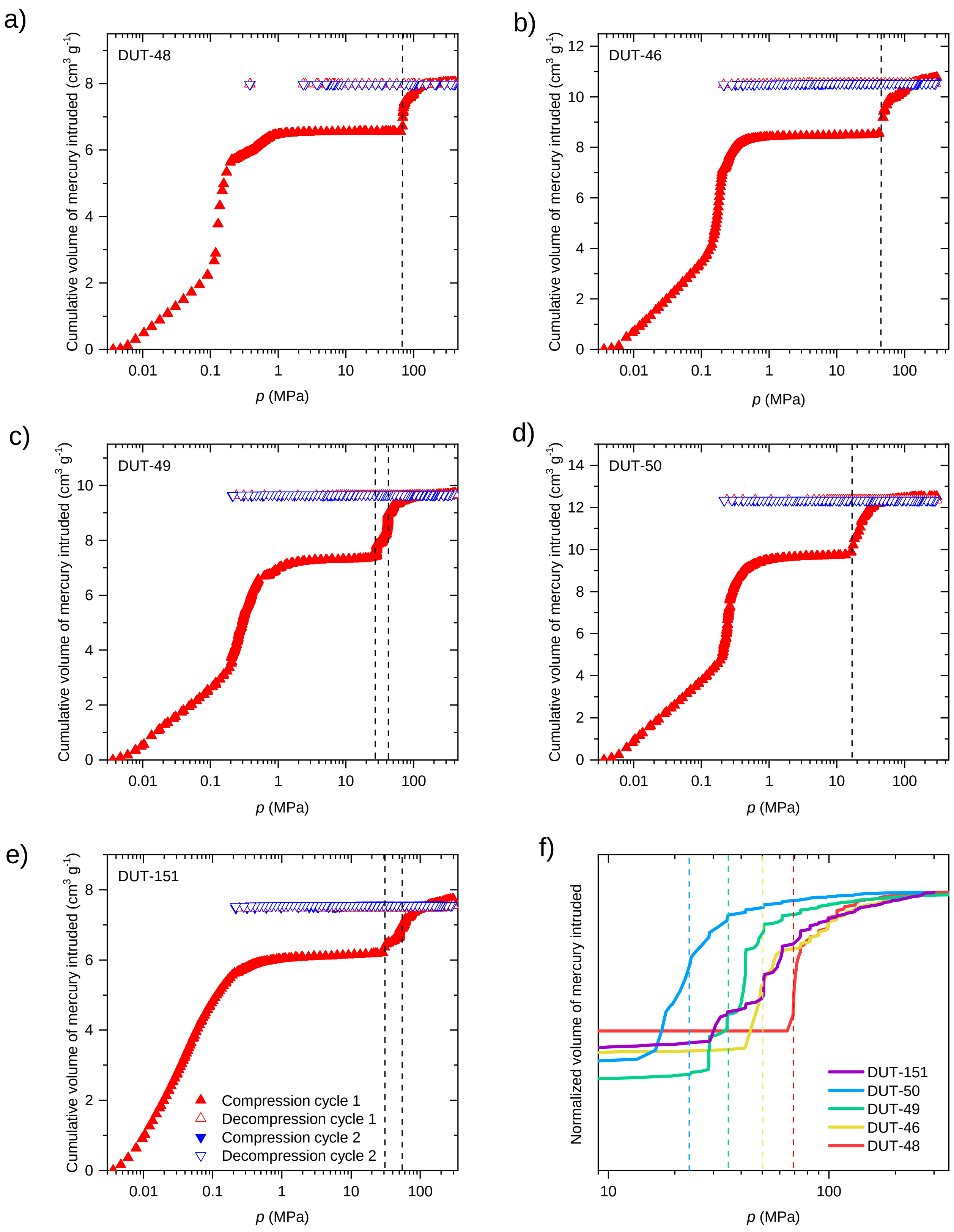

Supplementary Figure 17. Mercury intrusion curves for DUT-48 (a), DUT-46 (b), DUT-49 (c), DUT-50 (d), DUT-151 (e), and f) comparison of the intrusion curves with transition pressures indicated as dashed lines.

Supplementary Table 11. Summary of experimental details of Hg intrusion and comparison with simulated results.

	Pore volume (cm^3 g^{-1})	Volume change Hg intrusion total (cm^3 g^{-1})	Volume change Hg intrusion total (%)	ΔV simulation *op* - *cp* (%)	Transition pressure measured (MPa)	Transition pressure simulated (MPa)
DUT-48	1.98	1.5	63.7	44.0	65	145
DUT-46	2.153	2.01	77.8	47.2	50	84
DUT-49	2.89	2.30	71.8	51.4	35	65
DUT-50	4.02	2.78	65.3	59.2	24	38
DUT-151	n. a.	n. a.	n. a.	62.5	n. a.	22
DUT-151*int*	1.891	1.53	56.4	27.9	29	n. a.

Supplementary Note 7: Parallelised powder X-ray diffraction and high resolution gas adsorption studies of microcrystalline MOF powders

In situ-PXRD studies and parallelised gas adsorption were measured at KMC-2 beamline[27] of the BESSY II synchrotron, operated by Helmholtz-Zentrum Berlin für Materialien und Energie. Self-designed automated instrumentation, based on the volumetric adsorption instrument and closed-cycle Helium cryostat, equipped with adsorption chamber with beryllium domes was used in all experiments.[28] PXRD patterns were measured at constant wavelength λ = 0.15406 nm (E = 8048 eV) in transmission geometry. Because of the bulky cryostat, the sample holder cannot rotate during experiments, however an average crystallite size in the range of 2-15 µm and using an area 2D detector (Vantec 2000, Bruker) allowed to record diffraction images with reasonable particle statistics. Each 2D image was measured with 31 s exposure. For each experiment 10-12 mg of sample were used. In order to block the reflections coming from the crystalline Be-dome, a tungsten aperture with 5 mm opening was mounted on the detector cone. The obtained diffraction images were integrated using DATASQUEEZE 2.2.9[29] with further processing in FITYK 0.9[30] software. PXRD patterns, measured for DUT-50 during adsorption and desorption of methane at 111 K (Supplementary Figure 20) and *n*-butane at 273 K (Supplementary Figure 19) were obtained in the automatic mode in parallel to the isotherm measurement. The dataset of DUT-151*int* tracking the adsorption and desorption of *n*-butane at 273 K was collected in the automated modus as well (Supplementary Figure 22). For all automated measurements the physisorption isotherms were measured using equilibrium settings for pressure change of 0.1% within 300 s. The dataset during *n*-butane adsorption at 303 K (Supplementary Figure 18) as well as the analysis of DUT-151*int* at 273 K (Supplementary Figure 22) was measured in the manual mode. Each pressure was set manually and PXRD patterns were measured after the pressure in the cell was stabilized for at least 300 s.

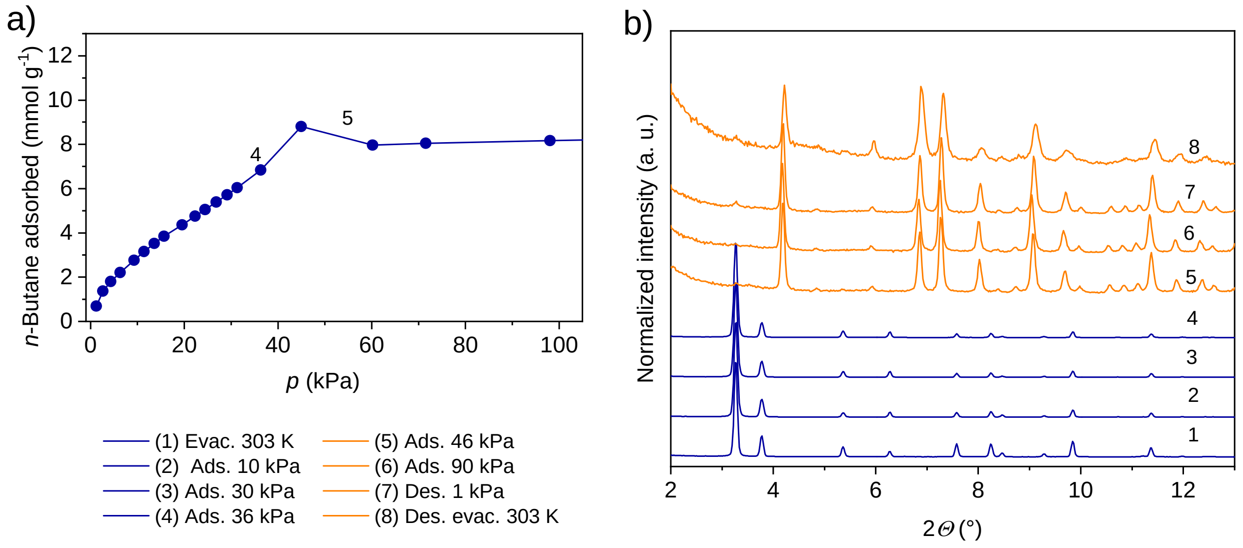

Supplementary Figure 18. *In situ*-PXRD data for DUT-49: a) *n*-butane adsorption isotherm at 303 K and corresponding PXRD patterns at varying pressures.

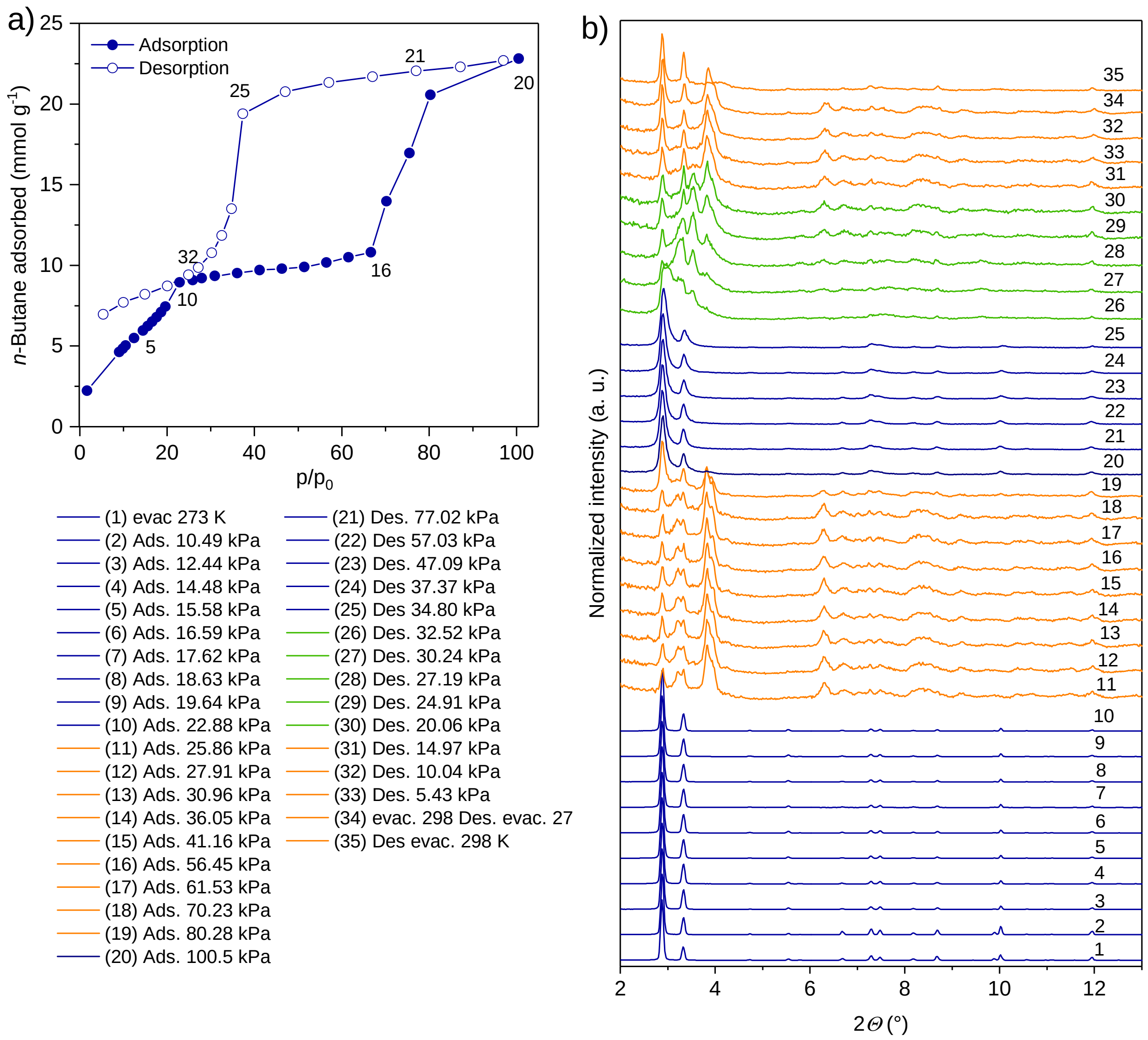

Supplementary Figure 19. DUT-50 *in situ*-PXRD dataset with a) *n*-butane isotherm at 273 K and corresponding PXRD patterns at varying pressures. Filled symbols: adsorption, empty symbols: desorption.

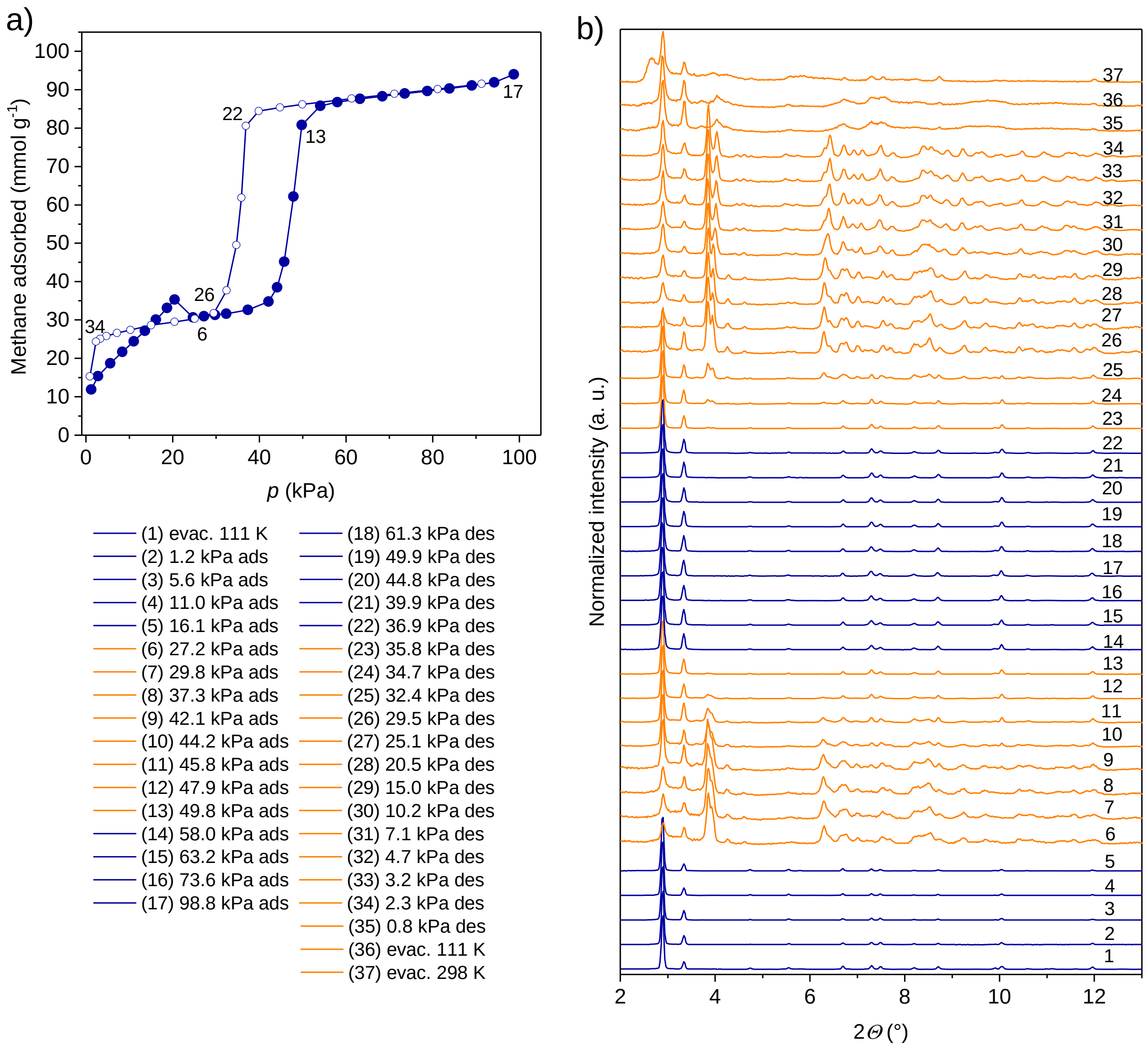

Supplementary Figure 20. DUT-50 *in situ*-PXRD dataset with a) methane isotherm at 111 K and corresponding PXRD patterns at varying pressures. Filled symbols: adsorption, empty symbols: desorption.

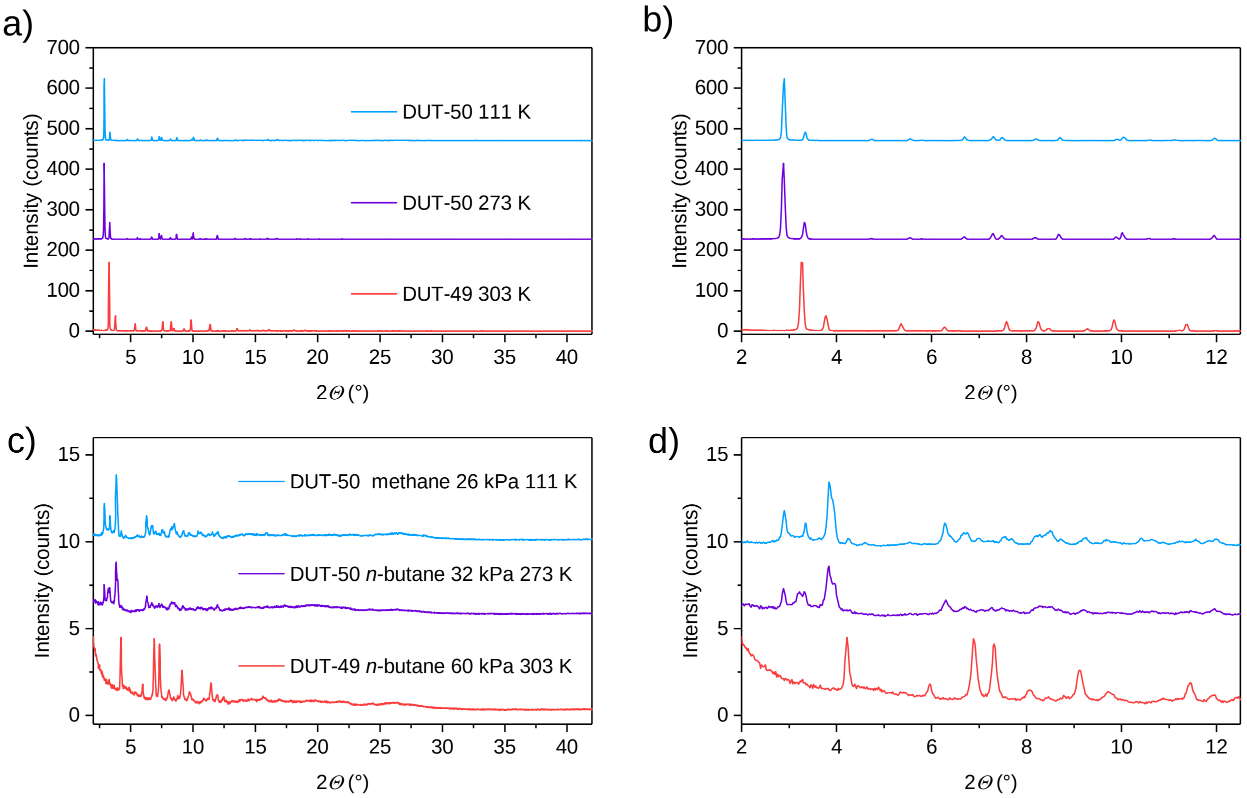

Supplementary Figure 21. Comparison of PXRD patterns of DUT-49 and DUT-50 in a,b) open pore, and c,d) in contracted pore (offset is 220 counts in a,b and 5 counts in c,d).

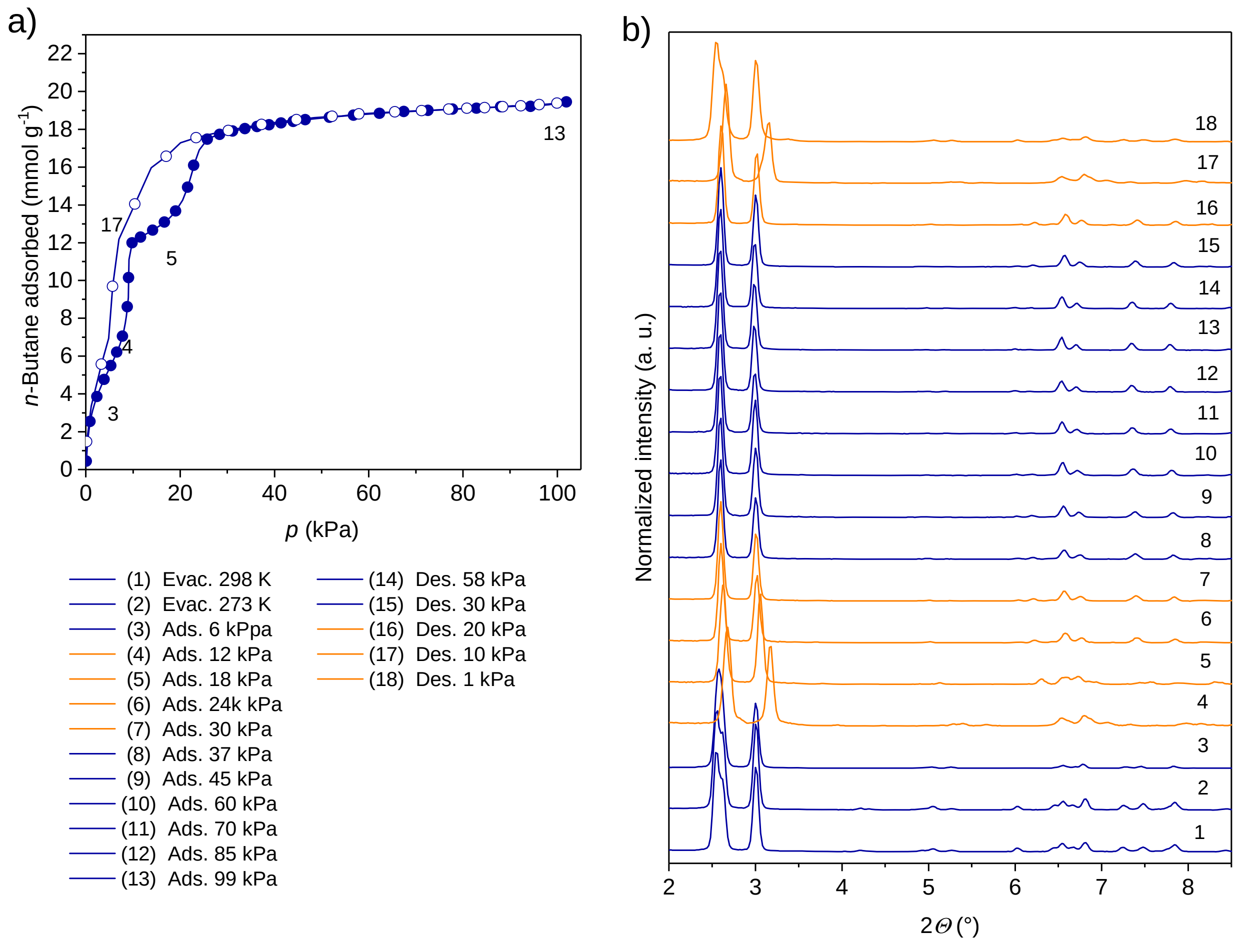

Supplementary Figure 22. DUT-151*int in situ*-PXRD dataset with a) *n*-butane at 273 K and corresponding PXRD patterns at varying pressures. Filled symbols: adsorption, empty symbols: desorption.

In situ PXRD patterns of DUT-50 show a peak broadening and reduced crystallinity upon adsorption of *n*-butane at 20 kPa and 273 K indicating the formation of *cp* phase. At elevated pressure the peaks of the pristine *op* phase reappear reflecting the reversible reopening of the structure and indicating a rare crystalline-disordered-crystalline transition. Upon desorption, the *op* phase undergoes contraction at 25 kPa without indications for reopening at lower pressure. The fact that DUT-50*cp* completely reopens at higher pressures suggests that the framework connectivity is preserved and is an evidence for a cooperative transformation within a single crystal.

In situ PXRD patterns, measured for DUT-50 during the adsorption of methane at 111 K show enhanced diffraction intensities and improved pattern quality (Supplementary Figure 23) and were thus subjected to detailed analysis involving indexing and full pattern decomposition using Le Bail fit[31], implemented into the FullProf software[32].

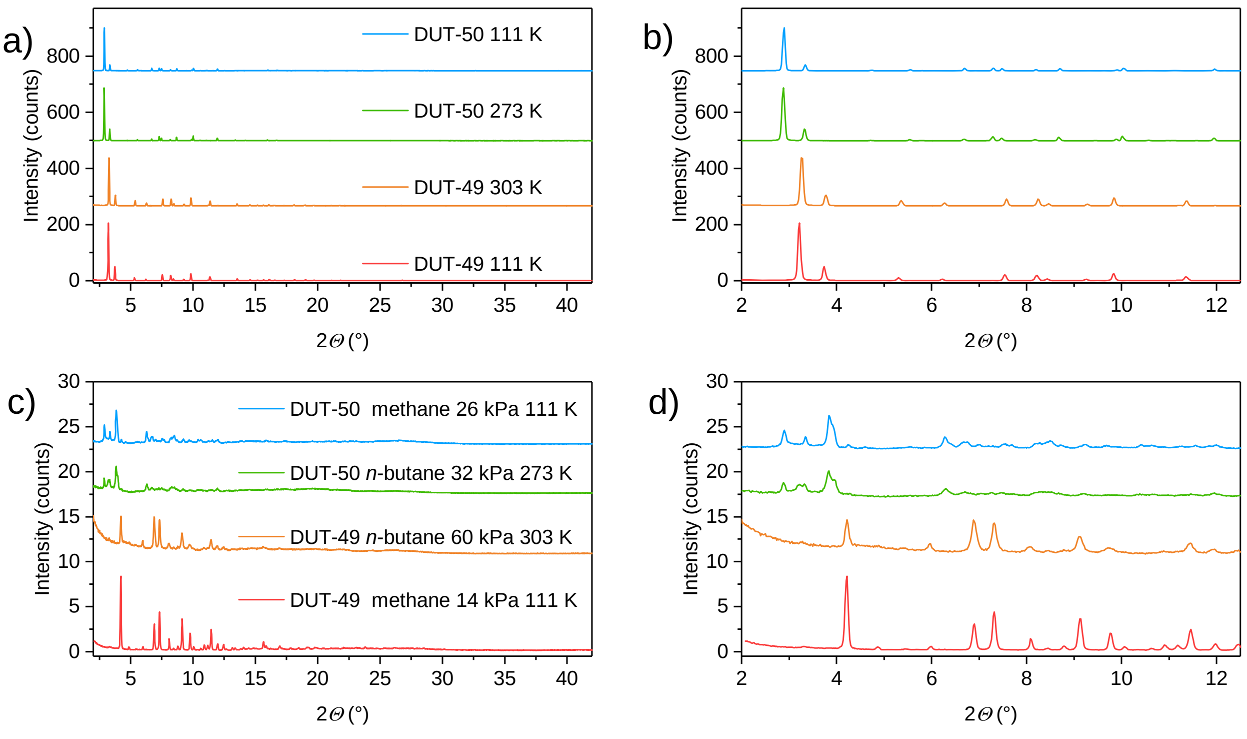

Supplementary Figure 23. Comparison of PXRD patterns of DUT-49 and DUT-50 in a,b) open pore, and c,d) in contracted pore upon adsorption of *n*-butane and methane at 111 and 303 K. a,c) wide angle diffractogram; b,d) magnification of small angle region.

The presence of a second set of reflections different from that of DUT-50*op* after contraction could be attributed to DUT-50*cp*. PXRD patterns measured during adsorption of methane between 0 and 16 kPa show phase pure DUT-50*op*. After the phase transition and NGA at 20 kPa, the PXRD patterns become more complex in comparison with DUT-49. Firstly, only part of the bulk is transformed into the *cp* phase, with peaks of the op phase present in PXRD patterns throughout the whole pressure range. The most probable reason for this behaviour are crystallite size effects which have been shown to result in phase mixtures upon adsorption of nitrogen in DUT-49[7]. Secondly, in the pressure range of 21-45 kPa during adsorption in which the *cp* phase is present, the PXRD patterns reflect low crystallinity and peak broadening at higher 2θ angles in comparison with PXRD patterns of *op* phase at lower pressures. Thirdly, the measured PXRD pattern of DUT-50*cp* contains two reflections in the 2θ range of 3.7 – 4.1° with different splitting dependent on the gas pressure in the cell. *Ab initio* indexing of PXRD patterns was difficult due to the present phase mixture and peak broadening. Therefore, an empirical analysis of the experimental PXRD patterns by using calculated PXRD patterns from simulated DUT-50*cp* phase in $Pa\bar{3}$ symmetry – the symmetry of DUT-49*cp* – and lattice parameter of 38.29 Å was performed. The reflection in the range of 3.7–4.1° of the simulated cubic unit cell with index (111) represents the interplanar distances between the space diagonals of the cubic unit cell. The splitting of the reflection can be a sign of inequality of the space diagonals in the unit cell which is only possible in non-orthogonal unit cells. Therefore, the symmetry was first reduced to rhombohedral (*R*3), however no satisfactory Le Bail fit could be obtained at larger splitting of the peak. Consequently, the symmetry of the space group was

further reduced to monoclinic $P2_1/c$ for which the multiphase Le Bail fit resulted in reasonable reliability factors. Therefore, the whole series of measured *in situ* PXRD patterns of DUT-50 upon adsorption and desorption of methane at 111 K was subjected to profile matching procedure with constant scale factor. The PXRD profile was modelled using pseudo-Voigt function with axial divergence and asymmetry of reflections was additionally treated using Berar-Baldinozzi function in Material Studio[33]. The background was refined as linear interpolation between a set of background points with reliable heights. No zero-line shift was refined during the fitting. The obtained unit cell parameters are very close to the theoretically predicted model ranging from 38.18 to 39.49 Å. The monoclinic angle, inducing the splitting of (111) and (11-1) reflections is gas pressure-dependent and ranges from 92.2° to 93.9°. The evolution of unit cell parameter is summarized in Supplementary Table 12 and displayed in Supplementary Figure 24.

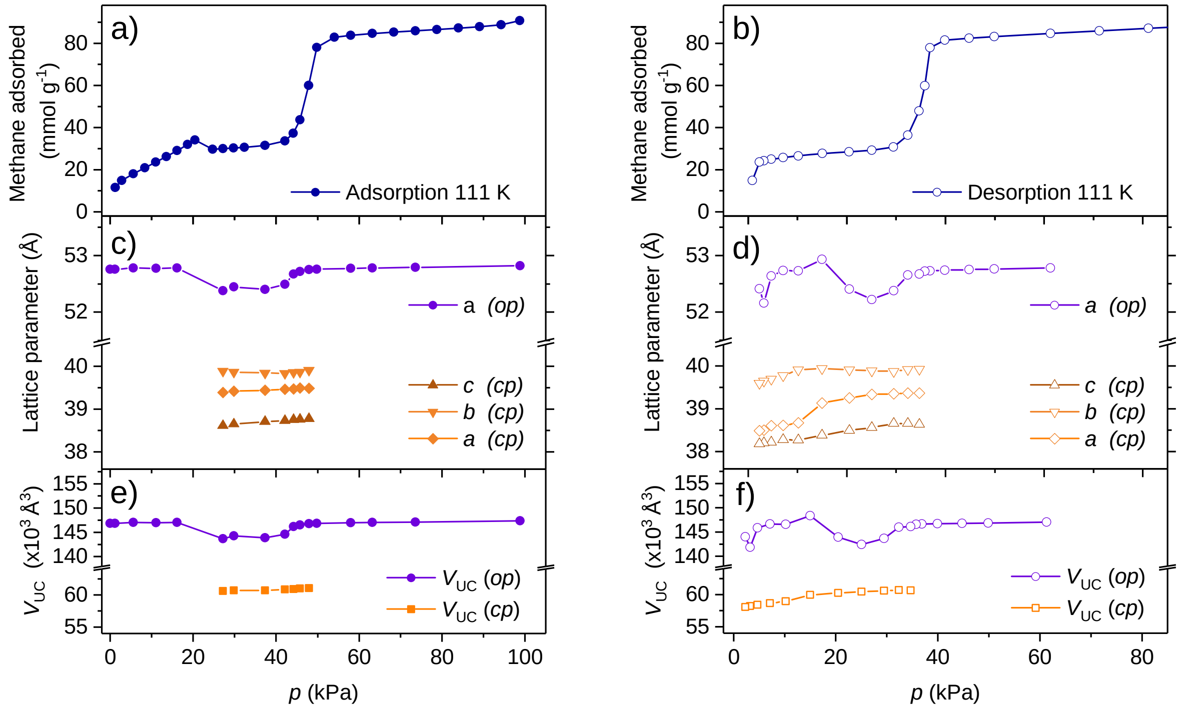

Supplementary Figure 24. a) Adsorption (filled symbols) and desorption (empty symbols) isotherm of methane at 111 K of DUT-50. c,d) Lattice parameter evolution of DUT-50 upon adsorption (c) and desorption (d) of the *op* phase (cubic symmetry $Fm\bar{3}m$) and *cp* phase (monoclinic symmetry $P2_1/c$). *op* phase is shown as purple circles, *cp* phases as orange triangles and squares.

The evolution of the unit cell volume is very similar to DUT-49 with a decrease of 58% upon structural contraction. In addition, the residual *op* phase undergoes minor contraction in the range of 25 – 45 kPa and a complete reopening of the *cp* phases can be observed at pressures beyond 50 kPa. During desorption, the *op* phase is found to transform into the *cp* phase at 38 kPa and the unit cell parameter and volume is found to decrease with decreasing pressure. Residual *op* phase is observed throughout the whole investigated pressure range. Interestingly, the unit cell volume of the op phase is slightly reduced in the range of 20-30 kPa. This indicates that the residual *op* phase also exhibits a structural response to the adsorption-induced stress upon adsorption and desorption but to a minor extent. Rietveld analysis of the experimental PXRD patterns of *cp* phase was not possible due to the poor quality and present phase

mixture. Consequently, structural models on the basis of the symmetry and lattice parameter refined by Le Bail methods were constructed and optimized with Materials Studio[33]. For this set of quasi-experimental structures the specific pore volume for the simulated structures of DUT-50*op* and *cp* was subsequently calculated using Zeo++[16] software (in detail described below). Comparison of the simulated specific pore volume of the quasi-experimental structures with the modelled structures based on MD simulation show good agreement for both *op* and *cp* phase (Supplementary Figure 25).

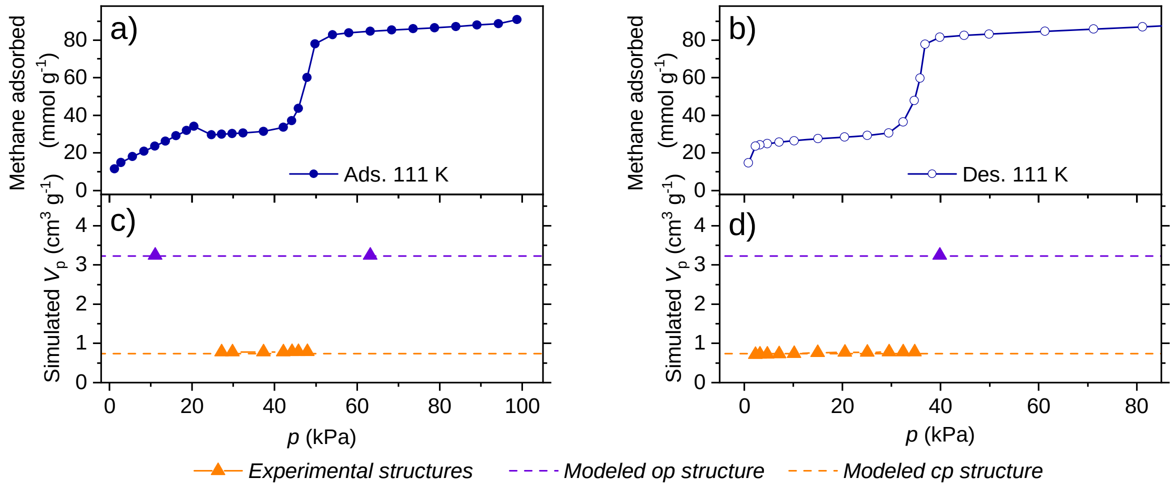

Supplementary Figure 25. a) Adsorption (filled symbols) and desorption (empty symbols) isotherm of methane at 111 K of DUT-50. c,d) Evolution of simulated pore volume (using Zeo++) of DUT-50 upon adsorption (c) and desorption (d) of the *op* phase (purple) and *cp* phase (orange). Simulated pore volumes of the structural models determined by MD simulations are given as horizontal dashed lines.

Supplementary Table 12. Data obtained from Le Bail refinement of PXRD patterns collected during adsorption of methane at 111 K in DUT-50.

P (kPa)	Pattern No (Supplementary Figure 22)	DUT-50*op* ($Fm\bar{3}m$)		DUT-50*cp* ($P2_1/c$)					*Rp* (%)	*Rwp* (%)
		a (Å)	*V* (Å^3)	*a* (Å)	*b* (Å)	*c* (Å)	*β* (°)	*V* (Å^3)		
Evac. 111K	1	52.756 (1)	146833	-	-	-	-		7.98	12.90
1.2	2	52.756 (1)	146830	-	-	-	-		7.68	12.70
5.6	3	52.777(1)	147009	-	-	-	-		7.95	12.70
11.1	4	52.775(1)	146988	-	-	-	-		8.01	13.10
16.1	5	52.779(1)	147020	-	-	-	-		8.43	13.60
27.2	6	52.380(1)	143709	38.611(3)	39.886(2)	39.384(2)	92.12(1)	60611(6)	3.60	5.56
29.8	7	52.445(4)	144252	38.651(3)	39.862 (2)	39.419 (3)	92.03 (1)	60695(6)	3.62	5.36
37.3	8	52.401(5)	143887	38.713(2)	39.841(2)	39.436(2)	92.08(1)	60684(6)	3.50	5.51
42.1	9	52.491(3)	144629	38.728(2)	39.830(2)	39.463(2)	92.08(1)	60832(6)	3.71	5.70
44.2	10	52.674(2)	146151	38.751(3)	39.854(3)	39.470(2)	92.08(1)	60915(6)	3.71	5.81
45.8	11	52.720(1)	146527	38.763(2)	39.863 (2)	39.494(2)	92.04 (1)	60987(6)	3.82	5.87
47.9	12	52.751(1)	146789	38.777 (2)	39.910(3)	39.486 (3)	92.02 (1)	61070(7)	4.05	6.60
49.8	13	52.756(1)	146829	-	-	-	-	-	6.55	10.20
58.0	14	52.773(1)	146974	-	-	-	-	-	7.01	10.80
63.2	15	52.780(1)	147031	-	-	-	-	-	6.08	9.39
73.6	16	52.792(1)	147128	-	-	-	-	-	6.86	10.20
98.8	17	52.816(1)	147335	-	-	-	-	-	7.97	11.40

Supplementary Table 13. Data obtained from Le Bail refinement of PXRD patterns collected during desorption of methane at 111 K in DUT-50.

p (kPa)	Pattern No (Supplementary Figure 22)	DUT-50*op* ($Fm\bar{3}m$)		DUT-50*cp* ($P2_1/c$)					*Rp* (%)	*Rwp* (%)
		Lattice parameter *a*, Å	*V* (Å^3)	*a* (Å)	*b* (Å)	*c* (Å)	*β* (°)	*V* (Å^3)		
61.3	18	52.779 (1)	147026	-		-	-	-	7.31	10.3
49.9	19	52.759 (1)	146857	-	-	-	-	-	6.57	9.56
44.8	20	52.749 (1)	146773	-	-	-	-	-	6.35	8.91
39.9	21	52.738(1)	146677	-	-	-	-	-	6.41	9.15
36.9	22	52.729 (1)	146608	-	-	-	-	-	6.65	9.31
35.8	23	52.723 (1)	146555	-	-	-	-	-	6.92	10.6
34.7	24	52.671 (1)	146125	38.633(2)	39.923(2)	39.368(2)	92.18 (1)	60673(3)	4.61	7.77
32.4	25	52.652(1)	145963	38.655(2)	39.918 (2)	39.365(2)	92.18(1)	60697(4)	3.90	6.42
29.5	26	52.377 (4)	143690	38.656 (3)	39.876(3)	39.354(2)	92.18 (1)	60618(6)	4.18	6.78
25.1	27	52.221(8)	142405	38.561(3)	39.887(3)	39.338 (2)	92.23 (1)	60459(7)	4.51	7.78
20.5	28	52.408(6)	143940	38.494(2)	39.907(2)	39.255(2)	92.31 (1)	60252(6)	4.12	7.05
15.0	29	52.933 (6)	148313	38.380 (2)	39.943(2)	39.138 (2)	92.41 (1)	59946(5)	3.98	6.52
10.2	30	52.727 (3)	146590	38.274(1)	39.909(1)	38.671(2)	93.21 (1)	58975(4)	3.32	4.95
7.1	31	52.735(3)	146652	38.281(1)	39.780(1)	38.614(2)	93.57(1)	58687(4)	3.25	4.70
4.7	32	52.637 (3)	145839	38.219 (1)	39.692(1)	38.604(2)	93.68 (1)	58440(4)	3.46	5.18
3.2	33	52.156(5)	141876	38.201 (2)	39.653 (2)	38.504 (2)	93.76(1)	58201(5)	3.59	5.16
2.3	34	52.413 (3)	143988	38.180(1)	39.601(1)	38.487(2)	93.78(1)	58062(4)	3.42	4.93

The PXRD patterns obtained for DUT-151*int* after supercritical activation (DUT-151*int-act*) show a shift of peaks in the range of 2-3.5 ° to lower 2Θ values and appearance of new reflections in comparison to the as made material are observed.

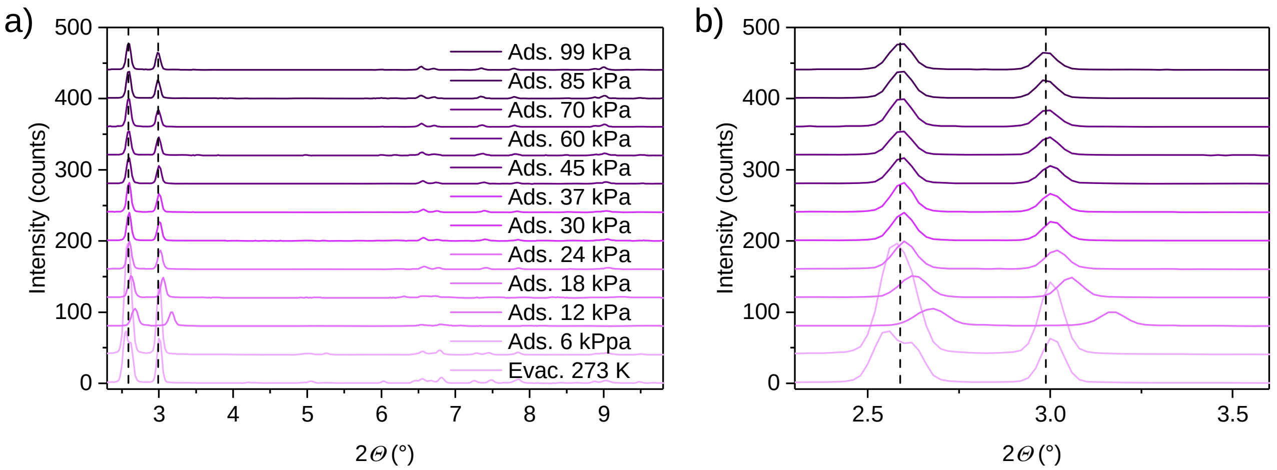

Supplementary Figure 26. a) PXRD patterns of DUT-151*int* upon loading with *n*-butane at 273 K and b) magnified small angle region. Dashed lines represent peak positions of DUT-151*int*.

This change can be assigned to a change in symmetry and unit cell volume upon solvent removal from monoclinic *C*2/*m* (in solvated DUT-151*int*) to triclinic $P\bar{1}$ in DUT-151*int-act* determined by indexing of the PXRD pattern. At intermediate pressures in the range of 8 - 10 kPa, a step and hysteresis in the isotherm are observed. Indexing of *in situ* PXRD patterns in this pressure region shows that the symmetry of the unit cell is transformed to the initial monoclinic *C*2/*m* space group, however the structure of DUT-151*int-cp* (87419 Å^3) is found to be contracted by 14% in unit cell volume in comparison to DUT-151*int* (101103 Å^3). The full list of cell parameter refined from the *in situ* PXRD data is displayed in Supplementary Table 14 and further illustrated in Supplementary Figure 27.

Supplementary Table 14. Le Bail fit of PXRD patterns (Supplementary Figure 22), measured *in situ* at 273 K during loading of *n*-butane.

p (kPa)	Space group	a (Å)	b (Å)	c (Å)	α (°)	β (°)	γ (°)	R_p	R_{wp}
Evac 298K	$P\bar{1}$	41.204(2)	40.981(2)	42.234(4)	119.62(1)	119.66(1)	59.66(6)	2.97	4.99
Evac 273	$P\bar{1}$	41.187(2)	41.013(3)	42.221(3)	119.67(1)	119.70(1)	59.64(1)	3.05	4.48
6 ads	$P\bar{1}$	41.313(3)	41.101(3)	42.183(2)	119.92(1)	119.95(1)	59.50(1)	4.44	9.54
12 ads	$C\,2/m$	70.1749(62)	37.727(24)	41.3181(19)	90	127.02(1)	90	4.99	8.42
18 ads	$C\,2/m$	71.557(3)	38.078(3)	41.207(2)	90	126.04(1)	90	4.90	7.29
24 ads	$C\,2/m$	72.185(3)	38.455(3)	41.210(2)	90	125.79 (2)	90	5.49	10.90
30 ads	$C\,2/m$	72.224(4)	38.483(4)	41.299(3)	90	125.70 (1)	90	5.87	12.1
37 ads	$C\,2/m$	72.322(3)	38.515(3)	41.342(2)	90	125.72(1)	90	5.35	9.74
45 ads	$C\,2/m$	72.387(3)	38.518(2)	41.371(2)	90	125.73(1)	90	5.09	9.18
60 ads	$C\,2/m$	72.472(3)	38.583(3)	41.461(2)	90	125.67(1)	90	5.07	9.40
70 ads	$C\,2/m$	72.512(3)	38.605(2)	41.525(2)	90	125.60(1)	90	5.34	10.10
85 ads	$C\,2/m$	72.566(4)	38.623(3)	41.562(2)	90	125.60(1)	90	6.00	12.3
99 ads	$C\,2/m$	72.673(2)	38.628(2)	41.613(1)	90	125.70(1)	90	4.87	8.58
58 des	$C\,2/m$	72.657(2)	38.615(2)	41.686(1)	90	125.75(1)	90	4.87	8.59
30 des	$C\,2/m$	72.336(3)	38.638(3)	41.679(2)	90	125.80(1)	90	5.00	9.86
20 des	$C\,2/m$	72.203(3)	38.598(3)	41.671(2)	90	125.89(1)	90	5.07	9.38
10 des	$C\,2/m$	70.624(6)	37.866(5)	41.415(3)	90	126.55(1)	90	5.81	9.46
1 des	$P\bar{1}$	41.318(4)	41.057(4)	42.266(4)	119.72(1)	119.91 (1)	59.49(1)	5.41	9.40
Single crystal	$C\,2/m$	71.700(14)	41.570(8)	41.450(8)	90	125.08(3)	90	n. a.	n. a.

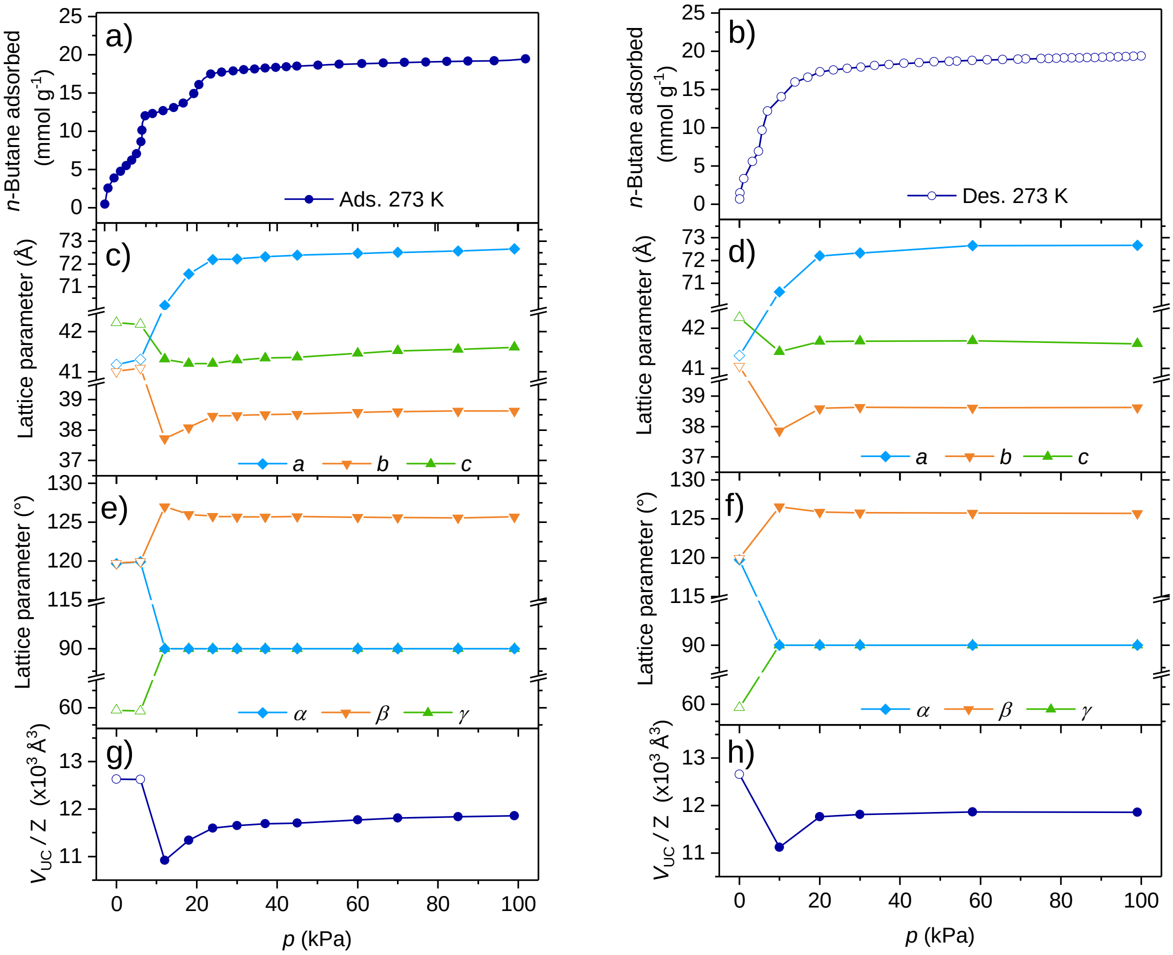

Supplementary Figure 27. a,b) Adsorption (closed symbols) and desorption (open symbols) isotherms of *n*-butane at 273 K. c-f) Corresponding lattice parameter evolution of the unit cells of DUT-151*int-act* (open symbols) and DUT-151*int-cp* and DUT-151*int-op* (closed symbols). e,f) Evolution of unit cell volume given in V_{UC} / Z to compare the volume evolution independent of the change in symmetry (Z(*int, op, cp*) = 8, Z(*act*) = 4). Tabulated values can be obtained from Supplementary Table 14.

With increasing pressure, the unit cell volume and lattice parameters increase reaching a unit cell volume of DUT-151*int-op* (101740 Å^3) in the range of the unit cell obtained from the single crystal analysis of solvated DUT-151*int*. Upon desorption of *n*-butane at 273 K and 298 K DUT-151*int-op* is found to transform back to DUT-151*int-cp* which then transforms back to the original structure of DUT-151*int-act.* In contrast to DUT-49 and DUT-50, the structural transitions in DUT-151*int* are found to be fully reversible.

The crystal structures of DUT-151*int-op*, DUT-151*int-cp*, and DUT-151*int-act* could further be refined by Rietveld methods and a procedure previously described for the refinement of DUT-50*cp* from the *in situ* PXRD patterns recorded at 60 kPa, 12 kPa and evacuated, respectively. Because of low symmetry of the structures, adsorbed *n*-butane molecules were not considered in the refinement. The Rietveld plots are displayed in Supplementary Figure 28 - Supplementary Figure 30. The main structural data are summarized in Supplementary Table 15. CCDC 1890381, 1890382, and 1890383 contain structural data for DUT-151*int-act,* DUT-151*int-cp* and DUT-151*int-op*, respectively.

Supplementary Table 15. Experimental structural data on DUT-151*int-act,* DUT-151*int-cp* and DUT-151*int-op*.

	DUT-151*int-act*	DUT-151*int-cp*	DUT-151*int-op*
Formula unit	$C_{52}H_{28}Cu_2N_2O_8$	$C_{52}H_{28}Cu_2N_2O_8$	$C_{52}H_{28}Cu_2N_2O_8$
Z	12	24	24
Symmetry, space group	triclinic, $P\bar{1}$	monoclinic, *C*2*/m*	monoclinic, *C*2*/m*
Unit cell parameters			
a (Å)	41.193(1)	69.747(19)	71.598(3)
b (Å)	41.026(1)	37.990(12)	41.628(2)
c (Å)	42.227(1)	41.139(11)	41.667(2)
α, °	119.58(2)	90	90
β, °	119.78(3)	126.68(2)	124.99(2)
γ, °	59.46(2)	90	90
Unit cell volume, (Å^3)	50493(5)	87419(40)	101740(18)
Wave length (Å)		1.5406	
2θ range (°)		2 - 30	
Instrument geometry		Debye-Scherrer	
Zero point line shift (°)	0	0	0
Profile function		Thompson-Cox-Hastings	
U	0.02797	0.81789	0.0206
V	0.00371	0.03867	0.002
W	0.00507	0.00927	0.00447
X	0.01787	0.08439	0.08557
Y	0.00027	-0.00141	0.00105
Asymmetry correction		Berar-Baldinozzi	
P1	0.00280	0.00961	-0.00021
P2	0.00010	-0.00022	0.00018
P3	-0.00359	0.00412	-0.00074
P4	-0.00011	0.00005	-0.00001
Refined motion groups	42	70	70
Refined degrees of freedom	252	388	388
		Final *R*-values	
Final R_{wp} %	14.53	12.56 %	9.42 %
Final R_p %	10.83	8.19 %	6.98 %

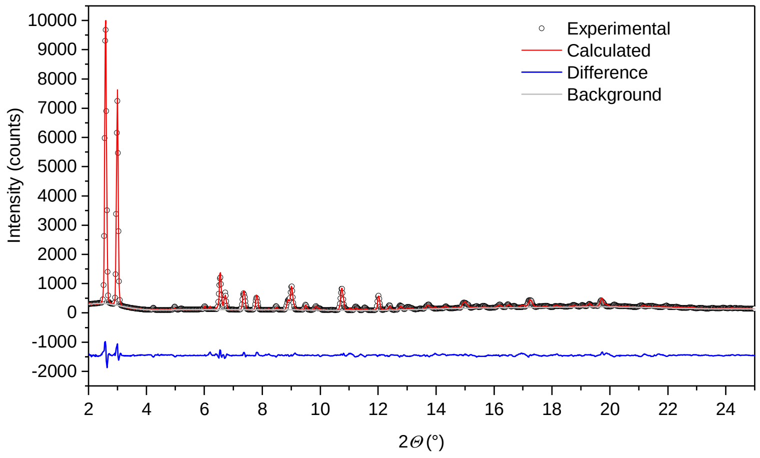

Supplementary Figure 28. Rietveld plot for DUT-151*int-act* evacuated at 273 K.

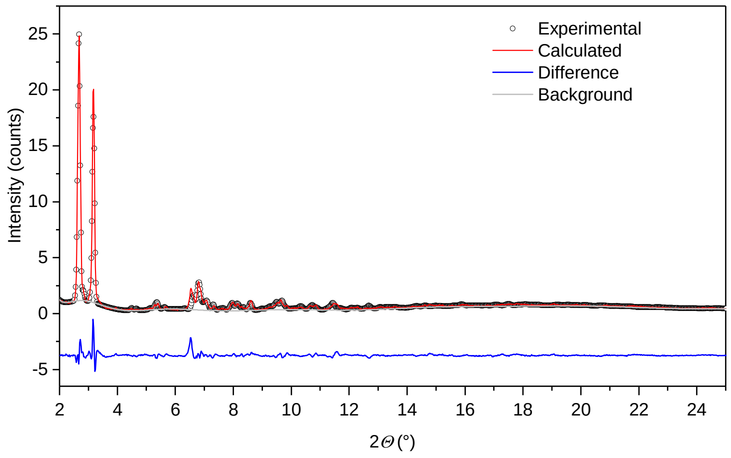

Supplementary Figure 29. Rietveld plot for DUT-151*int-cp* at 12 kPa *n*-butane adsorption at 273 K.

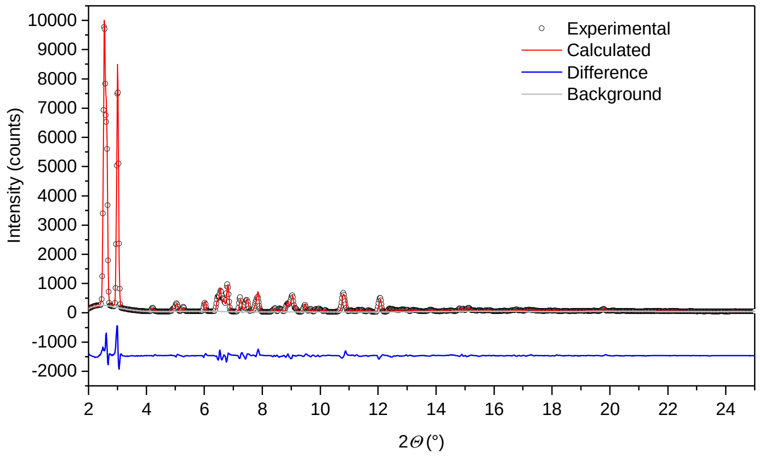

Supplementary Figure 30. Rietveld plot for DUT-151*int-op* at 60 kPa *n*-butane adsorption at 273 K.

Based on the refined structures and the single crystal structure of DUT-151*int* the structural transition in DUT-151int upon adsorption of *n*-butane at 273 K can be illustrated (Supplementary Figure 31).

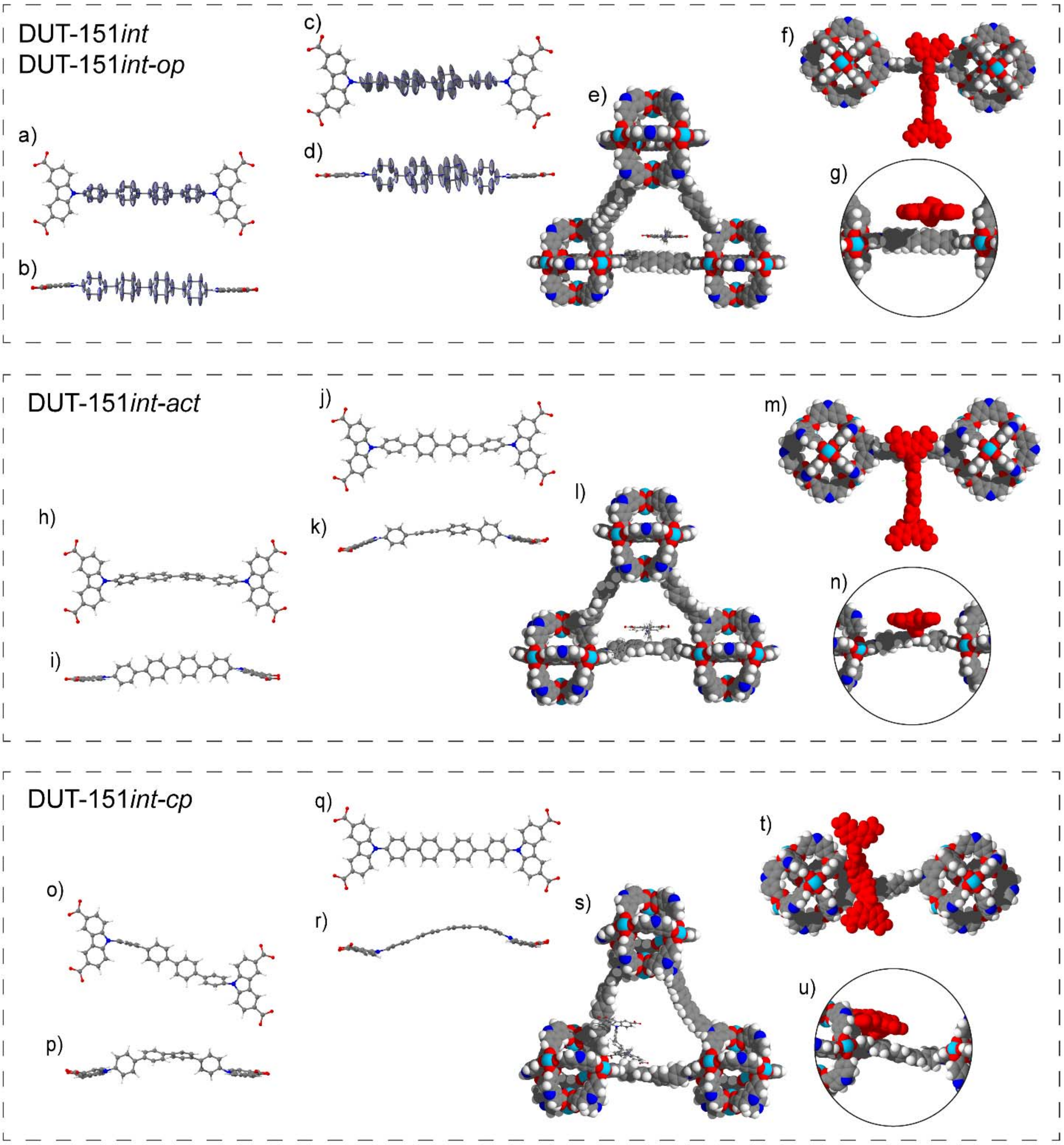

Supplementary Figure 31. a-g) single crystal structure of DUT-151*int* (which is very similar to DUT-151*int-op*), a,c) top-view and b,d) side-view of two symmetrically independent ligands including thermal ellipsoid with 50% probability of occupancy of the bridging unit, e) tetrahedral window with ligand of secondary net in red, f) top-view, g) side-view. h-n) DUT-151*int-act* h,j) top-view and i,k) side-view of two symmetrically independent ligands, l) tetrahedral window with ligand of secondary net in red, m) top-view, n) side-view. o-u) DUT-151*int-cp*, o,q) top-view and p,r) side-view of two symmetrically independent ligands, s) tetrahedral window with ligand of secondary net in red, t) top-view, u) side-view.

The symmetrically independent ligands in DUT-151*int-act* and especially in DUT-151*int-cp* are found to be deformed similar to the ligands in DUT-49*cp* although less severe. It can also be observed that the

secondary net in DUT-151*int-cp* is oriented differently, mainly due to the deformation of the ligand which results in a shrinkage of the individual nets. This crystallographic information allows to derive a structural mechanism and simulate textural properties such as pore volume and pore size distribution for the different structures in play (Supplementary Figure 74). From the PSD it is obvious that the largest pore diameter in DUT-151*int* is increased from 19 to 22 Å upon activation (DUT-151*int-act*) while maintaining a specific pore volume of 1.09 $cm^3 g^{-1}$. The increase in pore diameter can be explained by interframework dynamics in which the interframework distance is reduced due to dispersive interactions. In contrast, the difference in pore volume between DUT-151*int-op* and *-cp* is 0.32 $cm^3 g^{-1}$, less than 20 % compared with DUT-49 and 50 and in the range of the simulated transitions of DUT-48. Thus, the absence of NGA can be explained by the lack of difference in adsorption energetics and large activation barriers previously derived for DUT-48. The crystallographic analysis can also be set in context to the free energy profiles simulated for DUT-151*int* (Figure 2). A flat slope in the region of 175,000 $Å^3$ indicates the presence of slightly stabilized structures in this range of this unit cell volume. This value is in line with the experimentally observed unit cell volume of DUT-151*int-cp* that needs to be doubled to compare the experimentally obtained structures to the proposed models yielding a unit cell volume of 174,838 $Å^3$. The absence of NGA under any of the various adsorption conditions indicates that the structural contraction in DUT-151*int* lacks the metastable character well reflected by simulations of the free energy profile and absence of ligand buckling. Upon contraction, a certain degree of interframework dynamics and bending of the ligands occurs in DUT-151*int*. However, no tilting or concerted rotation of the MOPs is observed - in strong contrast to contraction transitions in DUT-49. The lower degree of ligand deformation and lower extent of reduction in pore volume are the reason for the absence of NGA, however they also enable the reversibility of the transition. Although the structural transition upon adsorption of *n*-butane in DUT-151*int* is well investigated, isotherms recorded using methane and argon show multiple steps and hysteresis that suggest a more complex structural transition compared to the observations made upon adsorption of *n*-butane. The observed structural flexibility in DUT-151*int* presents a unique example of both ligand deformation and interframework dynamics. In conclusion interpenetration is suggested to suppress contraction and NGA for this topology, however, this remains untested experimentally for a non-interpenetrated DUT-151 material.

Supplementary Note 8: Parallelised neutron powder diffraction (NPD) and methane(CD_4)-adsorption studies of microcrystalline MOF powders

In situ NPD experiments were conducted at neutron powder diffractometer E9-FIREPOD at the BER II neutron source of the Helmholtz Zentrum Berlin[34] using incoming neutrons with wavelength $\lambda = 2.8172(2)$ Å. A cylindrically-shaped aluminium sample holder with inner diameter of 1 cm and length of 12 cm was filled and sealed under argon atmosphere with 130, 115, and 95 mg of DUT-48, -49, -50, respectively. The sample holder was connected to a gas handling system via a stainless-steel capillary that allowed controlled dosing of methane in the pressure range of 0.001 – 200 kPa with a home-made apparatus. The sample was cooled by a closed cycle helium cryostat system and the temperature was monitored directly at the sample cell with a Lakeshore temperature controller with accuracy of 0.1 K. Prior to the NPD measurement the samples were outgassed in dynamic vacuum (< 0.1 Pa) at 298 K for at least 1 h. Afterwards the samples were cooled to 111 K and diffractograms of the guest-free evacuated MOFs were recorded. Based on the CH_4 isotherms at 111 K, pressures were selected to achieve different loadings of CD_4. Selected loadings/pressures are displayed in Supplementary Figure 32.

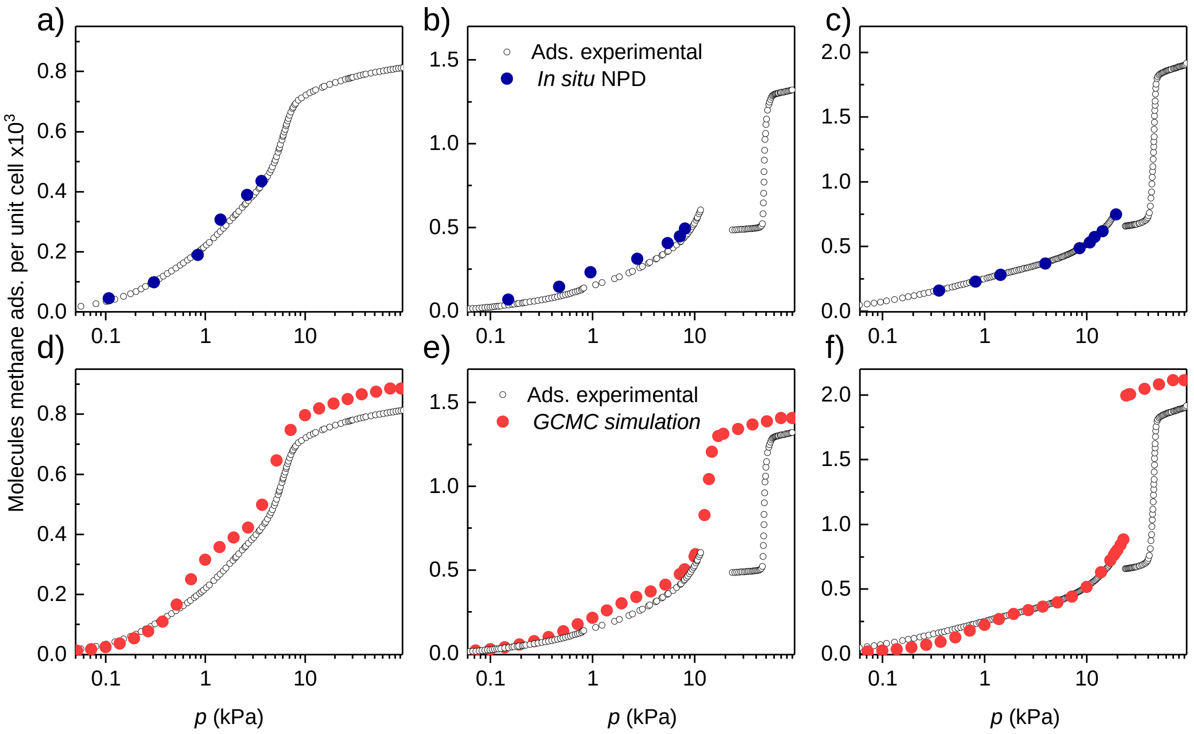

Supplementary Figure 32. Experimental volumetric methane adsorption isotherms at 111 K (open symbols) in comparison to a-c) loadings investigated by NPD (blue) and d-f) GCMC simulations (red) of a,d) DUT-48, b,e) DUT-49 c,f) DUT-50.

Due to the high porosity and large sample amount CD_4 was dosed stepwise to reach the desired loadings without the need of removing CD_4 via evacuation in the case of overdosing. The adsorbed amount of the sample was monitored in parallel by the gas dosing system and showed good agreement with the *ex situ* CH_4 isotherms. Higher loadings required multiple dosing steps and an accurate determination of the

loading by the connected gas dosing system was not possible. The loadings of CD_4 were thus determined on the basis of the *ex situ* isotherms and loadings monitored during the *in situ* experiment. Before recording NPD patterns of CD_4-loaded MOFs, the samples were equilibrated for at least 20 min until the change in pressure was below 1% over 5 min. NPD patterns were recorded in 4-9 scans with 1 - 2.5 h per scan. The detector signals were integrated and calibrated by the in-house Bean software. The final patterns of each loading were obtained by adding the individual scans to obtain good statistics. The obtained patterns for each scan were compared and only summarized to a single pattern if they were identical. For each final pattern, the measuring time and number of scans was selected until the intensity of the main reflection showed at least 10000 counts. Patterns of the evacuated samples with poor scattering were only measured up to 5000 counts. The Al-sample holder shows two reflections at 75 ° and 89 ° which were used as an internal standard to normalize the patterns and monitor the increase of intensity of the main reflections in the range of 4 - 20°. These two peaks of the sample holder were omitted from the Rietveld refinement but are shown for clarity in the difference Rietveld plots.

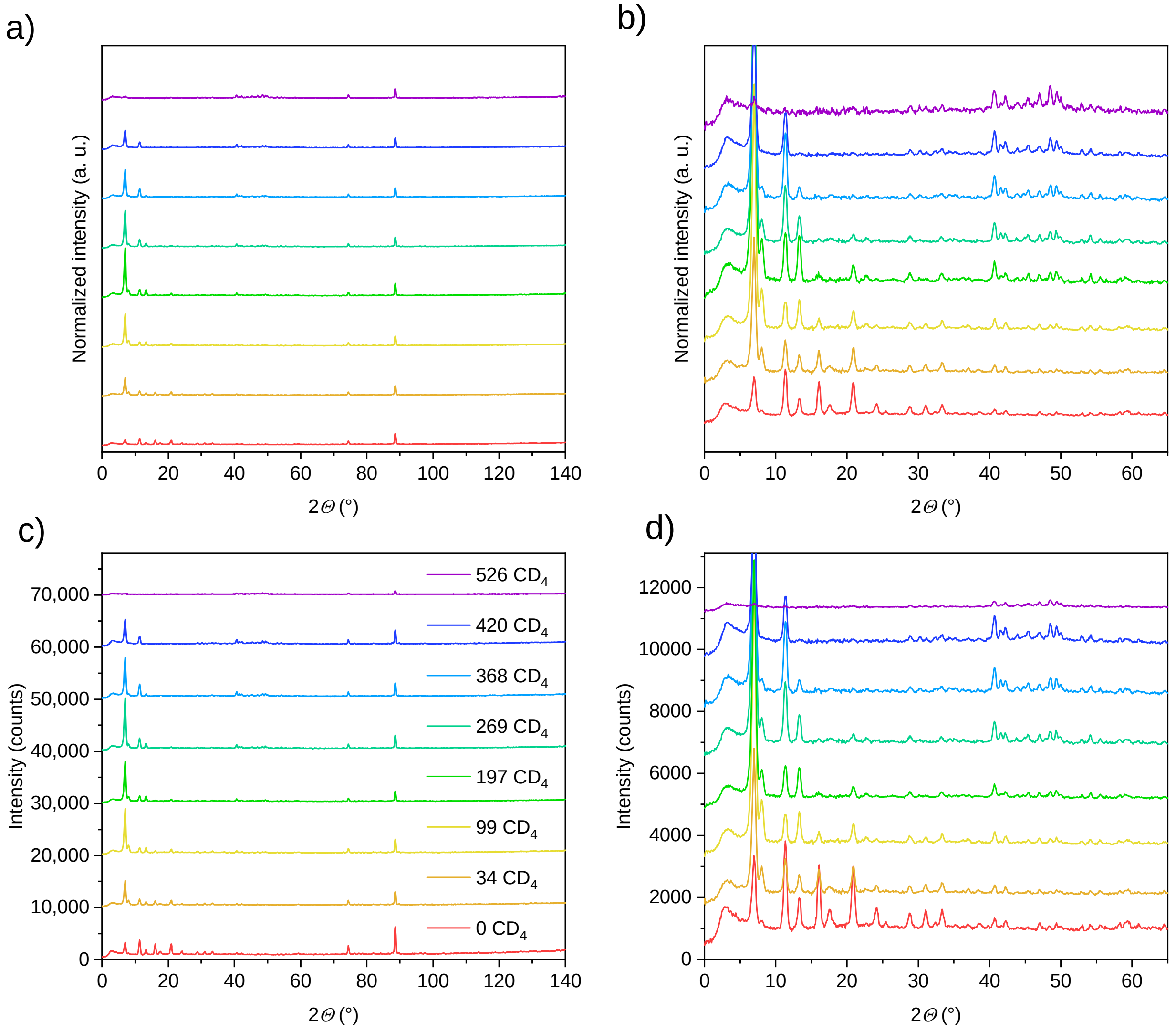

Supplementary Figure 33. NPD patterns for varying loadings with CD_4 in DUT-48: a) normalized NPD patterns with b) magnification of small angle area. c) NPD in absolute scale, and d) magnification of small angle area.

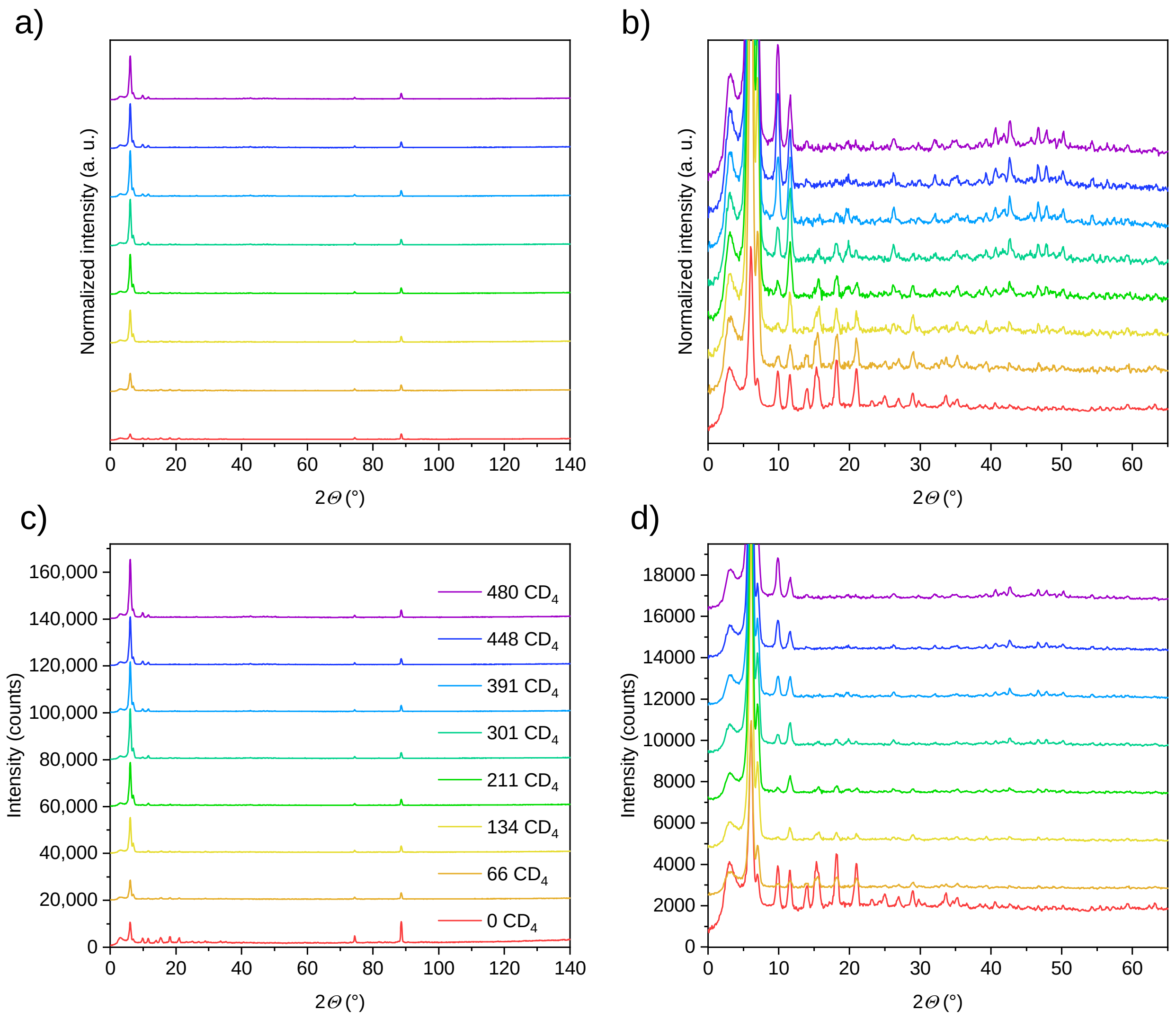

Supplementary Figure 34. NPD patterns for varying loadings with CD_4 in DUT-49: a) normalized NPD patterns with b) magnification of small angle area. c) NPD in absolute scale, and d) magnification of small angle area.

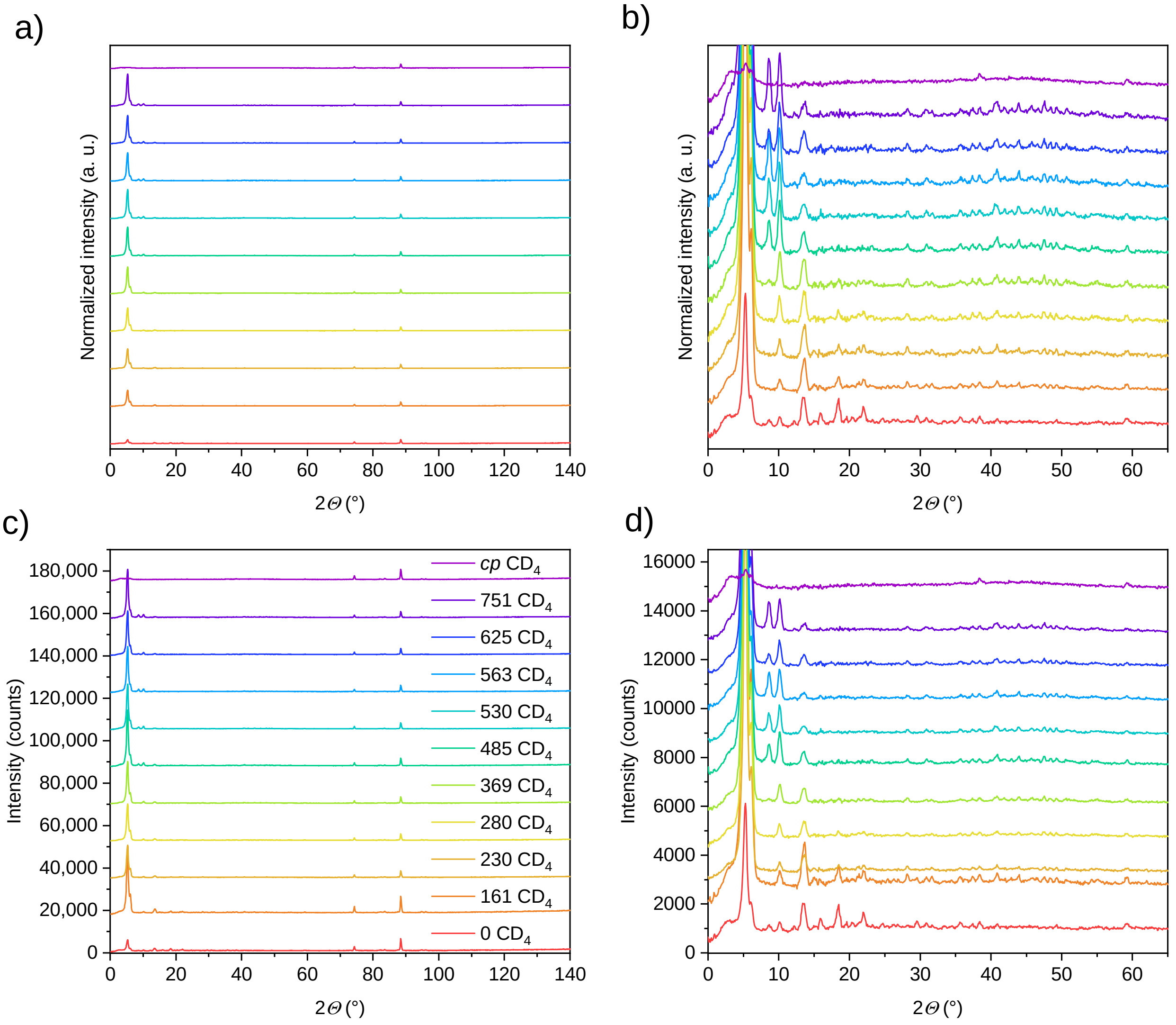

Supplementary Figure 35. NPD patterns for varying loadings with CD_4 in DUT-50: a) normalized NPD patterns with b) magnification of small angle area. c) NPD in absolute scale, and d) magnification of small angle area.

The obtained patterns for DUT-48, 49 and 50 are summarized in Supplementary Figure 33 and were analyzed by Rietveld refinement using FullProf[35]. Positions and distribution of CD_4 molecules within the unit cell of DUT-48, -49, and -50 were identified using Rietveld refinement in analogy to a recent study on CD_4 adsorption in HKUST-1[36]. The guest free single crystal structures of DUT-48, -49, and -50 described in Supplementary Table 4 were used as a structural template of the refinement. The oxygen atoms which belong to solvent or water molecules coordinated to the copper paddle-wheels were removed as they are not present in the fully activated MOF. The pseudo-Voigt function with axial divergence (implemented function 12 of FullProf) was used for profile description. Lattice parameters are refined with fixed atom positions of the framework. CD_4 molecules are treated as rigid groups with tetrahedral shape with C-D bond length of 0.999 Å[36]. For the refinement of CD_4 positions, the experimentally obtained amount of adsorbed CD_4 was introduced at random positions in the unit cell. Within the refinement, the positions of the CD_4 molecules were refined first. In a second step restriction of translation and rotation of CD_4 were applied to the methane molecules with fixed fractional occupancy of the refined positions. After validating these refinements of the positions further refinement of the fractional occupancy was performed with a fixed scale factor.[36] Occupancies of C and D atoms in the CD_4 molecules were assumed to be identical. For each obtained NPD pattern, several refinements were performed starting with slight variations of loading and positions of CD_4. The final refinements of fractional coordinates and occupancies for both, the host lattice and adsorbed CD_4 molecules including background and profile coefficients yielded good agreement with the experimental data. The Rietveld plots, reliability factors, refined amount of loading, fractional coordinates of framework and CD_4 molecules, and lattice parameters are summarized in Supplementary Figure 36 -Supplementary Figure 41, Supplementary Table 16, Supplementary Table 19 (DUT-48), Supplementary Figure 42, Supplementary Figure 48, Supplementary Table 20, Supplementary Table 23 (DUT-49), Supplementary Figure 49, Supplementary Figure 57, Supplementary Table 24, Supplementary Table 27 (DUT-50). Crystallographic files for the refined structures are accessible as Supplementary Data 2.

An intrinsic problem of the performed experiments and refinements is the decreased scattering density caused by the high mobility of methane molecules within the pores. Especially at lower loadings CD_4 is more dispersed and exhibits little interactions with other methane molecules, hence occupancies of methane at the pore walls were found below 0.4. Thus, the positions refined only reflect preferential adsorption sites but the methane molecule is not immobilized at this position of the host framework. To reduce the delocalization of methane within the pores, *in situ* NPD experiments with CD_4 adsorption in MOFs are typically performed at low temperature (77 - 4 K)[36-38] to fix the methane molecules within the pores. Ideally, this will cause immobilization of methane within the pores reflected by refinements of CD_4 positions with high occupancy up to 1 even at lower loadings. However, experiments performed on methane adsorption in ZIF-8 and MOF-5 in the range of 80-3.5 K[38] demonstrate, that a phase transition of the fluid at around 60 K occurs. At higher temperatures the adsorption process is found to be different from the adsorption sites at lower temperature. This temperature dependency of the adsorption process was further supported by computational analysis in MOF-5[39]. Consequently, experiments at lower adsorption temperature might yield better refinement of adsorption sites but do not necessarily reflect

adsorption sites and mechanism relevant for NGA at intermediate temperatures. The goal of this study was to investigate the adsorption behaviour at 111 K where NGA occurs and thus application of a lower temperature, although fundamentally interesting, would not reflect the conditions of interest for this study. Although this rather high temperature limits the localization of methane adsorption sites, the changes in diffraction intensities show that CD_4 is partly immobilized within the framework especially at higher loadings and consequently gives insights into the adsorption mechanism. Validation of these experimental results by GCMC simulations further supports the findings. Conditions of other *in situ* crystallographic experiments performed in parallel to adsorption of gases on various MOF materials are summarized in an article by Brammer and co-workers[40] and help to set the experiments described in this study in context.

For each refinement of DUT-48, the Rietveld plots are summarized in Supplementary Figure 36 - Supplementary Figure 41, and R-values, refined amount of loading, fractional coordinates of framework and CD_4 molecules, and lattice parameters are summarized in the following.

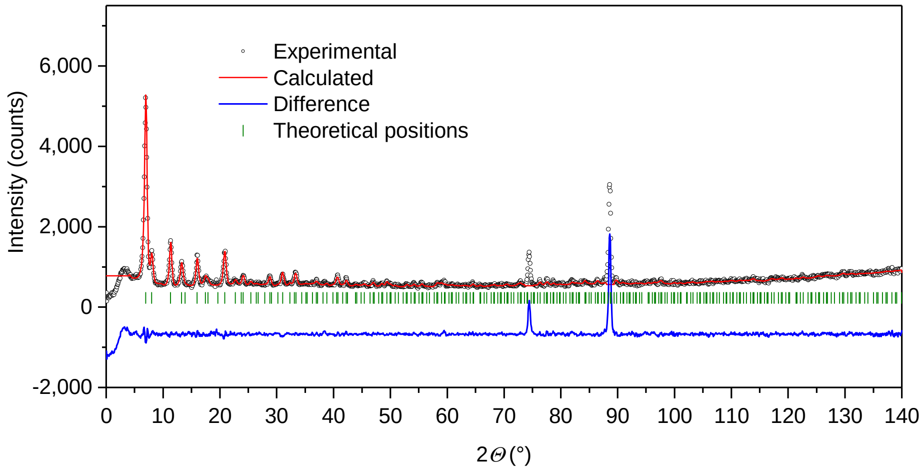

Supplementary Figure 36. Rietveld plot of DUT-48 with a loading of 45 CD_4 molecules per unit cell at 111 K.

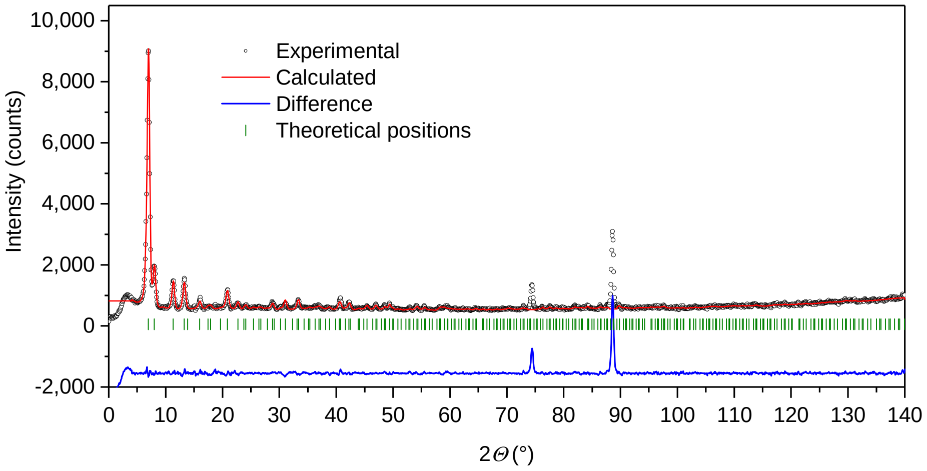

Supplementary Figure 37. Rietveld plot of DUT-48 with a loading of 99 CD_4 molecules per unit cell at 111 K.

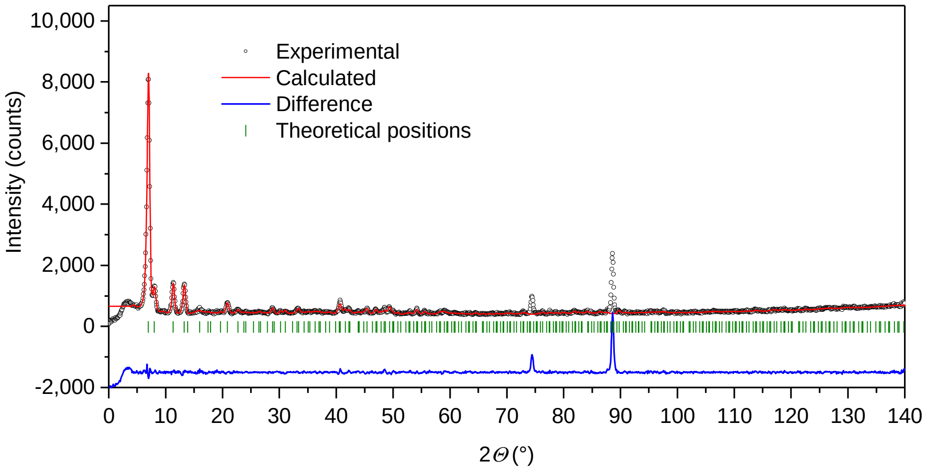

Supplementary Figure 38. Rietveld plot of DUT-48 with a loading of 189 CD_4 molecules per unit cell at 111 K.

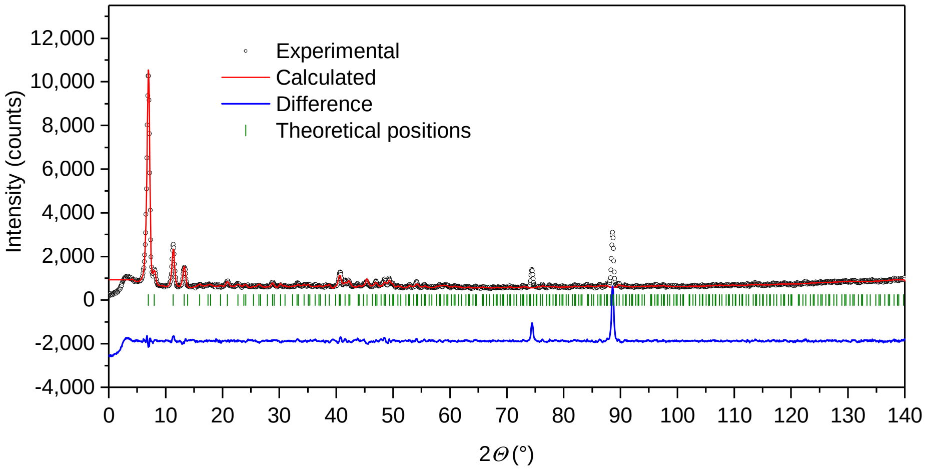

Supplementary Figure 39. Rietveld plot of DUT-48 with a loading of 306 CD_4 molecules per unit cell at 111 K.

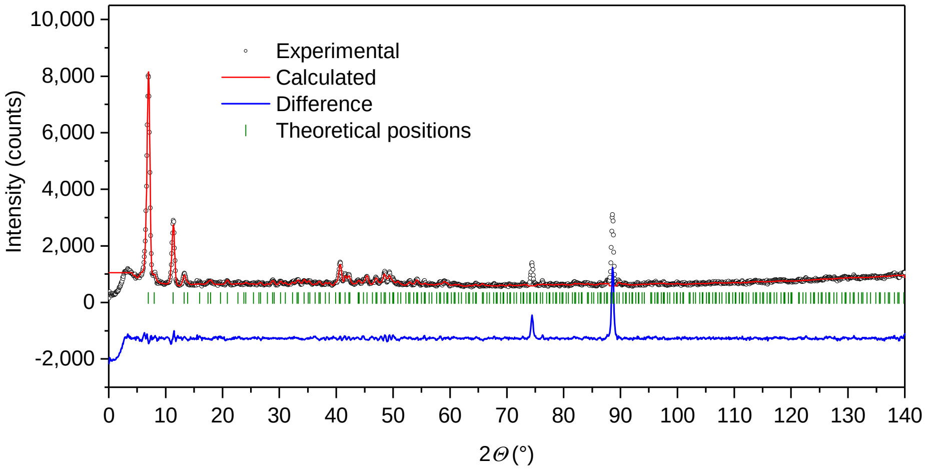

Supplementary Figure 40. Rietveld plot of DUT-48 with a loading of 389 CD_4 molecules per unit cell at 111 K.

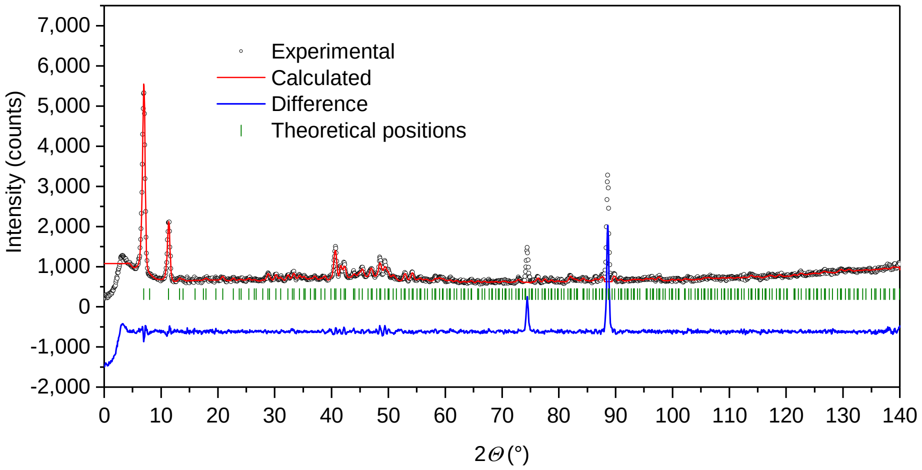

Supplementary Figure 41. Rietveld plot of DUT-48 with a loading of 435 CD_4 molecules per unit cell at 111 K.

Supplementary Table 16. Crystallographic information for DUT-48.

Material ID	DUT-48
Chemical formula, weight	$Cu_2O_8C_{34}H_{16}N_2$, 707.6 g mol^{-1}
Empirical formula, weight	$Cu_{48}O_{192}C_{960}H_{480}N_{48}$, 16982.4 g mol^{-1}
Temperature	111 K
Wavelength	Neutrons; 2.8172(2) Å
Measurement system	E9-FIREPOD at the BER II neutron source, Helmholtz Zentrum Berlin
Crystal system	Cubic
Space group	$Fm\bar{3}m$ (No. 225)
Unit cell dimensions	40.4365(15) Å
Volume	66118(4) Å^3
Z	24
Density	0.426 g cm^{-3}
2Θ range for data collection	-9.40873 ° – 141.79128 °
2Θ resolution	0.075 °
No. of data points	2017
Refinement method, program	Rietveld L.S. refinement, Full Prof

Supplementary Table 17. Lattice and profile parameters for Rietveld refinements of CD_4 loaded DUT-48.

Suppl. Figure	a (Å)	R_p	R_{wp}	R_{exp}	χ^2	R-Bragg	CD_4 per UC exp.	CD_4 per UC refined
Supplementary Figure 36	40.435(2)	3.29	4.20	3.80	1.22	4.88	34	45
Supplementary Figure 37	40.436(3)	3.32	4.35	3.72	1.37	4.17	99	99
Supplementary Figure 38	40.446(2)	3.40	4.38	4.20	1.09	4.19	197	189
Supplementary Figure 39	40.453(3)	3.68	4.80	3.59	1.79	4.20	269	306
Supplementary Figure 40	40.450(2)	3.57	4.65	3.55	1.71	4.77	368	389
Supplementary Figure 41	40.4365(16)	3.03	3.81	3.52	1.18	6.97	420	435
No refinement possible due to low diffraction intensities							526	n.a.

Supplementary Table 18. Fractional coordinates of asymmetric unit in guest free DUT-48.

		Coordinates				
Atom-ID	Atom	*x*	*y*	*z*	U_{iso}	Occupancy
CuA	Cu	0.00000	0.00000	0.17184	0.01299	1.00000
CuB	Cu	0.00000	0.00000	0.23683	0.01299	1.00000
OA	O	0.00000	0.04849	0.17676	0.01316	1.00000
OB	O	0.00000	0.04828	0.23210	0.01316	1.00000
NA	N	0.00000	0.20092	0.20092	0.01316	1.00000
CA	C	0.00000	0.06252	0.20440	0.01408	1.00000
CB	C	0.00000	0.09904	0.20453	0.01408	1.00000
CC	C	0.00000	0.11628	0.23485	0.01408	1.00000
HCA	H	0.00000	0.10430	0.25440	0.04711	1.00000
CD	C	0.00000	0.15016	0.23632	0.01408	1.00000
HDA	H	0.00000	0.16120	0.25640	0.04711	1.00000
CE	C	0.00000	0.16728	0.20660	0.01408	1.00000
CF	C	0.00000	0.15079	0.17610	0.01408	1.00000
CG	C	0.00000	0.11657	0.17520	0.01408	1.00000
HGA	H	0.00000	0.10550	0.15510	0.04711	1.00000
CH	C	0.00000	0.22608	0.22608	0.01408	1.00000
CI	C	0.02412	0.24912	0.22658	0.01408	0.50000
HIA	H	0.04070	0.24870	0.21070	0.04713	0.50000
HIA	H	0.04070	0.24870	0.21070	0.04713	0.50000

Supplementary Table 19. Fractional coordinates of CD_4 molecules in DUT-48 at varied loadings.

CD_4 per UC	Atom-ID	Atom	*X*	*y*	*z*	U_{iso}	Occupancy
45	AD1	C	0.00000	0.04235	0.09175	0.01408	0.1821(4)
45	BD1	C	0.09237	0.09237	0.15638	0.01408	0.2841(6)
99	AD1	C	0.00000	0.04665	0.08156	0.01408	0.255(7)
99	BD1	C	0.08911	0.08911	0.16049	0.01408	0.773(14)
189	AD1	C	0.08792	0.08792	0.15818	0.01408	0.85(2)
189	BD1	C	0.09444	0.09444	0.24697	0.01408	0.50(2)
189	CD1	C	0.07189	0.07189	0.00000	0.01408	0.644(17)
189	DD1	C	0.17883	0.17883	0.08643	0.01408	0.30(2)
306	AD1	C	0.09571	0.09571	0.16643	0.01408	1.00000
306	BD1	C	0.07523	0.07523	0.26657	0.01408	1.00000
306	CD1	C	0.07822	0.07822	0.00000	0.01408	1.00000
306	DD1	C	0.20002	0.20002	0.09155	0.01408	0.69(2)
389	AD1	C	0.09354	0.09354	0.17899	0.01408	0.721(20)
389	BD1	C	0.08081	0.08081	0.26427	0.01408	0.65(2)
389	CD1	C	0.10955	0.32467	0.21599	0.01408	0.338(13)
389	DD1	C	0.17104	0.17104	0.53480	0.01408	0.596(20)
389	ED1	C	0.19243	0.19243	0.10024	0.01408	0.91(2)
389	FD1	C	0.08635	0.08635	0.00000	0.01408	1.000(12)
435	AD1	C	0.09117	0.09117	0.16207	0.01408	0.87(3)
435	BD1	C	0.07462	0.07462	0.30334	0.01408	0.57(4)
435	CD1	C	0.21012	0.21011	0.40755	0.01408	0.57(3)
435	DD1	C	0.16780	0.16780	0.46469	0.01408	0.48(3)
435	ED1	C	0.19111	0.19111	0.10157	0.01408	0.91(3)

Recently, Rowland *et al.* presented *in situ* NPD analysis of CD_4 adsorption in PCN-81[3] previously demonstrated as isostructural to DUT-48. In their work the authors used the synthesis and activation procedure described by Zhou and co-workers[4]. The NPD experiments were performed at 7 K and the lower $Pa\overline{3}$ symmetry refined for PCN-81 was used for Rietveld refinement of the obtained NPD pattern to obtain primary adsorption sites of methane within the porous structure of PCN-81. Loadings of 48, 92, 192 CD_4 molecules per unit cell were analysed only covering the low-pressure region of the adsorption isotherm. Their report supports the findings of the present study on DUT-48 in which only open metal sites and the cub voids are filled with methane at these low loadings.

For each refinement of DUT-49, the Rietveld plots are summarized in Supplementary Figure 42-Supplementary Figure 48, and R-values, refined amount of loading, fractional coordinates of framework and CD_4 molecules, and lattice parameters are summarized in the following.

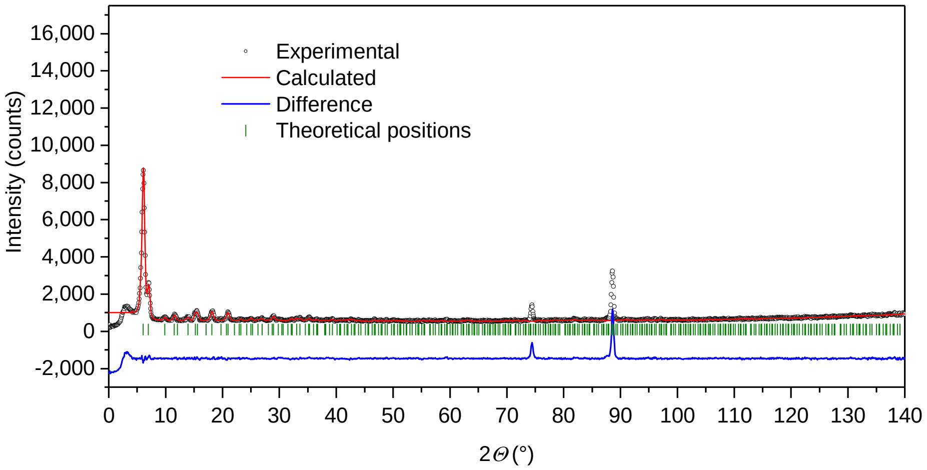

Supplementary Figure 42. Rietveld plot of DUT-49 with a loading of 68 CD_4 molecules per unit cell at 111 K.

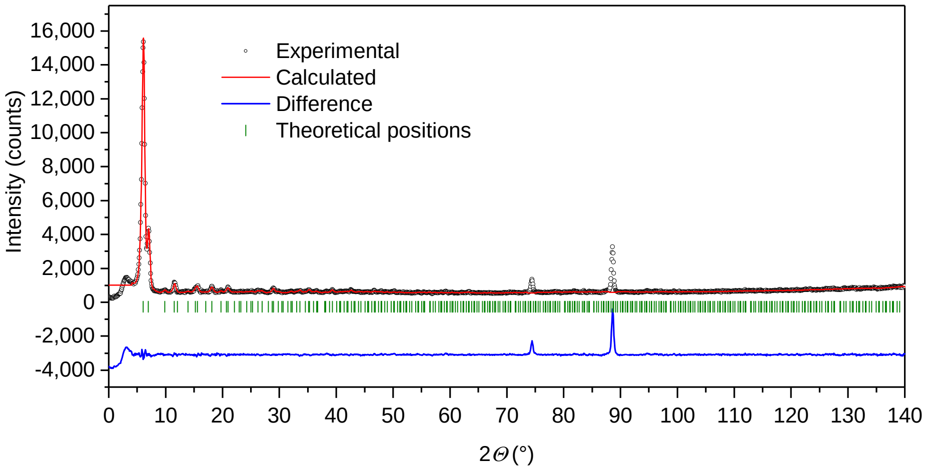

Supplementary Figure 43. Rietveld plot of DUT-49 with a loading of 145.5 CD_4 molecules per unit cell at 111 K.

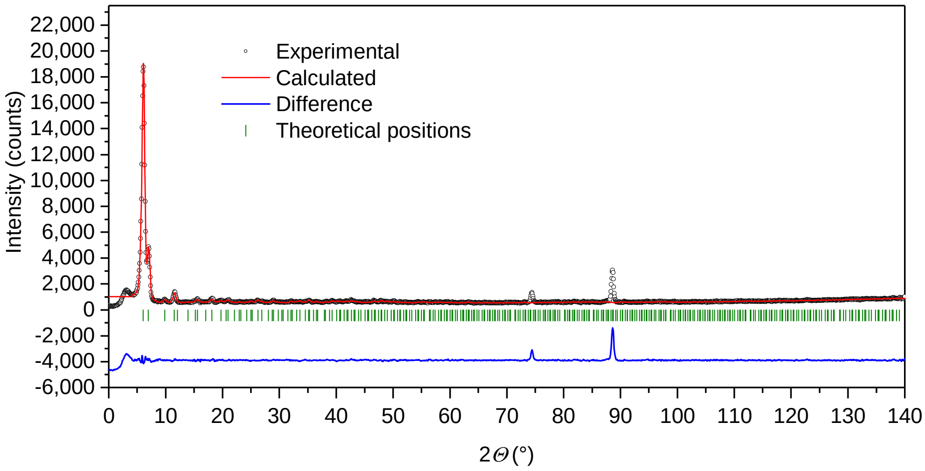

Supplementary Figure 44. Rietveld plot of DUT-49 with a loading of 231 CD_4 molecules per unit cell at 111 K.

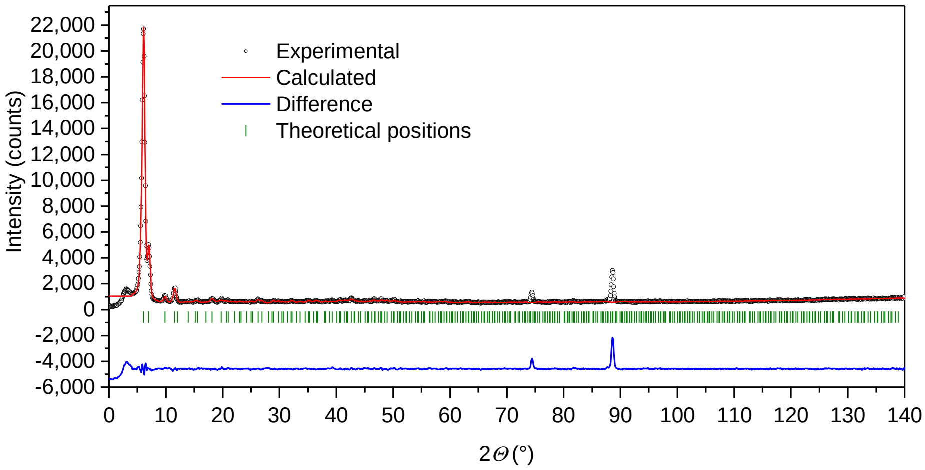

Supplementary Figure 45. Rietveld plot of DUT-49 with a loading of 311 CD_4 molecules per unit cell at 111 K.

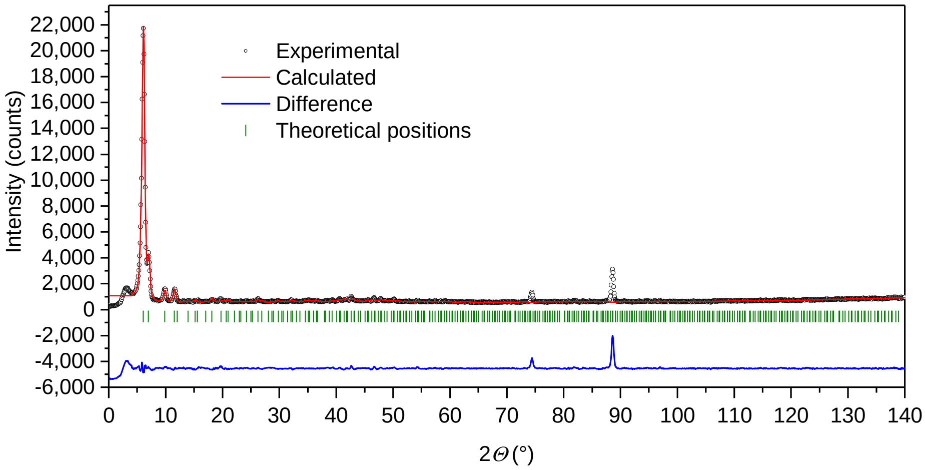

Supplementary Figure 46. Rietveld plot of DUT-49 with a loading of 405 CD_4 molecules per unit cell at 111 K.

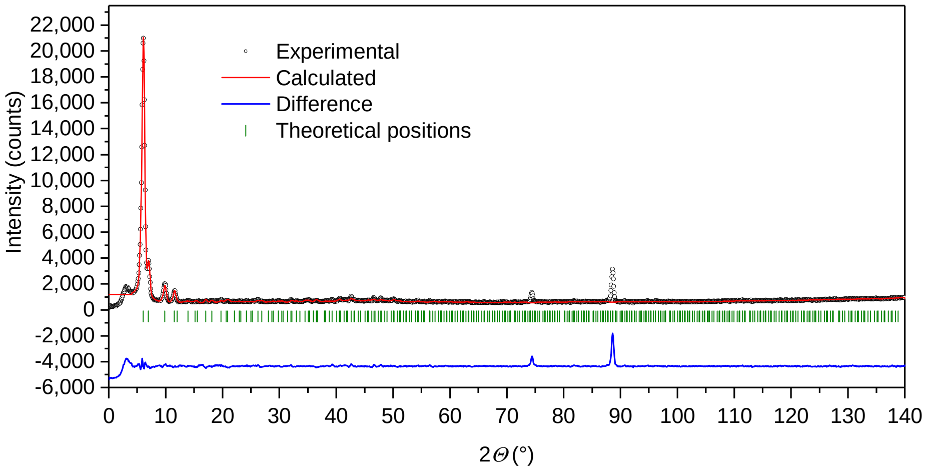

Supplementary Figure 47. Rietveld plot of DUT-49 with a loading of 446.5 CD_4 molecules per unit cell at 111 K.

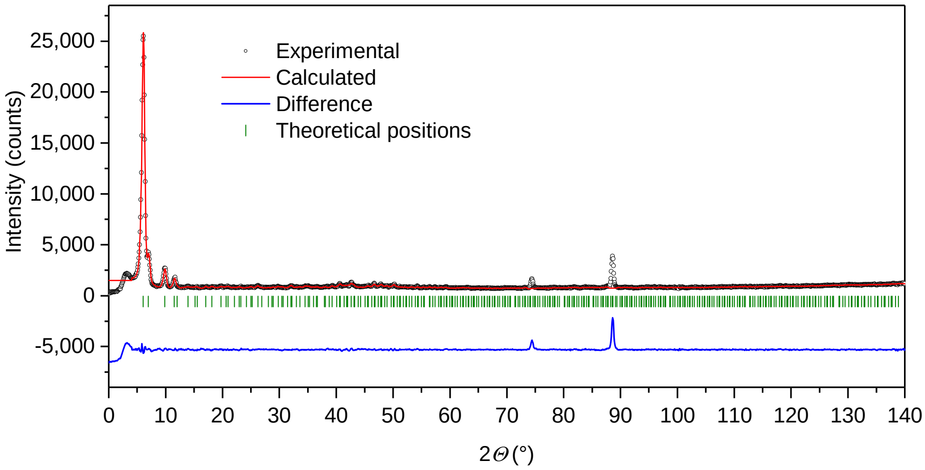

Supplementary Figure 48. Rietveld plot of DUT-49 with a loading of 492 CD_4 molecules per unit cell at 111 K.

Supplementary Table 20. Crystallographic information for DUT-49.

Material ID	DUT-49
Chemical formula, weight	$Cu_2O_8C_{40}H_{20}N_2$, 783.7 g mol^{-1}
Empirical formula, weight	$Cu_{48}O_{192}C_{960}H_{480}N_{48}$, 18808.8 g mol^{-1}
Temperature	111 K
Wavelength	Neutrons; 2.8172(2) Å
Measurement system	E9-FIREPOD at the BER II neutron source, Helmholtz Zentrum Berlin
Crystal system	Cubic
Space group	$Fm\bar{3}m$ (No. 225)
Unit cell dimensions	46.391(7) Å
Volume	99836(26) Å^3
Z	24
Density	0.312821 g cm^{-3}
2Θ range for data collection	-9.40873 ° – 141.79128 °
2Θ resolution	0.075 °
No. of data points	2017
Refinement method, program	Rietveld L.S. refinement, Full Prof

Supplementary Table 21. Lattice and profile parameters for Rietveld refinements of CD_4 loaded DUT-49.

Suppl. Figure	a (Å)	R_p	R_{wp}	R_{exp}	χ^2	R-Bragg	CD_4 per UC exp.	CD_4 per UC refin.
Supplementary Figure 42	46.425(4)	2.91	3.71	3.71	1.00	5.38	66	68
Supplementary Figure 43	46.436(4)	3.02	3.95	3.60	1.20	2.84	134	145.5
Supplementary Figure 44	46.447(5)	3.12	4.07	3.54	1.32	2.98	211	231
Supplementary Figure 45	46.473(5)	3.22	4.30	3.48	1.53	3.23	301	311
Supplementary Figure 46	46.469(4)	3.42	4.65	3.45	1.82	3.33	391	405
Supplementary Figure 47	46.477(5)	3.39	4.56	3.42	1.77	4.19	448	446.5
Supplementary Figure 48	46.472(4)	2.82	3.75	3.05	1.51	2.61	480	492

Supplementary Table 22. Fractional coordinates of asymmetric unit in guest free DUT-49.

		Coordinates				
Atom-ID	Atom	*x*	*y*	*z*	U_{iso}	Occupancy
CuA	Cu	0.00000	0.00000	0.35040	0.01299	1.00000
CuB	Cu	0.00000	0.00000	0.29560	0.01299	1.00000
OA	O	0.00000	0.04041	0.34750	0.01316	1.00000
OB	O	0.00000	0.04240	0.29930	0.01316	1.00000
CA	C	0.00000	0.05550	0.32360	0.01408	1.00000
CB	C	0.00000	0.08810	0.32440	0.01408	1.00000
CC	C	0.00000	0.10400	0.34990	0.01408	1.00000
HC	H	0.00000	0.09437	0.36818	0.04711	1.00000
CD	C	0.00000	0.13410	0.34850	0.01408	1.00000
CE	C	0.00000	0.14640	0.32100	0.01408	1.00000
CF	C	0.00000	0.13100	0.29500	0.01408	1.00000
HF	H	0.00000	0.14059	0.27663	0.04711	1.00000
CG	C	0.00000	0.10110	0.29720	0.01408	1.00000
HG	H	0.00000	0.08949	0.28016	0.04711	1.00000
NA	N	0.00000	0.17690	0.32310	0.01316	1.00000
CH	C	0.00000	0.19870	0.30130	0.01408	1.00000
CI	C	0.02410	0.21690	0.29970	0.01408	0.50000
HI	H	0.03991	0.21474	0.31282	0.04711	0.50000
CJ	C	0.02410	0.23820	0.27850	0.01408	0.50000
HJ	H	0.04005	0.25122	0.27643	0.04711	0.50000
CK	C	0.00000	0.23980	0.26020	0.01408	1.00000

Supplementary Table 23. Fractional coordinates of CD_4 molecules in DUT-49 at varied loadings.

CD_4 per UC	Atom-ID	Atom	*x*	*y*	*z*	U_{iso}	Occupancy
68	AD1	C	0.02078	0.02078	0.41049	0.01408	0.307(8)
68	BD1	C	0.08155	0.08155	0.34879	0.01408	0.399(14)
145.5	AD1	C	0.02182	0.02182	0.41981	0.01408	0.324(13)
145.5	BD1	C	0.08702	0.08702	0.43858	0.01408	0.2803(9)
145.5	CD1	C	0.07616	0.16468	0.39687	0.01408	0.386(10)
145.5	DD1	C	0.00374	0.00374	0.22505	0.01408	0.1384(5)
231	AD1	C	0.02459	0.02459	0.41011	0.01408	0.522(11)
231	BD1	C	0.11533	0.11533	0.44070	0.01408	0.686(17)
231	CD1	C	0.08399	0.18661	0.37417	0.01408	0.400(12)
231	DD1	C	0.04710	0.04710	0.23536	0.01408	0.396(16)
311	AD1	C	0.04097	0.04097	0.41978	0.01408	0.652(13)
311	BD1	C	0.12171	0.12171	0.43375	0.01408	0.81(2)
311	CD1	C	0.08444	0.19053	0.36756	0.01408	0.397(17)
311	DD1	C	0.06153	0.06153	0.27431	0.01408	0.99(3)
405	AD1	C	0.04447	0.04447	0.40690	0.01408	0.898(16)
405	BD1	C	0.13369	0.13369	0.43118	0.01408	1.000
405	CD1	C	0.06705	0.23219	0.36328	0.01408	0.661(18)
405	DD1	C	0.06492	0.06492	0.27173	0.01408	1.000
446.5	AD1	C	0.02117	0.06317	0.40297	0.01408	0.542(9)
446.5	BD1	C	0.03565	0.14019	0.25011	0.01408	0.687(17)
446.5	CD1	C	0.13325	0.07868	0.33728	0.01408	0.596(16)
446.5	DD1	C	0.07827	0.07827	0.27634	0.01408	1.000
492	AD1	C	0.01530	0.05400	0.40869	0.01408	0.386(7)
492	BD1	C	0.08305	0.08305	0.35000	0.01408	1.000
492	CD1	C	0.07386	0.07386	0.27129	0.01408	1.000
492	DD1	C	0.02622	0.19969	0.13841	0.01408	0.415(15)
492	ED1	C	0.06947	0.16414	0.28080	0.01408	0.572(17)
492	FD1	C	0.12357	0.12357	0.29867	0.01408	0.38(3)

For each refinement of DUT-50, the Rietveld plots are summarized in Supplementary Figure 49-Supplementary Figure 57, and R-values, refined amount of loading, fractional coordinates of framework and CD_4 molecules, and lattice parameters are summarized in the following.

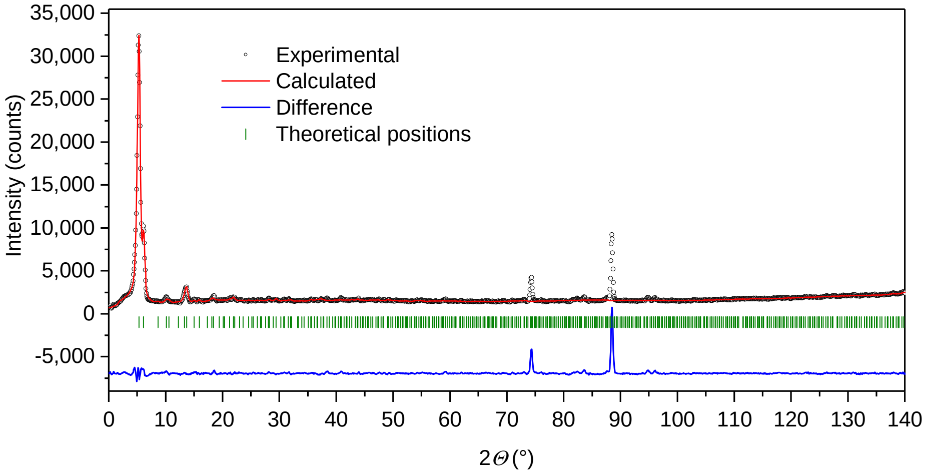

Supplementary Figure 49. Rietveld plot of DUT-50 with a loading of 159 CD_4 molecules per unit cell at 111 K.

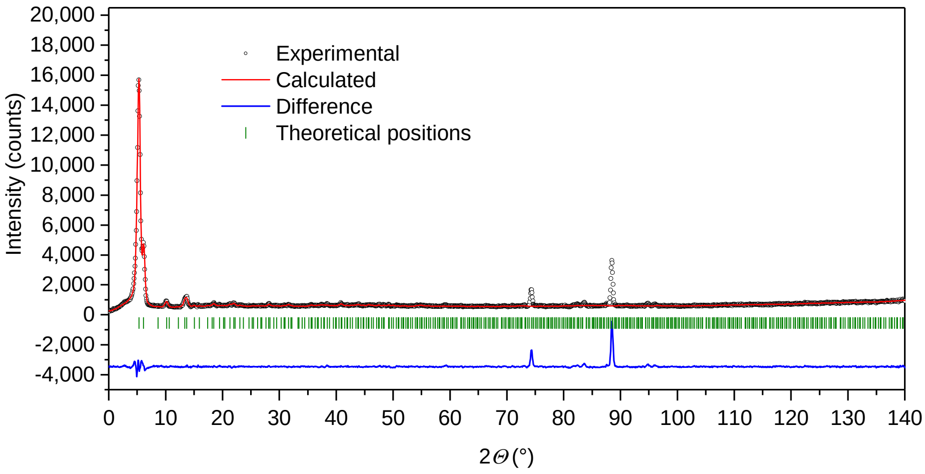

Supplementary Figure 50. Rietveld plot of DUT-50 with a loading of 228.5 CD_4 molecules per unit cell at 111 K.

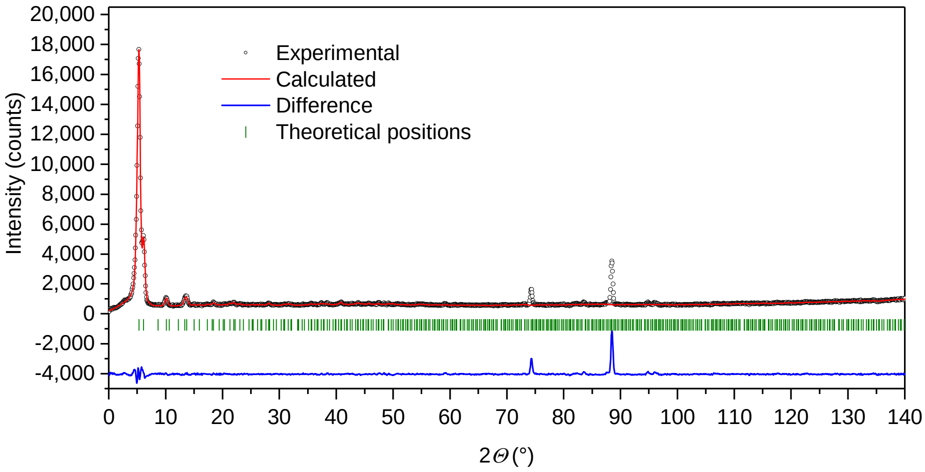

Supplementary Figure 51. Rietveld plot of DUT-50 with a loading of 280 CD_4 molecules per unit cell at 111 K.

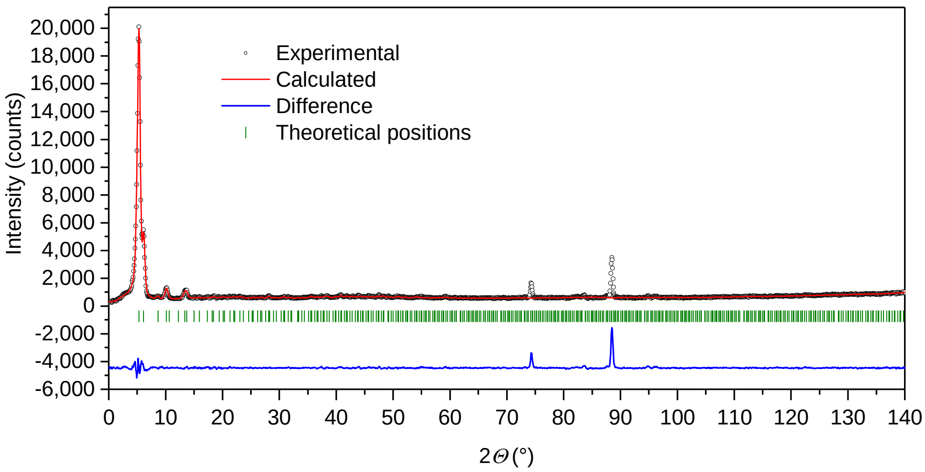

Supplementary Figure 52. Rietveld plot of DUT-50 with a loading of 370 CD_4 molecules per unit cell at 111 K.

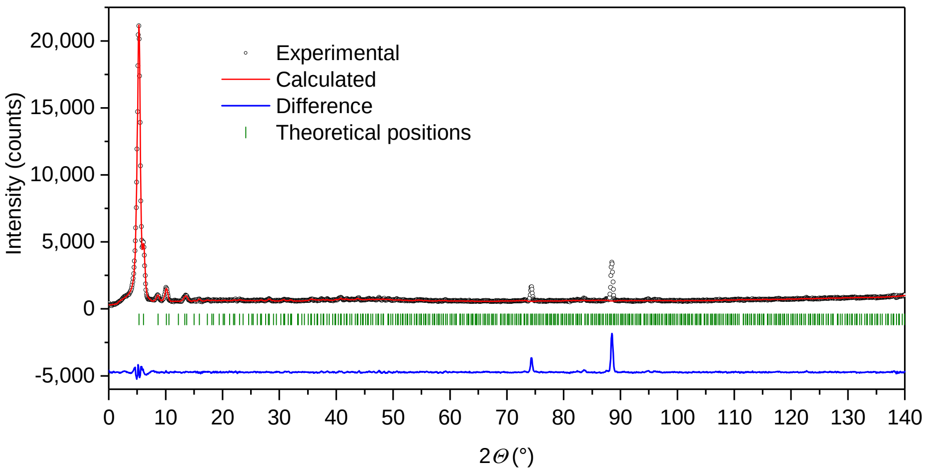

Supplementary Figure 53. Rietveld plot of DUT-50 with a loading of 488 CD_4 molecules per unit cell at 111 K.

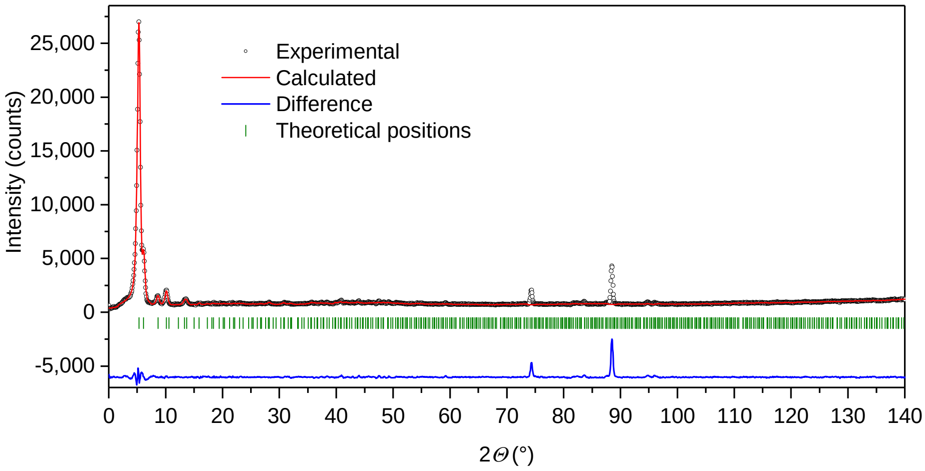

Supplementary Figure 54. Rietveld plot of DUT-50 with a loading of 529 CD_4 molecules per unit cell at 111 K.

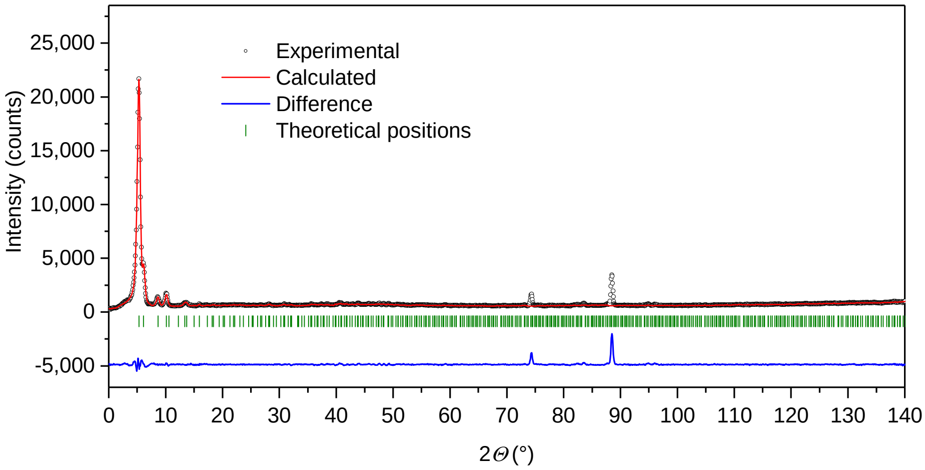

Supplementary Figure 55. Rietveld plot of DUT-50 with a loading of 569 CD_4 molecules per unit cell at 111 K.

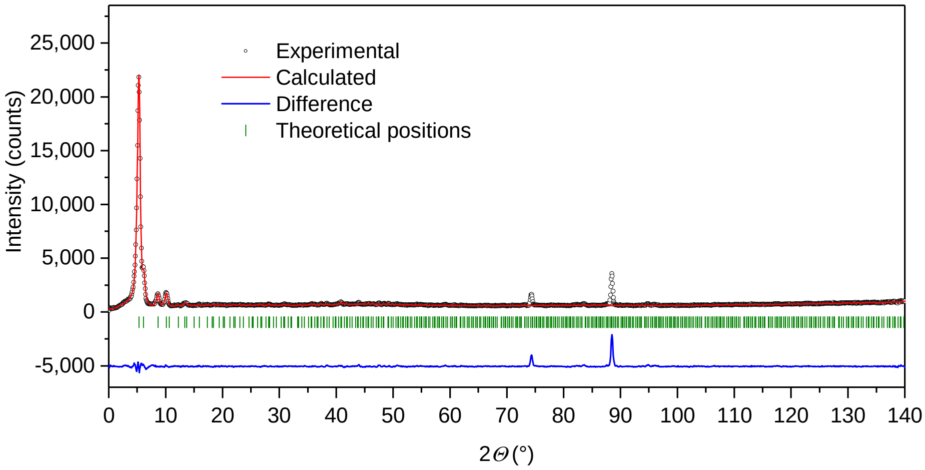

Supplementary Figure 56. Rietveld plot of DUT-50 with a loading of 683 CD_4 molecules per unit cell at 111 K.

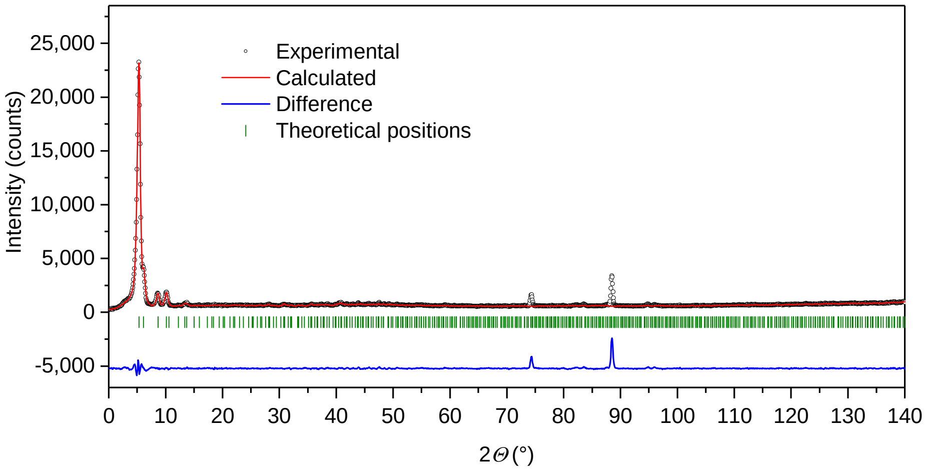

Supplementary Figure 57. Rietveld plot of DUT-50 with a loading of 756 CD_4 molecules per unit cell at 111 K.

Supplementary Table 24. Crystallographic information for DUT-50.

Material ID	DUT-50
Chemical formula, weight	$Cu_2O_8C_{46}H_{24}N_2$, 859.7 g mol^{-1}
Empirical formula, weight	$Cu_{48}O_{192}C_{960}H_{480}N_{48}$, 20632.8 g mol^{-1}
Temperature	111 K
Wavelength	Neutrons; 2.8172(2) Å
Measurement system	E9-FIREPOD at the BER II neutron source, Helmholtz Zentrum Berlin
Crystal system	Cubic
Space group	$Fm\bar{3}m$ (No. 225)
Unit cell dimensions	52.744(6) Å
Volume	146,731(30) Å^3
Z	24
Density	0.234 g cm^{-3}
2Θ range for data collection	-9.40873 ° – 141.79128 °
2Θ resolution	0.075 °
No. of data points	2017
Refinement method, program	Rietveld L.S. refinement, Full Prof

Supplementary Table 25. Lattice and profile parameters for Rietveld refinements of CD_4 loaded DUT-50.

Suppl. Figure	a (Å)	R_p	R_{wp}	R_{exp}	χ^2	R-Bragg	CD_4 per UC exp.	CD_4 per UC refined
Supplementary Figure 49	52.852(9)	3.16	4.35	2.29	3.61	4.83	161	159
Supplementary Figure 50	52.852(9)	3.58	4.79	3.58	1.79	3.66	230	228.5
Supplementary Figure 51	52.785(8)	3.57	4.84	3.53	1.88	3.09	280	280
Supplementary Figure 52	52.803(8)	3.71	4.90	3.50	1.96	3.19	369	370
Supplementary Figure 53	52.839(8)	3.51	4.62	3.46	1.78	2.80	485	488
Supplementary Figure 54	52.855(8)	3.42	4.61	3.08	2.24	2.41	530	529
Supplementary Figure 55	52.808(7)	3.68	4.87	3.44	2.01	3.37	563	569
Supplementary Figure 56	52.789(5)	3.46	4.75	3.43	1.92	2.60	625	683
Supplementary Figure 57	52.814(5)	3.62	4.81	3.42	1.97	2.64	751	756

Supplementary Table 26. Fractional coordinates of asymmetric unit in guest-free DUT-50 from NPD data.

		Coordinates				
Atom-ID	Atom	*x*	*y*	*z*	U_{iso}	Occupancy
CuA	Cu	0.00000	0.00000	0.36786	0.01299	1.00000
CuB	Cu	0.00000	0.00000	0.31786	0.01299	1.00000
OA	O	0.00000	0.03667	0.36410	0.01316	1.00000
OB	O	0.00000	0.03640	0.32170	0.01316	1.00000
NA	N	0.00000	0.15413	0.34587	0.01316	1.00000
CA	C	0.00000	0.04761	0.34295	0.01408	1.00000
CB	C	0.00000	0.07581	0.34276	0.01408	1.00000
CC	C	0.00000	0.08978	0.36513	0.01408	1.00000
HC	H	0.00000	0.08142	0.38070	0.04711	1.00000
CD	C	0.00000	0.11591	0.36464	0.01408	1.00000
CE	C	0.00000	0.12848	0.34150	0.01408	1.00000
CF	C	0.00000	0.11548	0.31819	0.01408	1.00000
HF	H	0.00000	0.12408	0.30280	0.04711	1.00000
CG	C	0.00000	0.08972	0.31925	0.01408	1.00000
HG	H	0.00000	0.08069	0.30410	0.04711	1.00000
CH	C	0.00000	0.17419	0.32582	0.01408	1.00000
CI	C	0.01959	0.19147	0.32550	0.01408	0.50000
HI	H	0.03271	0.19067	0.33730	0.04711	0.50000
CJ	C	0.01923	0.21045	0.30646	0.01408	0.50000
HJ	H	0.03198	0.22260	0.30580	0.04711	0.50000
CK	C	0.00000	0.21100	0.28901	0.01408	1.00000
CL	C	0.00000	0.23071	0.26927	0.01408	1.00000
CM	C	0.01898	0.24811	0.26740	0.01408	0.50000
HM	H	0.03229	0.24750	0.27900	0.04711	0.50000

Supplementary Table 27. Fractional coordinates of CD_4 molecules in DUT-50 at varied loadings.

CD_4 per UC	Atom-ID	Atom	*x*	*Y*	*z*	U_{iso}	Occupancy
159	AD1	C	0.11968	0.11967	0.56437	0.01408	0.96(3)
159	BD1	C	0.07280	0.07280	0.47208	0.01408	0.69(3)
218.5	AD1	C	0.14298	0.14297	0.56304	0.01408	0.57(5)
218.5	BD1	C	0.06427	0.06427	0.47982	0.01408	0.45(2)
218.5	CD1	C	0.04005	0.04005	0.27431	0.01408	0.55(4)
218.5	DD1	C	0.07279	0.07279	0.36556	0.01408	0.81(6)
280	AD1	C	0.14736	0.14735	0.56155	0.01408	0.76(3)
280	BD1	C	0.06517	0.06517	0.50000	0.01408	1.000(16)
280	CD1	C	0.04064	0.04064	0.27612	0.01408	0.66(4)
280	DD1	C	0.07366	0.07366	0.36343	0.01408	1.000(19)
370	AD1	C	0.03285	0.03285	0.39258	0.01408	0.81(2)
370	BD1	C	0.11573	0.11573	0.43472	0.01408	1.000(12)
370	CD1	C	0.12766	0.06434	0.29850	0.01408	0.56(3)
370	DD1	C	0.23252	0.03077	0.42897	0.01408	0.46(2)
488	AD1	C	0.02736	0.06837	0.40339	0.01408	0.592(17)
488	BD1	C	0.12289	0.06805	0.34789	0.01408	0.66(3)
488	CD1	C	0.13594	0.05012	0.27364	0.01408	0.54(7)
488	DD1	C	0.22437	0.04370	0.41114	0.01408	0.75(7)
529	AD1	C	0.02705	0.06656	0.39994	0.01408	0.61(2)
529	BD1	C	0.11927	0.07254	0.34550	0.01408	0.65(4)
529	CD1	C	0.14860	0.04684	0.26776	0.01408	0.67(5)
529	DD1	C	0.22223	0.04669	0.41533	0.01408	0.83(5)
569	AD1	C	0.02565	0.06478	0.39720	0.01408	0.651(20)
569	BD1	C	0.12241	0.07566	0.34224	0.01408	0.67(4)
569	CD1	C	0.14852	0.04426	0.26688	0.01408	0.79(5)
569	DD1	C	0.22133	0.04777	0.41810	0.01408	0.86(5)
683	AD1	C	0.03463	0.03463	0.39225	0.01408	1.00000
683	BD1	C	0.11483	0.11483	0.42957	0.01408	1.00000
683	CD1	C	0.02847	0.18312	0.23351	0.01408	0.5592(12)
683	DD1	C	0.03934	0.03934	0.26322	0.01408	1.0000(12)
683	ED1	C	0.12497	0.12497	0.30003	0.01408	1.0000(12)

683	FD1	C	0.29528	0.39076	0.45415	0.01408	1.0000(12)
756	AD1	C	0.04137	0.04137	0.39096	0.01408	1.0
756	BD1	C	0.10866	0.10866	0.43153	0.01408	1.0
756	CD1	C	0.04163	0.14674	0.23557	0.01408	0.94(3)
756	DD1	C	0.04855	0.04855	0.25574	0.01408	1.00(3)
756	ED1	C	0.12880	0.12880	0.29711	0.01408	1.00(3)
756	FD1	C	0.31567	0.39142	0.44913	0.01408	1.00(3)

Supplementary Note 9: *In situ*-DRIFT spectroscopy

Assignment of vibrational modes of each individual peak in the IR spectra of DUT-48, -46, -49, -50, and -151*int* is difficult and elaborate DFT analysis is beyond the scope of this study. However, each MOF and ligand contains the same 9*H*-carbazole-3,6-dicarboxylate building-block and a C-N bond to an aromatic bridging unit. Vibrational modes of these units will show the same peak positions in the IR spectra. In fact, several peaks with minor variation in wave number can be found in the spectra of all compounds analysed. Peaks which are found for all materials and can be assigned to vibrational modes of C-H, C-C, and C-N vibrations in the ligand backbone are of special interest as they are expected to be impacted by ligand buckling upon structural transition. These peaks are summarized in Supplementary Table 28. Air sensitive samples were analysed in a HARRICK Praying Mantis reaction chamber sealed by a removable dome with IR-transparent ZnSe windows. The same setup was used to perform *in situ* DRIFT analysis by applying a constant flow of a mixture of inert carrier gas (N_2) and adsorptive (*n*-butane or vaporized CCl_4). For the analysis of *n*-butane adsorption, the gas composition was controlled via two mass flow controllers (MFC). The composition during *in situ* adsorption of vaporized CCl_4 was estimated by two valves controlling the flux of inert and CCl_4 saturated inert gas.

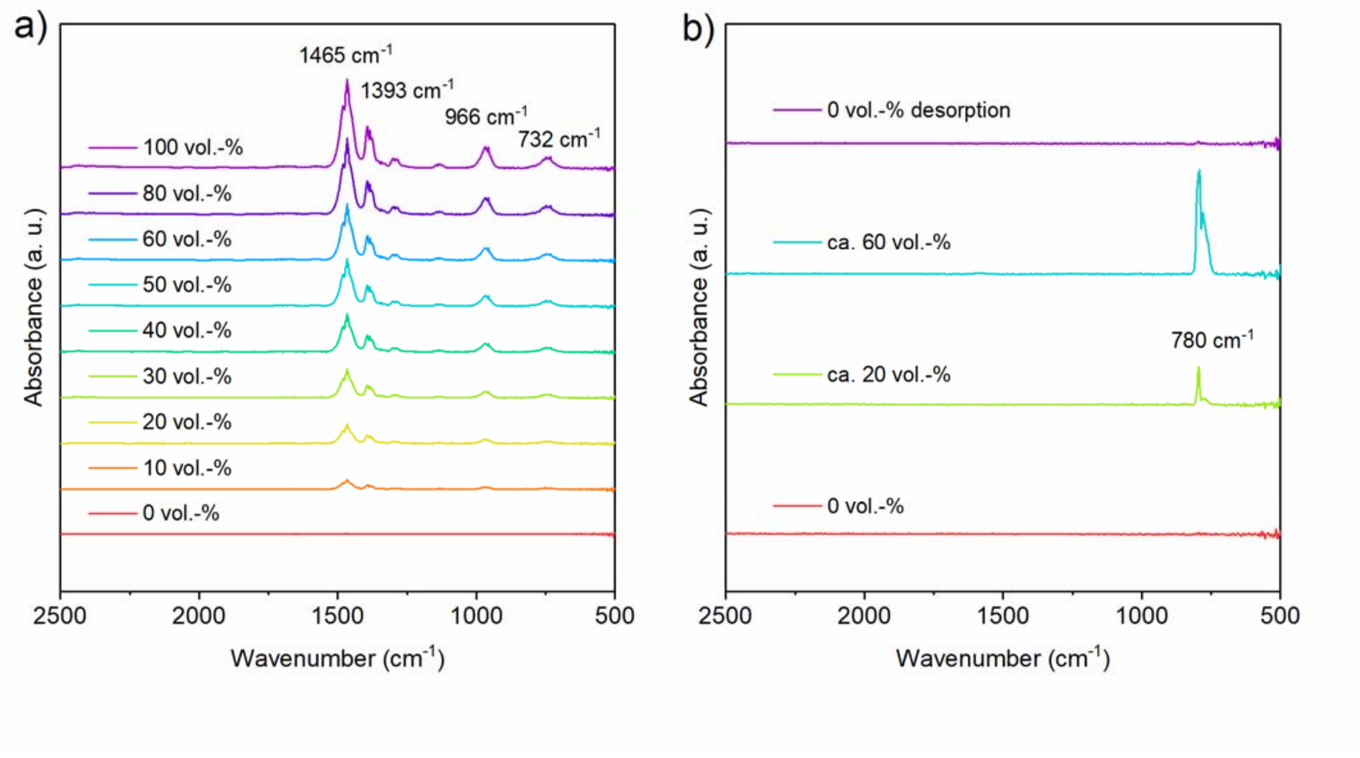

Supplementary Figure 58. a) DRIFT spectra of KBr filled sample holder at different concentrations of *n*-butane at 299 K and b) varying concentrations of CCl_4 vapour in nitrogen.

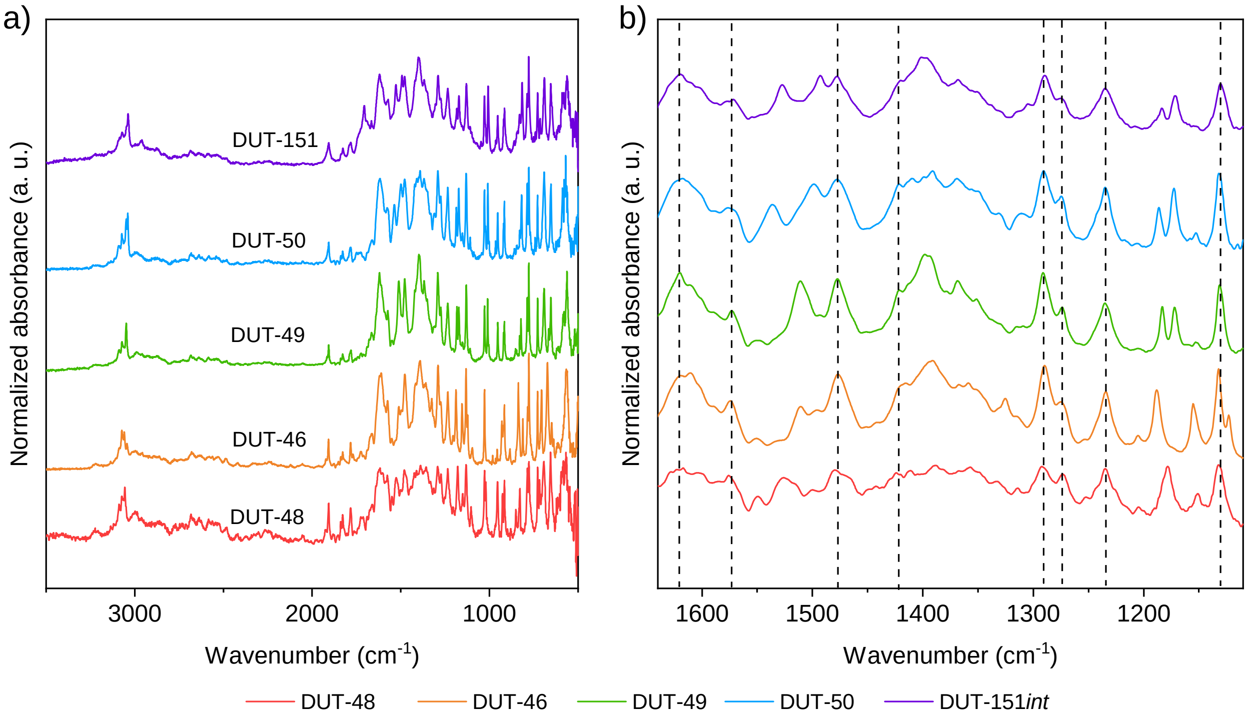

Supplementary Figure 59. a) DRIFT spectra of DUT-48 (red), DUT-46 (orange), DUT-49 (green), DUT-50 (blue), and DUT-151*int* (purple). b) magnified region of vibrations assigned to the ligand backbone. Vertical dashed lines indicate vibrations found in all materials which are thus assigned to the carbazole cores.

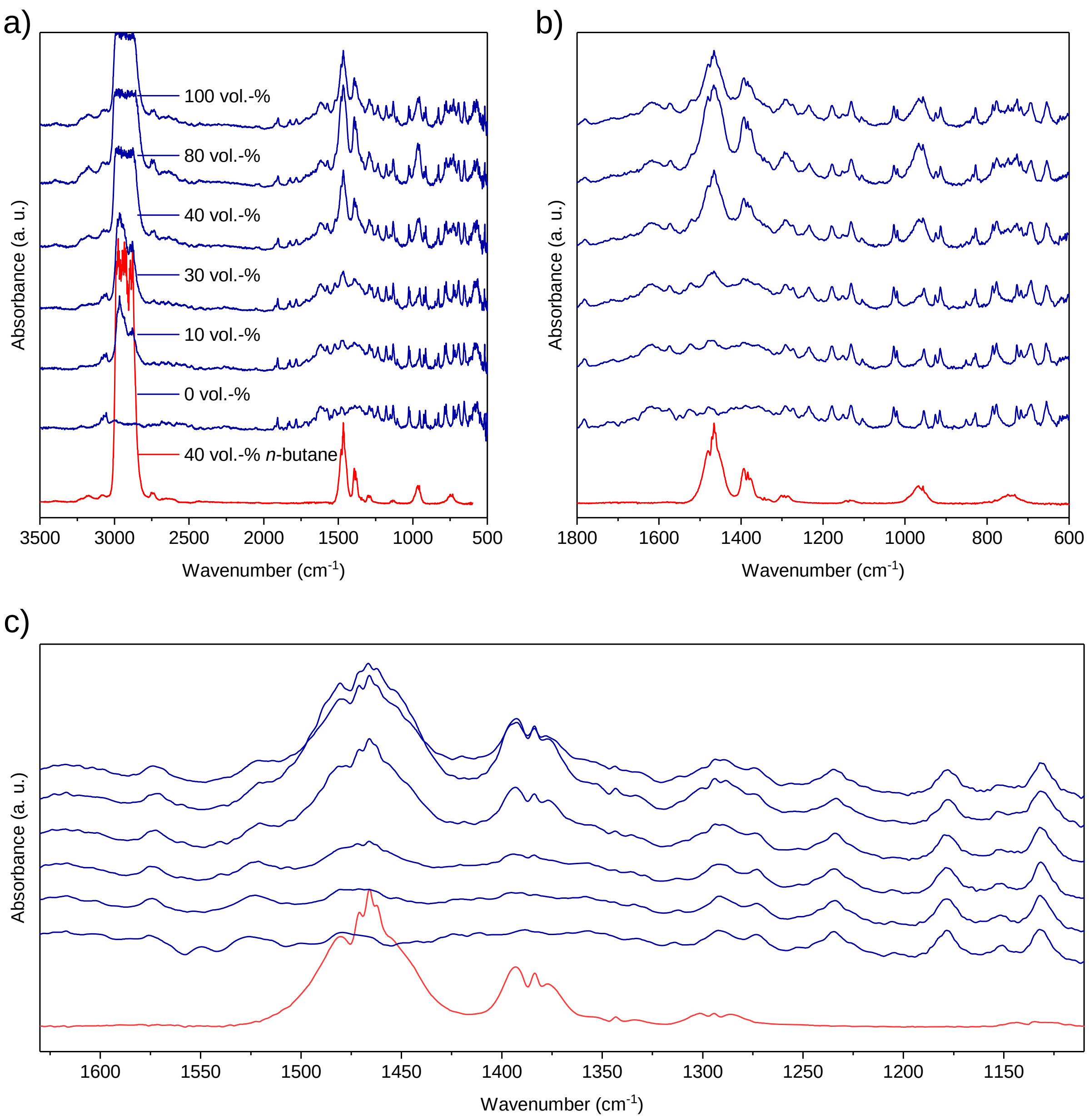

Supplementary Figure 60. a) *In situ*-DRIFT spectra of DUT-48 at varied loadings of *n*-butane at 299 K and b,c) magnifications of fingerprint region.

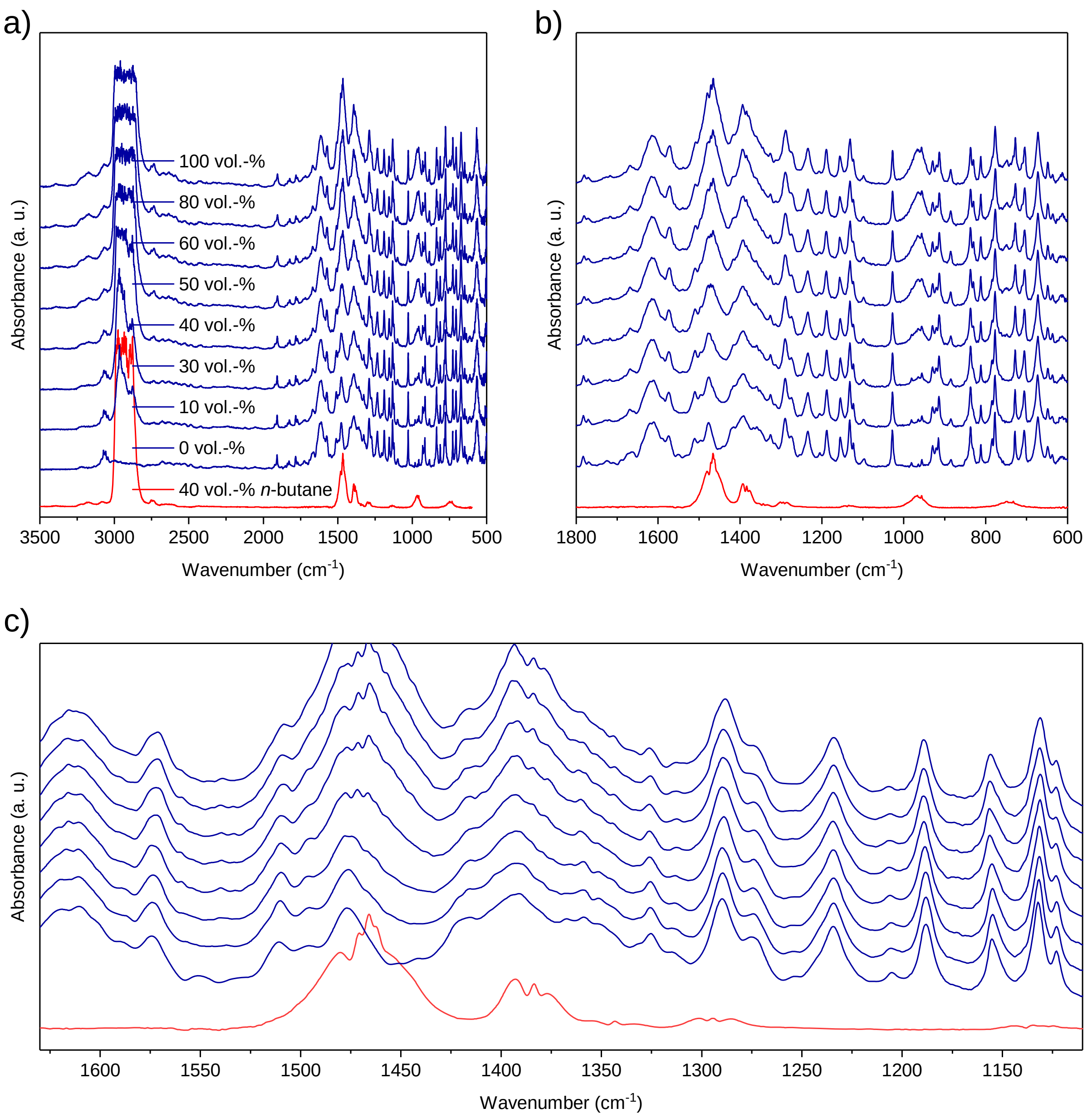

Supplementary Figure 61. a) *In situ*-DRIFT spectra of DUT-46 at varied loadings of *n*-butane at 299 K and b,c) magnifications of fingerprint region.

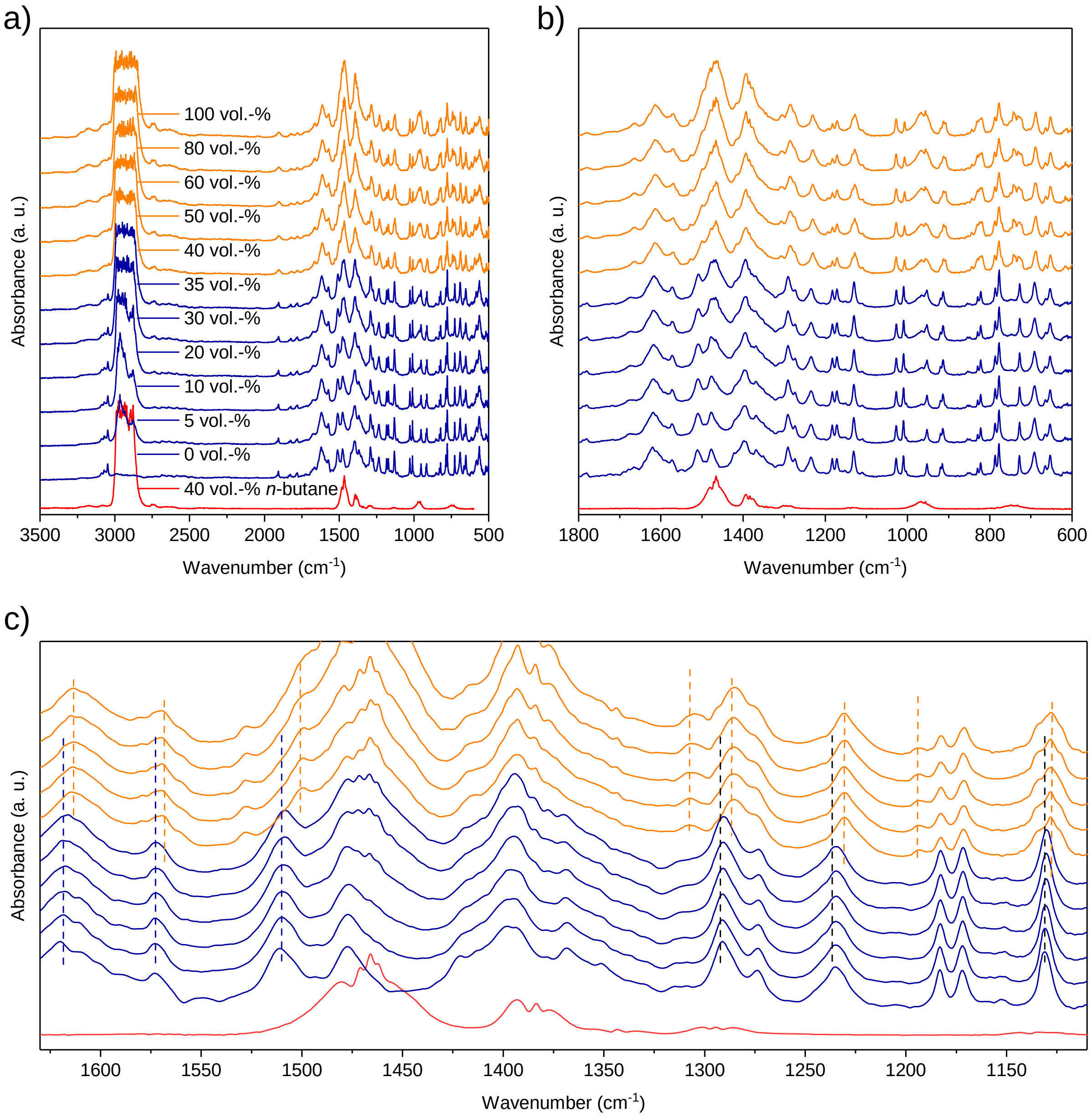

Supplementary Figure 62. a) *In situ*-DRIFT spectra of DUT-49 at varied loadings of *n*-butane at 299 K and b,c) magnifications of fingerprint region. Color code: blue: before transition, orange: after transition, dashed lines indicate shifts of peaks.

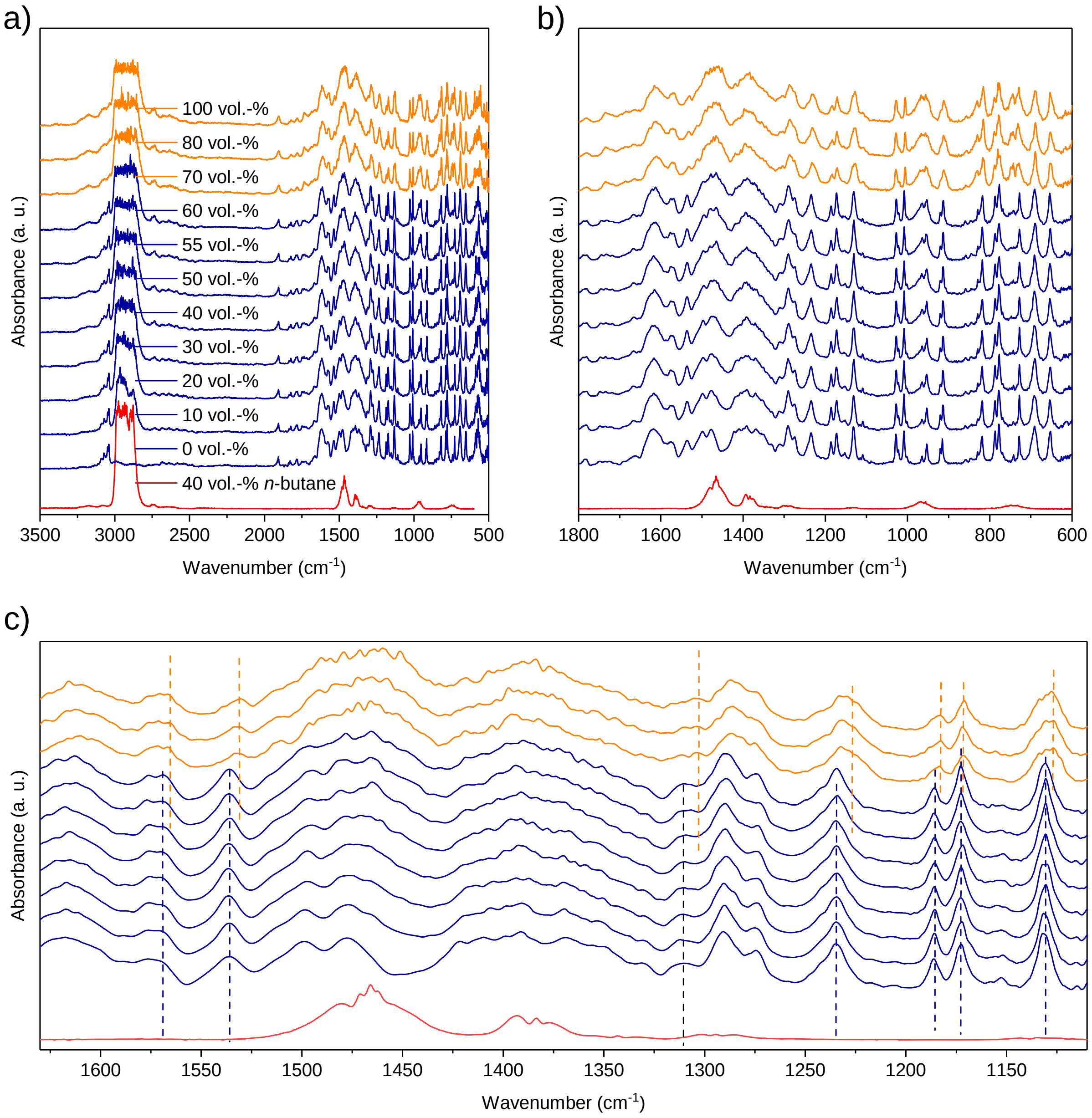

Supplementary Figure 63. a) *In situ*-DRIFT spectra of DUT-50 at varied loadings of *n*-butane at 299 K and b,c) magnifications of fingerprint region. Color code: blue: before transition, orange: after transition, dashed lines indicate shifts of peaks.

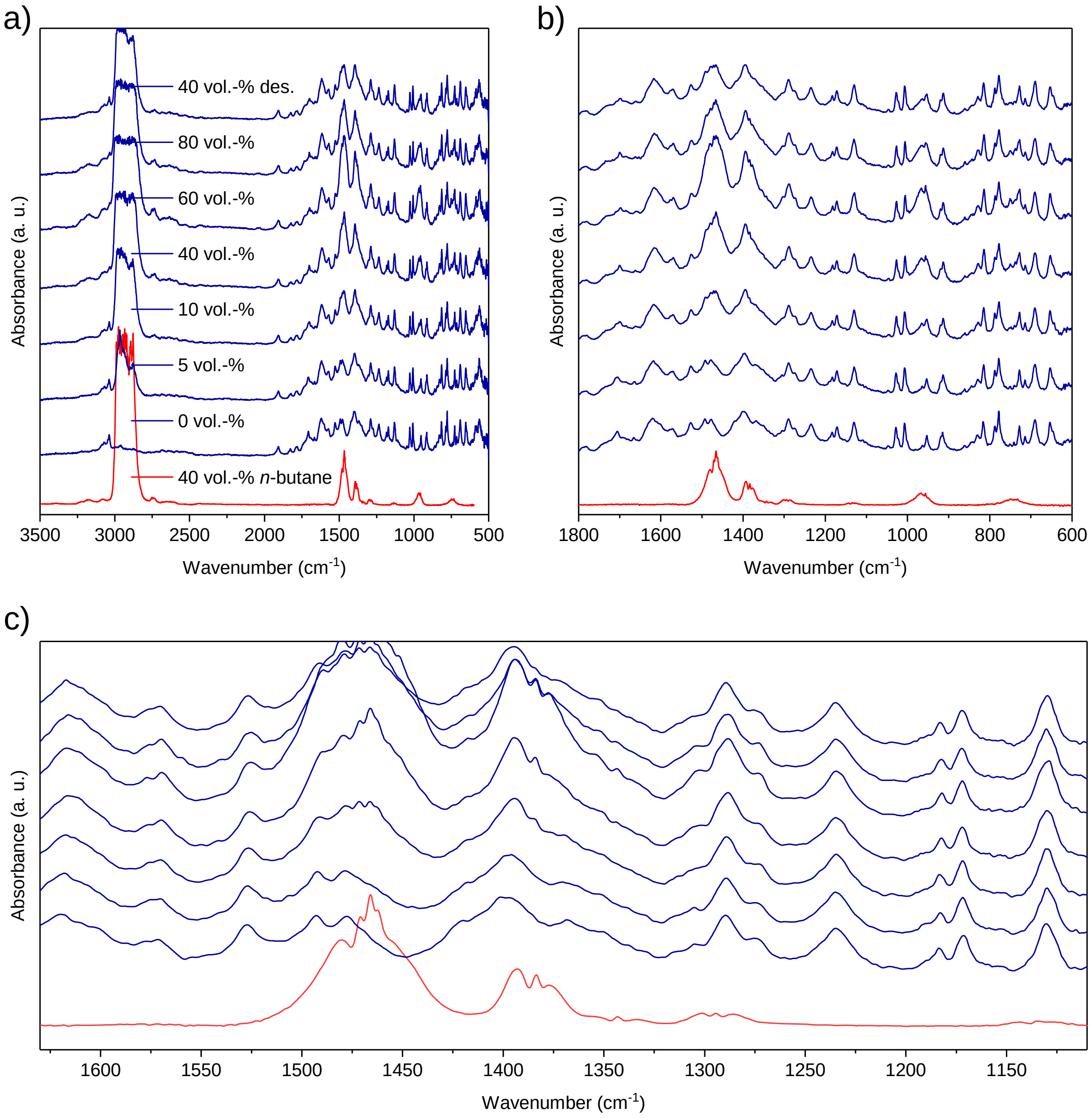

Supplementary Figure 64. a) *In situ*-DRIFT spectra of DUT-151 at varied loadings of *n*-butane at 299 K and b,c) magnifications of fingerprint region.

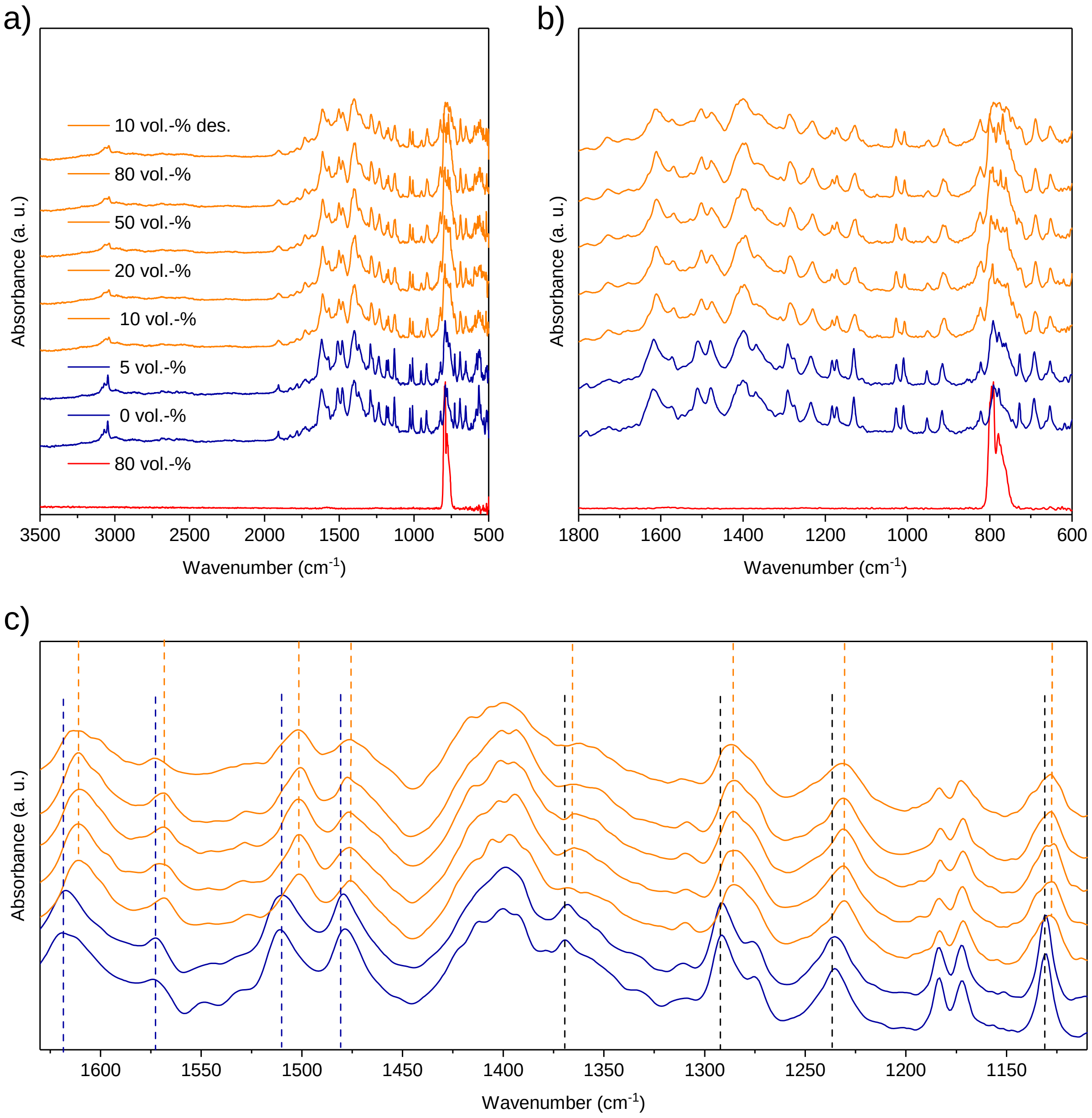

Supplementary Figure 65. a) *In situ*-DRIFT spectra of DUT-49 at varied loadings of CCl_4 at 299 K and b,c) magnifications of fingerprint region. Color code: blue: before transition, orange: after transition, dashed lines indicate shifts of peaks.

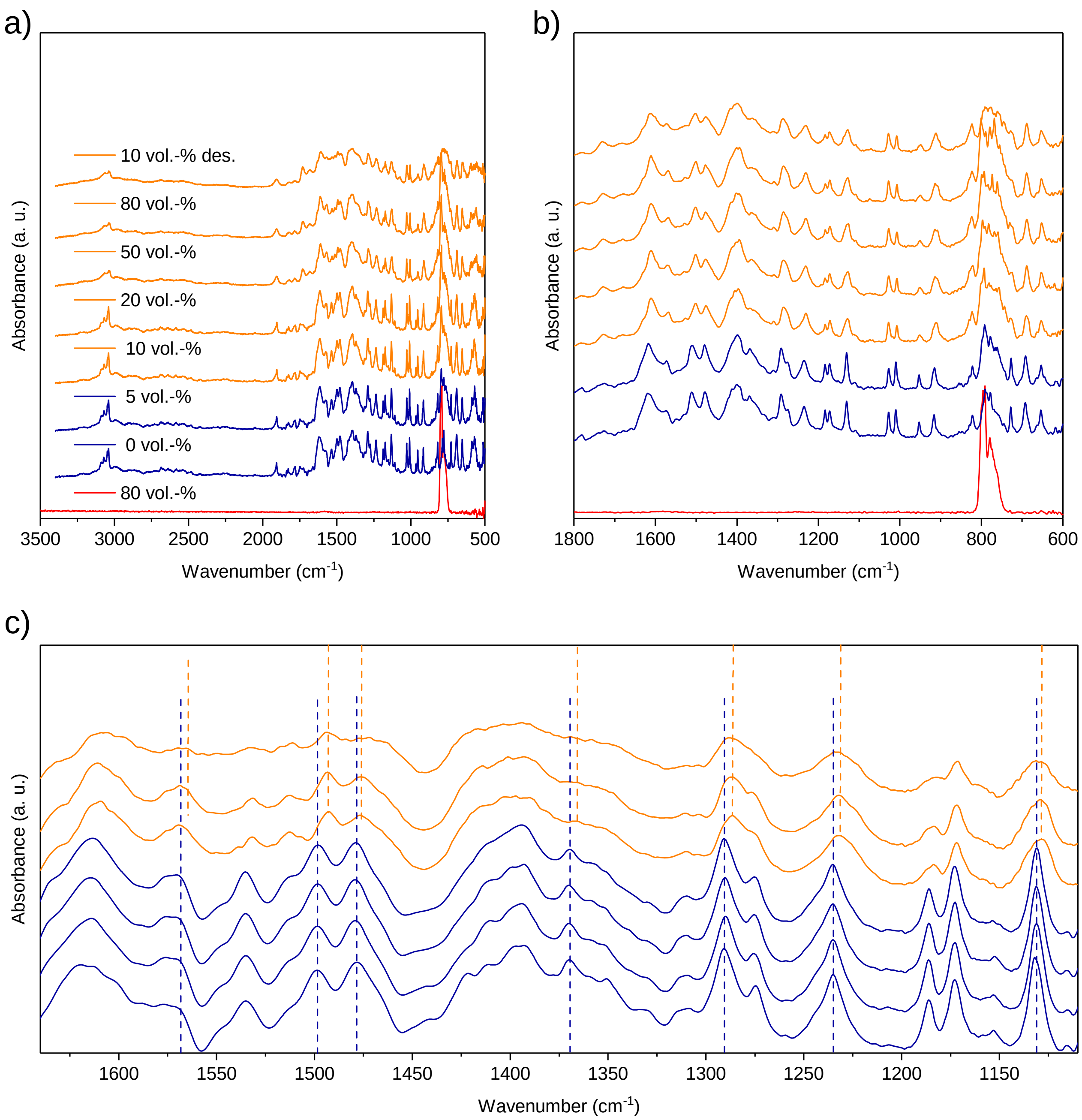

Supplementary Figure 66. a) *In situ*-DRIFT spectra of DUT-50 at varied loadings of CCl_4 at 299 K and b,c) magnifications of fingerprint region. Color code: blue: before transition, orange: after transition, dashed lines indicate shifts of peaks.

For DUT-49 and DUT-50, a shift in peak position to lower wavenumbers is observed upon adsorption of *n*-butane and CCl_4 which can be assigned to the structural contraction previously analysed by *in situ* PXRD. Several peaks are found to be affected in the spectra of DUT-49 in Supplementary Figure 62 (*n*-butane), Supplementary Figure 65 (CCl_4) and for DUT-50 in Supplementary Figure 63 (*n*-butane), Supplementary Figure 66 (CCl_4), respectively. The peaks which are shifted upon structural contraction are mostly different from the peaks found to be identical for the whole series of MOFs summarized in Supplementary Table 28. Consequently, they can most likely be assigned to changes of vibrational modes in the bridging units of the linkers and not the carbazole core.

Supplementary Table 28. Experimental peaks observed by *in situ* DRIFT spectroscopy in comparison with assigned peaks form reference [41].

Material ID	Wave number (cm^{-1})							
DUT-48	1610 (br)	1575	1478	1423	1291	1272	1234	1132
DUT-46	1620	1573	1476	1418	1289	1273	1234	1132
DUT-49	1619	1572	1508	1289	1234	n. a.	n. a.	1131
DUT-49 (CCl_4)	1610	1558	1501	1284	1230	n. a.	n. a.	1127
DUT-49 (*n*-butane)	1613	1569	1499	1286	1229	n. a.	n. a.	1128
DUT-50	1569	1479	1311	1290	1234	1185	1172	1131
DUT-50 (CCl_4)	1566	1475	1302	1285	1231	1182	1170	1128
DUT-50 (*n*-butane)	1565	1477	1303	1285	1231	1182	1170	1127
DUT-151	1619	1571	1477	1420	1289	1274	1233	1128
Reference [41]	1620	1497	1442	1364	1343	1299	1217	n. a.
Reference [41]-assigment[#]	$\nu(C–C)_b$, $\delta(C–H)_{ip}$	$\delta(C–H)_{ip,b}$, $\nu(C–N)$	$\delta(C-H)_{ip,c}$	$\delta(C–H)_{ip}$, $\nu(C–N)$	$\delta(C–H)_{ip}$, $\nu(C-N)$, $\nu(C–C)$	$\delta(C-H)_{ip}$	$\delta(C-H)_{ip,c}$	n. a.

[#] δ and ν refer to deformation and stretching modes, respectively. 'ip' indicate in plane modes. 'c' and 'b' indicate a dominant localization on the carbazole groups or biphenyl, respectively.

Supplementary Note 10: Solid-state MAS NMR studies

Solid-state NMR measurements were carried out on a Bruker Ascend 800 spectrometer equipped with a 3.2 mm HX probe. The ^{1}H-^{13}C cross polarization experiments were performed at a resonance frequency of 800.2 MHz for protons and 201.2 MHz for carbon. The contact time was 4 ms and the relaxation delay was 3 s. A sample rotation of 15 kHz was chosen. The ^{13}C chemical shift was referenced using adamantane.

The rotor was filled with the activated material (*op*-phase) under argon atmosphere. After NMR analysis of argon-filled DUT-49*op*, the rotor was opened under *n*-butane atmosphere and equilibrated for 2 min to obtain the *cp*-phase of the MOF. Formation of the *cp*-phase under these conditions was confirmed in a separate experiment by *in situ* PXRD. After sealing the rotor, the measurements of the butane filled *cp*-phase were carried out. Subsequently, the rotor was re-opened allowing the sample to have extended contact to ambient conditions and moisture which is known to initiate decomposition of the material. For these samples no reflections in the PXRD patterns could be observed.

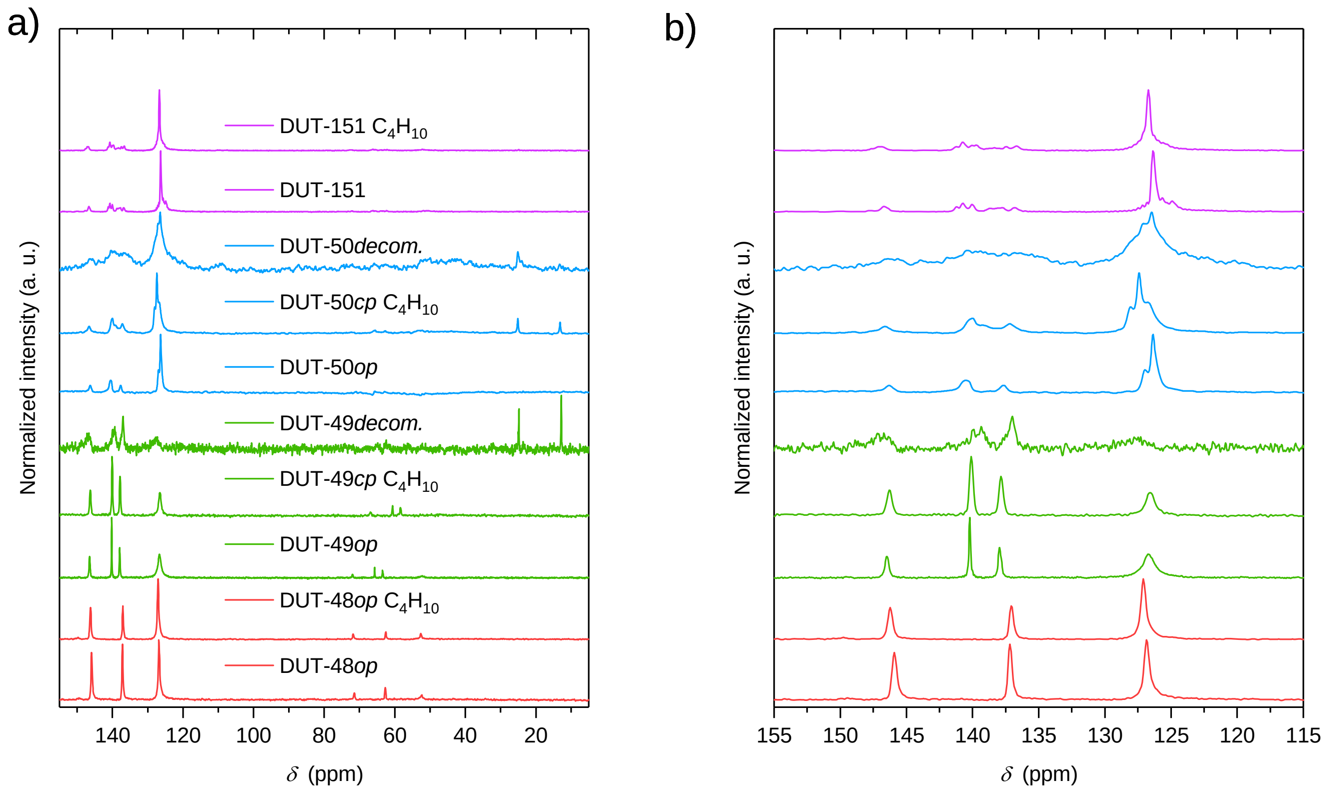

Supplementary Figure 67. Solid-state ^{13}C CP MAS NMR spectra for DUT-48, DUT-49, DUT-50 and DUT-151 filled with argon and *n*-butane at 298K.

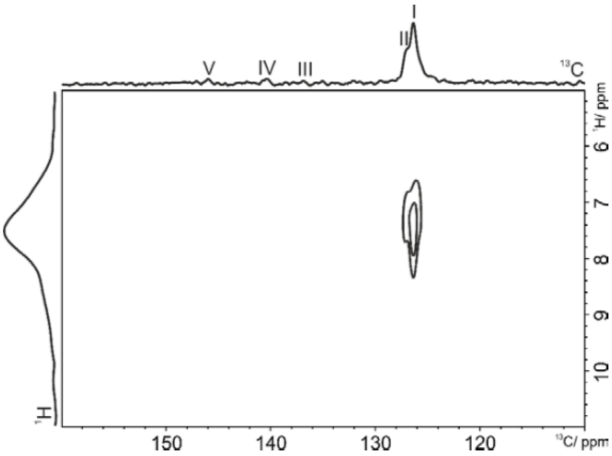

Supplementary Figure 68. ^{13}C-^{1}H-HETCOR- spectrum of Ar-filled DUT-50*op* at a contact time of 0.7 ms (ν_{rot} = 15 kHz).

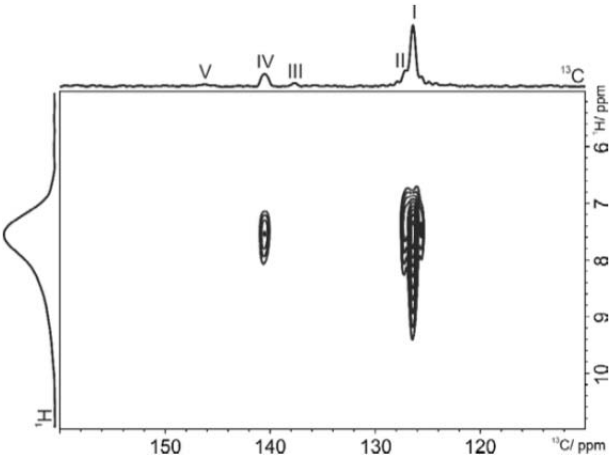

Supplementary Figure 69. ^{13}C-^{1}H-HETCOR spectrum of Ar-filled DUT-50*op* at a contact time of 4 ms (ν_{rot} = 15 kHz).

Supplementary Table 29. Peak assignment of ^{13}C NMR signals in DUT-50.

Signal	δ (ppm)	Assignment
I	126.3	CH
II	127.0	C
III	137.7	C
IV	140.5	C
V	146.3	C

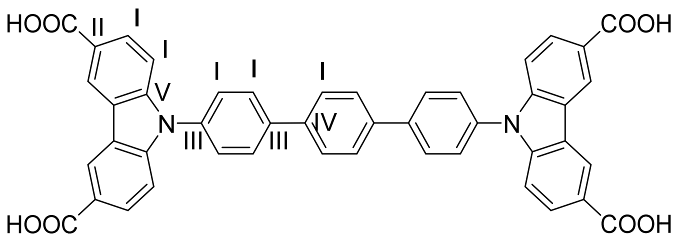

Supplementary Figure 70. Chemical structure of L_4 and assignment of ^{13}C NMR signals. The assignment of signal I to the CH groups and II-V to quaternary could be further corroborated by solid-state APT (attached proton test) experiments (data not shown). The assignment of signals II-V observed for intact DUT-50 to the designated carbon positions in the linker is suggested based on chemical shift predictions calculated with the computer program ChemDraw.

Supplementary Table 30. Unit cell parameter derived from structural modelling for the disorder free *op* phases in $Pa\overline{3}$ space group.

	DUT-48	DUT-46	DUT-49	DUT-50	DUT-151
Simulated unit cell volume ($Å^3$)	63570.67	79761	97153	141092	199196
Simulated lattice parameter, *a* (Å)	39.91	43.045	45.971	52.059	58.55
Experimental unit cell volume ($Å^3$)	66381(14)	82597(28)	101117(19)	145781(51)	n. a.
Experimental lattice parameter, *a* (Å)	40.490(5)	43.550(5)	46.588(5)	52.630(6)	n. a.

Supplementary Table 31. Structure parameter derived from the MD simulations.

	DUT-48	DUT-46	DUT-49	DUT-50	DUT-151
Global minimum (Unit cell volume *op*) ($Å^3$)	63044	78402	96071	142645	197645
Local minimum (Unit cell volume *cp*) ($Å^3$)	35287	41421	45882	58185	74988
Unit cell volume structural model *cp* ($Å^3$)	36674	41501	47360	57858	76227
Simulated transition pressure (MPa)	145	84	65	38	22
Free energy difference *op*-*cp* (kJ mol_{uc}^{-1})	892	793	768	598	336

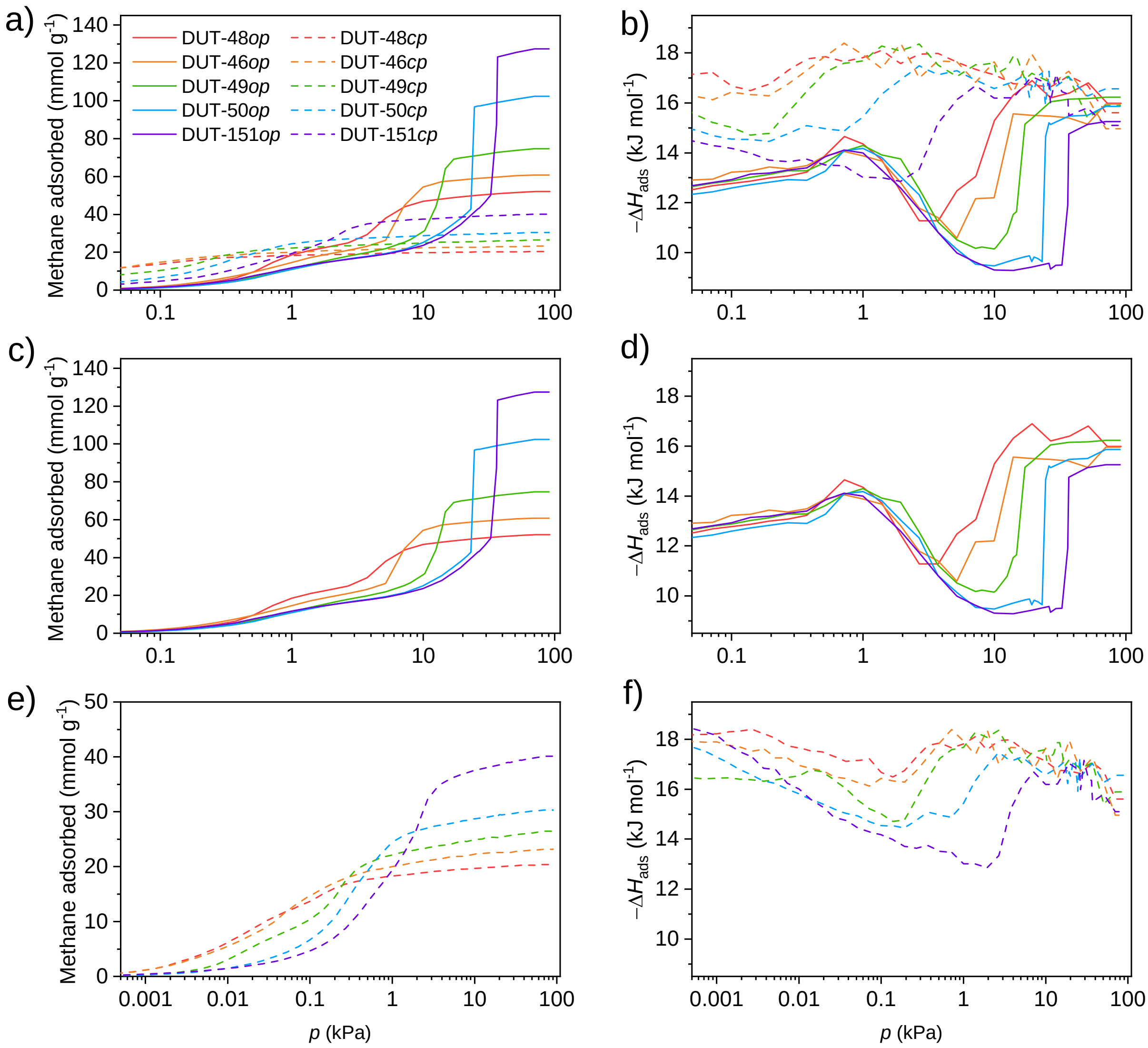

Supplementary Figure 71. a,c,e) Simulated methane adsorption isotherms at 111 K. b,d,f) Corresponding enthalpy profiles. Color code: DUT-48 (red), DUT-46 (orange), DUT-49 (green), DUT-50 (blue), DUT-151 (purple), *op* (full lines) and *cp* (dashed lines).

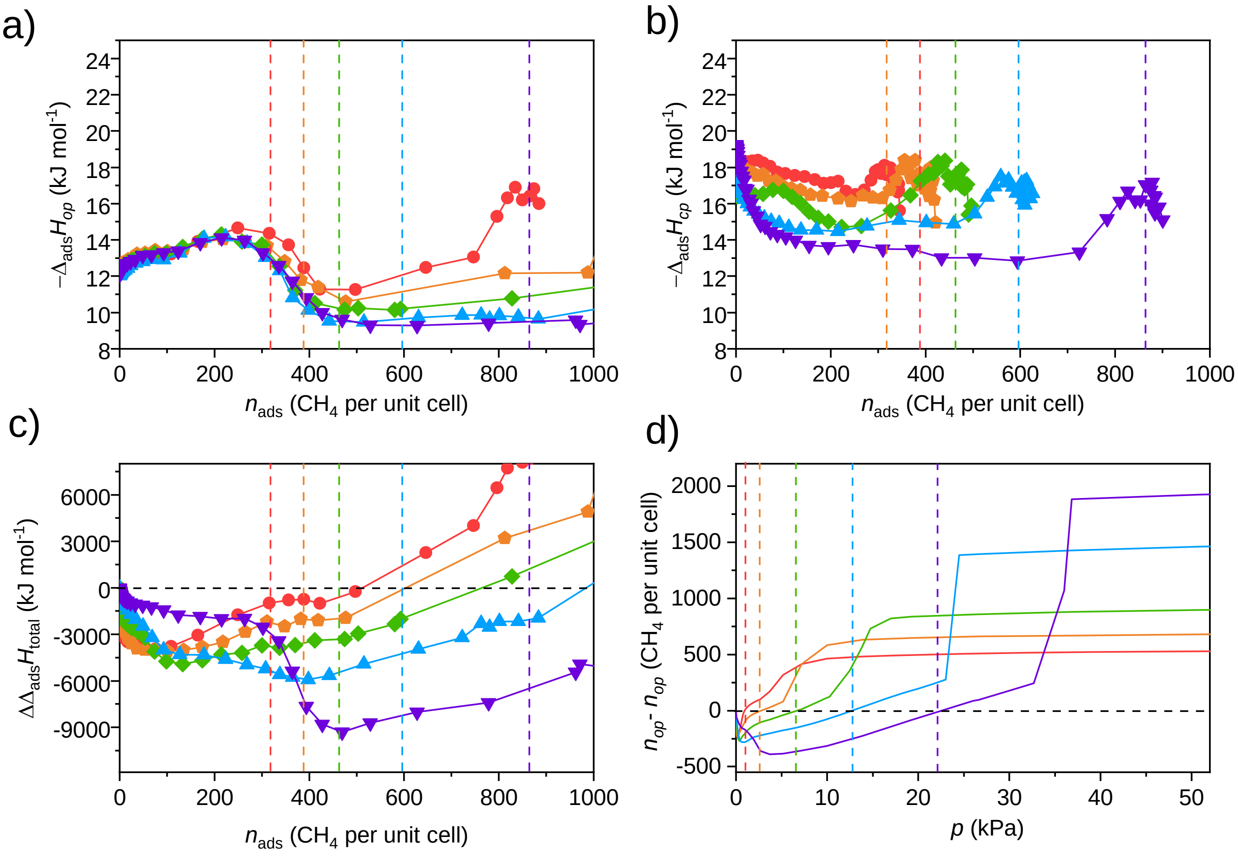

Supplementary Figure 72. a,b) Adsorption enthalpies for methane adsorption at 111 K plotted against the loading in the *op* (a) and *cp* phases (b) of DUT-48 (red), DUT-46 (orange), DUT-49 (green), DUT-50 (blue), DUT-151 (purple); c) evolution of $\Delta\Delta_{ads}H_{total}$ with loading and d) the difference in uptake between the op and cp phase plotted against pressure. Intersections of the op-cp isotherms are indicated as vertical dashed lines.

Supplementary Note 11: Thermodynamic considerations

At the intersection of the isotherms, $n_{ads} = n_{ads,op} = n_{ads,cp}$, indicated as dashed lines in Supplementary Figure 72, the difference in adsorption enthalpy of methane adsorbed in the *op* and *cp* phase increases from 3.7 kJ mol^{-1} methane in DUT-48 to 7.4 kJ mol^{-1} methane in DUT-50. The total gain in adsorption enthalpy upon structural contraction per unit cell, $\Delta\Delta_{ads}H_{total}$, at the intersection of the isotherms of *op* and *cp* phase can be estimated by multiplying the difference of adsorption enthalpy in the *op* and *cp* phase, $\Delta\Delta_{ads}H = \Delta_{ads}H_{cp} - \Delta_{ads}H_{op}$ with the amount of gas adsorbed per unit cell, n_{ads}.

$$\Delta\Delta_{ads}H_{total} = (\Delta_{ads}H_{cp} - \Delta_{ads}H_{op}) \cdot n_{ads} \quad (4)$$

Estimations of $\Delta\Delta_{ads}H_{total}$ for the series of materials are -1177 kJ mol^{-1} (DUT-48), -2289 kJ mol^{-1} (DUT-46), -3491 kJ mol^{-1} (DUT-49), -4458 kJ mol^{-1} (DUT-50), -6290 kJ mol^{-1} (DUT-151).

Supplementary Table 32. Adsorption enthalpy estimations for *op-cp* transitions derived from the GCMC simulations.

	DUT-48	DUT-46	DUT-49	DUT-50	DUT-151
p at intersection (kPa)	1.05	2.6	6.8	12.9	22.3
$n_{ads} = n_{ads,op} = n_{ads,cp}$ (molecules CH_4 per unit cell)	318	388	463	596	864
$\Delta\Delta_{ads}H = \Delta_{ads}H_{cp} - \Delta_{ads}H_{op}$ (kJ mol^{-1})	-3.7	-5.9	-7.54	-7.48	-7.28
$\Delta\Delta_{ads}H_{total} = (\Delta_{ads}H_{cp} - \Delta_{ads}H_{op}) \cdot n_{ads}$ (kJ mol^{-1})	-1177	-2289	-3491	-4458	-6290

Equation (4) can be generalized to reflect the adsorptive part of the energetic driving force for the entire pressure range $n_{ads,op} \neq n_{ads,cp}$. $\Delta\Delta_{ads}H_{total}$ is the total difference in adsorption enthalpy for the *op-cp* transition of one unit cell.

$$\Delta\Delta_{ads}H_{total} = \Delta_{ads}H_{cp} \cdot n_{ads,cp} - \Delta_{ads}H_{op} \cdot n_{ads,op} \quad (5)$$

The quantity

$$\Delta\Delta_{ads}H = \Delta_{ads}H_{cp} - \Delta_{ads}H_{op} < 0 \quad (6)$$

is the difference in adsorption enthalpies for the *op* and *cp* phase. Equation (5) can be rearranged to reflect the total adsorptive enthalpy difference with respect to $\Delta n = n_{op} - n_{cp}$.

$$\Delta\Delta_{ads}H_{total} = \Delta_{ads}H_{cp} \cdot n_{ads,cp} - \Delta_{ads}H_{op} \cdot (\Delta n + n_{ads,cp}) \quad (7)$$

$$\Delta\Delta_{ads}H_{total} = \Delta\Delta_{ads}H \cdot n_{ads,cp} - \Delta_{ads}H_{op} \cdot \Delta n \quad (8)$$

For the negative gas adsorption transition: $\Delta n = \Delta n_{NGA}$. In equation (8) $\Delta\Delta_{ads}H \cdot n_{ads,cp}$ represents the adsorptive driving force for the contraction and $\Delta_{ads}H_{op} \cdot \Delta n$ the driving force for the expansion.

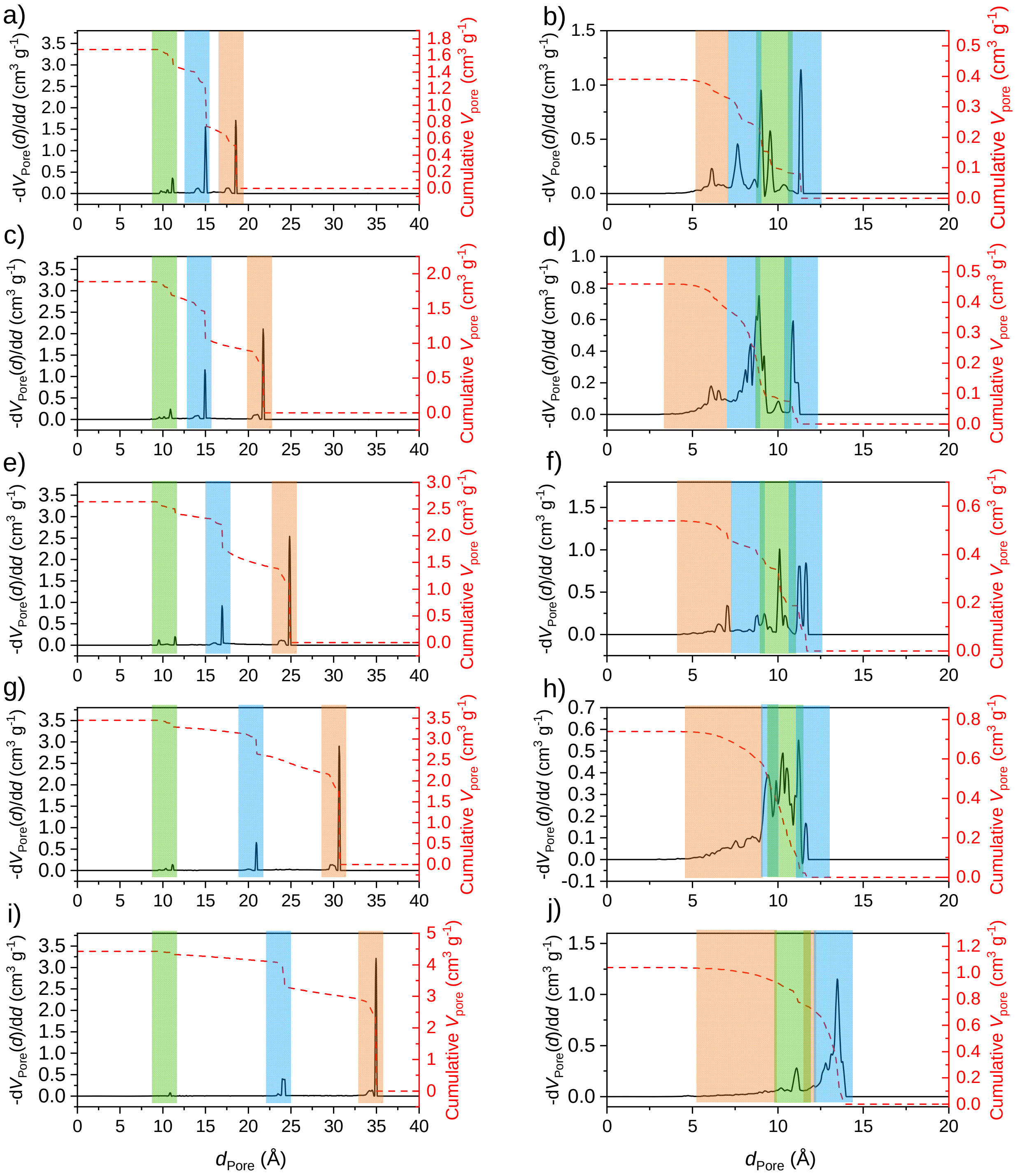

Supplementary Figure 73. Simulated pore size distributions (black lines) and cumulative pore volumes (red dashed lines) of a) DUT-48*op*, b) DUT-48*cp*, c) DUT-46*op*, d) DUT-46*cp*, e) DUT-49*op*, f) DUT-49*cp*, g) DUT-50*op*, h) DUT-50*cp*, i) DUT-151*op*, and j) DUT-151*cp*. Colour code trimodal pore system: octahedral (oct, orange), tetrahedral (tet, blue), cuboctahedral (cub, green).

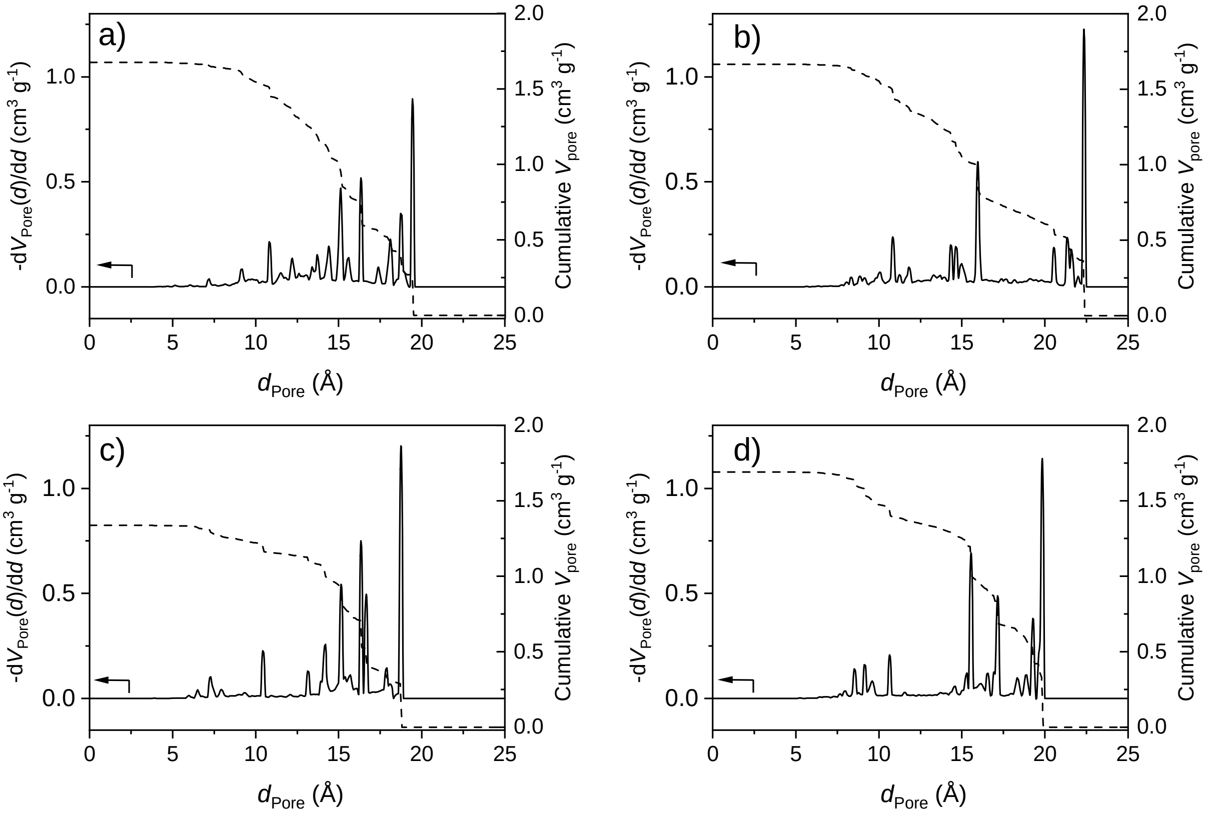

Supplementary Figure 74. Simulated pore size distributions (black lines) and cumulative pore volumes (red dashed lines) of a) DUT-151*int*, b) DUT-151*int-act*, c) DUT-151*int-cp,* and d) DUT-151*int-op*. Cumulative pore volume is given as dashed black line.

Supplementary Table 33. Pore size distributions and fractional pore volumes extracted from Supplementary Figure 73. Values for the *cp* phases were only partially estimated due to overlapping.

Material ID	Pore	*Open pore* V_{Pore} (cm^3 g^{-1})	d_{Pore} (Å)	V_{Pore} fract. (cm^3 g^{-1})	*Contracted pore* V_{Pore} (cm^3 g^{-1})	d_{Pore} (Å)	V_{Pore} fract. (cm^3 g^{-1})
DUT-48	Cub	total 1.67	10.8	0.27	total 0.39	10.9	n. a.
	Tet		15	0.62		n. a.	n. a.
	Oct		18.5	0.78		6.1	0.05
DUT-46	Cub	total 1.89	10.8	0.19	total 0.46	10.9	n. a.
	Tet		14.9	0.74		n. a.	n. a.
	Oct		21.7	0.96		6.1-6.5	0.08
DUT-49	Cub	total 2.64	10.8	0.23	total 0.54	10.9	n. a.
	Tet		16.9	0.87		n. a.	n. a.
	Oct		24.9	1.54		6.5-7	0.09
DUT-50	Cub	total 3.45	10.8	0.19	total 0.74	11	n. a.
	Tet		21	0.84		n. a.	n. a.
	Oct		30.7	2.43		6.5-8.5	0.13
DUT-151	Cub	total 4.43	10.8	0.14	total 1.04	11.1	n. a.
	Tet		24	1.16		n. a.	n. a.
	Oct		34.9	3.13		8.7-10.3	0.14

Supplementary Note 12: Adsorbate (CH_4) distribution and radial distribution analysis

Configuration snapshots from GCMC simulations were used to construct methane distribution for each of the three pores present in DUT-48, DUT-49 and DUT-50 at selected loadings (Supplementary Figure 75).

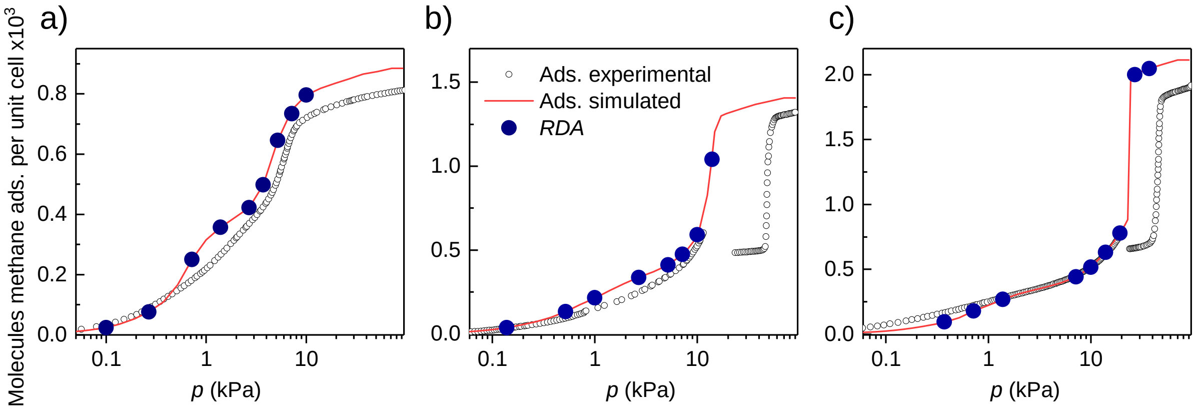

Supplementary Figure 75. Comparison of experimental (open symbols) and simulated (red line) methane adsorption isotherm at 111 K including selected loadings for radial distribution analysis (RDA) of a) DUT-48, b) DUT-49, and c) DUT-50.

Attributing each methane molecule to each of the pores was achieved via a python script using the pymatgen structure object (Supplementary Figure 76).

```python
pore_label = []
for index_methane in methane_indices:
      methane_cub_dist = []
      for index_cub in cub_indices:
            methane_cub_dist.append(structure.get_distance(index_cub, index_methane))
      methane_tet_dist = []
      for index_tet in tet_indices:
            methane_tet_dist.append(structure.get_distance(index_tet, index_methane))
      methane_oct_dist = []
      for index_oct in oct_indices:
            methane_oct_dist.append(structure.get_distance(index_oct, index_methane))

      if min(methane_cub_dist) < cub_cut:
            pore_label.append("cub")
            continue
      if min(methane_oct_dist) < oct_cut:
            pore_label.append("oct")
            continue
      if min(methane_tet_dist) < tet_cut:
            pore_label.append("tet")
            continue

amount_cub.append(pore_label.count("cub"))
amount_tet.append(pore_label.count("tet"))
amount_oct.append(pore_label.count("oct"))
```

Supplementary Figure 76. Python script using the pymatgen structure object for radial distribution analysis.

The script uses distance criteria between the adsorbate and the center of each pore to identify which pore the adsorbate is present within. We note that due to the aspherical nature of the pores the distributions are dependent on the sequence of applying the distance criteria. We choose to label in the order: cuboctahedral, octahedral and tetrahedral. This as the tetrahedral pore volume is both part of the cuboctahedral and octahedral pore volume. The key lines of the python code are displayed in Supplementary Figure 76 and a minimal working example is deposited in the supporting information. The amount of methane in each pore was further averaged over 100 snapshots. This was also repeated for structures resulting from the *in situ* CD_4 neutron diffraction experiments. The choice of cut-off for each of the pores was chosen by analysing the radial distribution of the framework atoms from the centre of each of the pores.

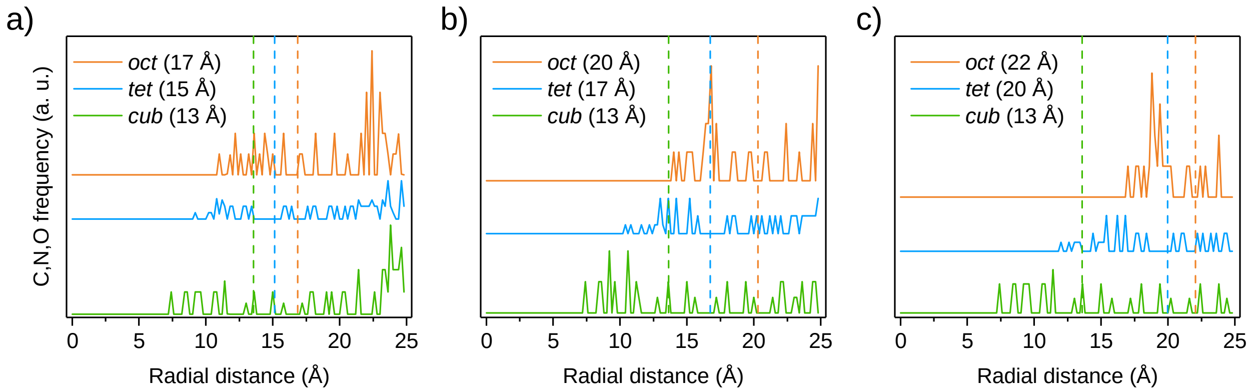

Supplementary Figure 77. Radial distribution of carbon, nitrogen, and oxygen atoms in the guest free framework of a) DUT-48, b) DUT-49, and c) DUT-50. Crystallographic origin of the octahedral pore (0.5,0.5,0.5), tetrahedral pore (0.25,0.25,0.25), and cuboctahedral pore (0,0,0). Dashed lines indicate cut off radius for the radial distribution analysis of methane filled MOFs.

Values of the chosen cut offs are displayed in Supplementary Figure 77 and summarized in Supplementary Table 34.

Supplementary Table 34. Cut-off radii for radial distribution analysis.

Material ID	*Cub* (Å)	*Tet* (Å)	*Oct* (Å)
DUT-48	10.6	12.4	14.4
DUT-49	10.6	14.6	17.2
DUT-50	10.6	17.4	19.8

To visualise the distribution of the adsorbates within each of the pores, the radial distribution relative to the pore was computed. This is similar to computing a radial distribution function but is not normalised to the radial volume as it is calculated from centre of the pore and at higher loadings the center point can contain a significant density of gas. The radial frequency of adsorbates and C, N, O atoms of the framework for each of the pores in DUT-48, DUT-49 and DUT-50 for increasing loading are displayed in Supplementary Figure 77 and Supplementary Figure 78. Furthermore, we report a comparison of the experimental and simulated distributions in Figure 79-81.

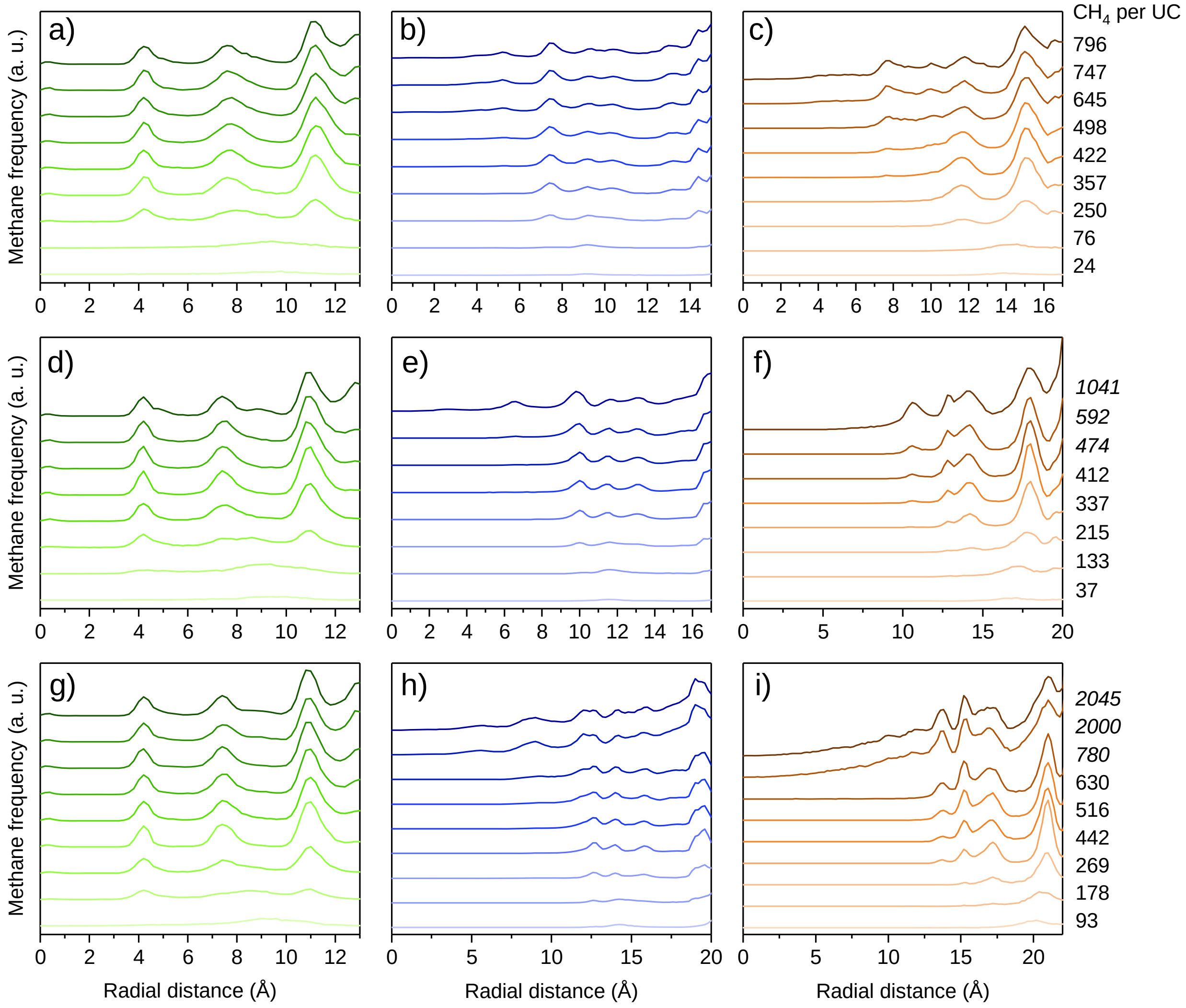

Supplementary Figure 78. Radial distribution of methane in the pores of a-c) DUT-48, d-f) DUT-49, and g-i) DUT-50. Crystallographic origin of the octahedral pore (orange, c,f,i) (0.5,0.5,0.5), tetrahedral pore (blue, b,e,h) (0.25,0.25,0.25), and cuboctahedral pore (green, a,d,g) (0,0,0). Loadings of the corresponding distribution curves are indicated at the right.

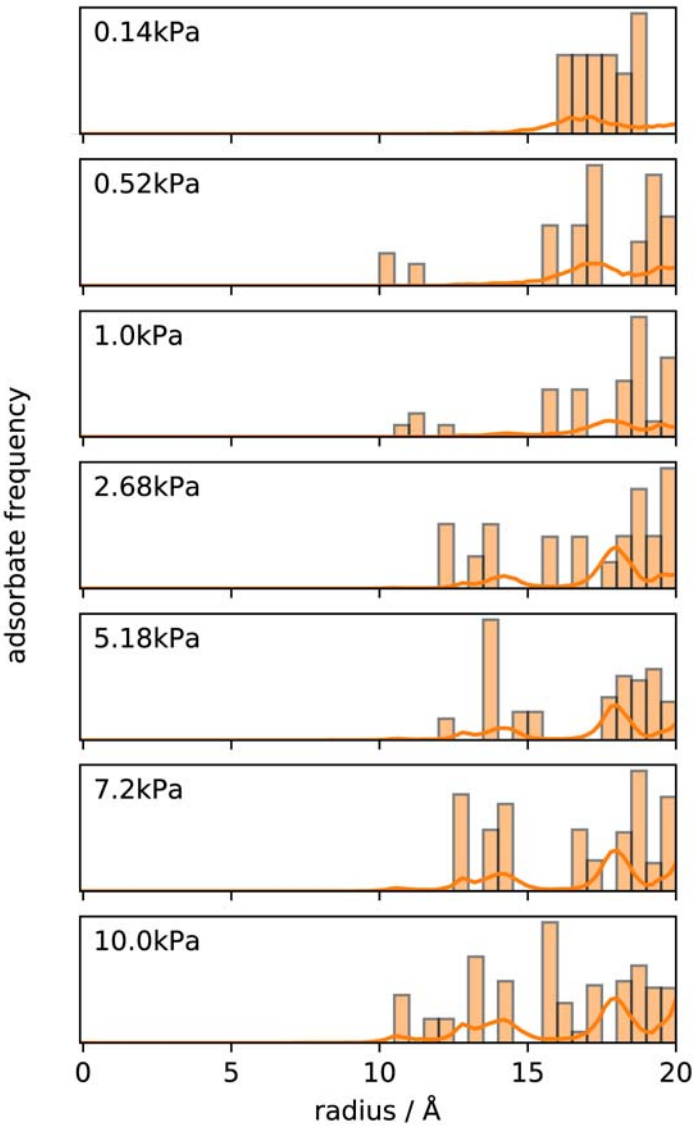

Supplementary Figure 79. Comparison of the simulated and experimental radial distributions of methane in the octahedral pore of DUT-49 for increasing loading. Simulated distributions are depicted as lines and experimental positions displayed as histograms.

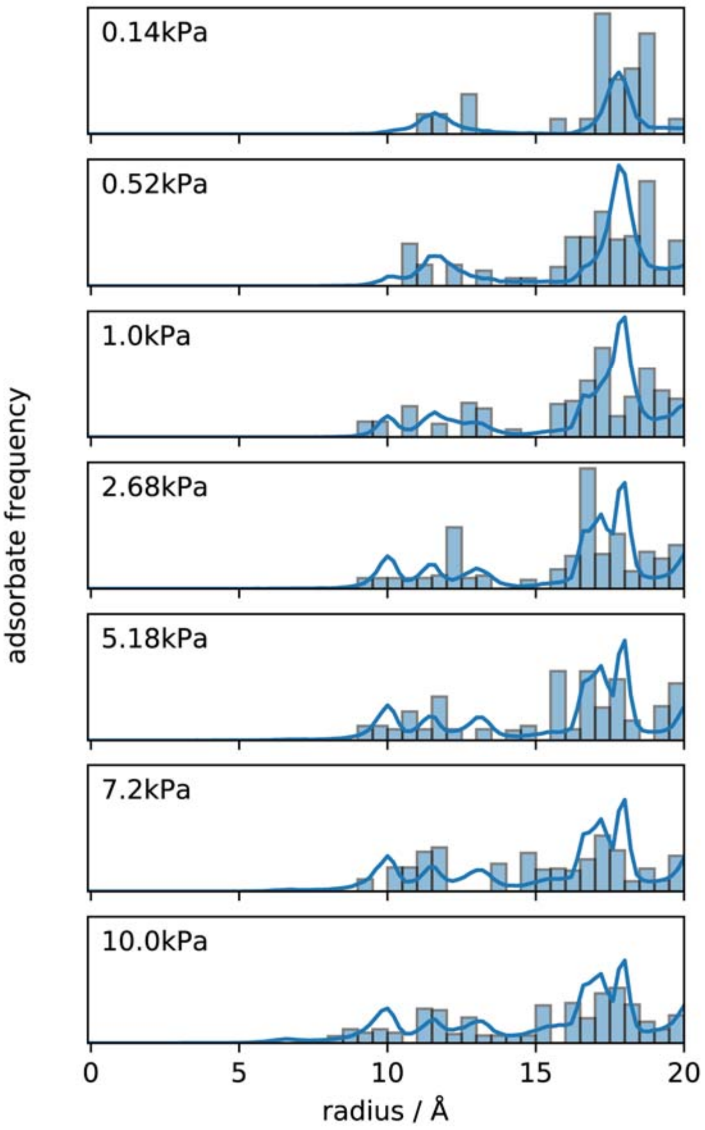

Supplementary Figure 80. Comparison of the simulated and experimental radial distributions of methane in the tetrahedral pore of DUT-49 for increasing loading. Simulated distributions are depicted as lines and experimental positions displayed as histograms.

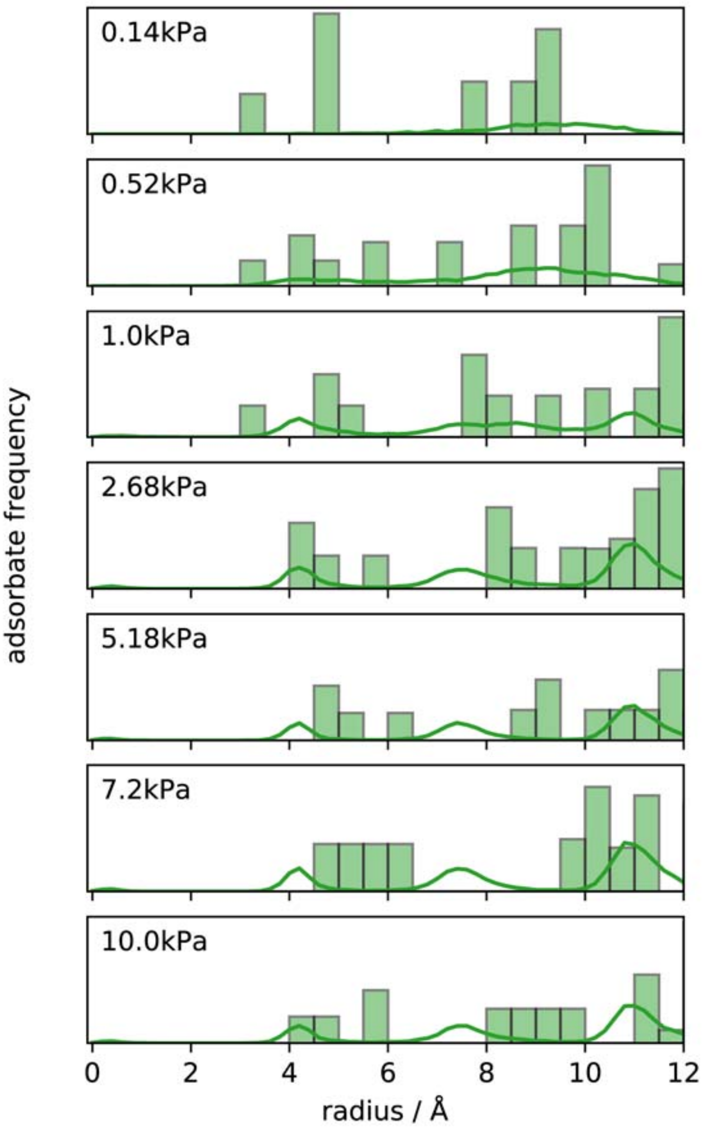

Supplementary Figure 81. Comparison of the simulated and experimental radial distributions of methane in the cuboctahedral pore of DUT-49 for increasing loading. Simulated distributions are depicted as lines and experimental positions displayed as histograms.

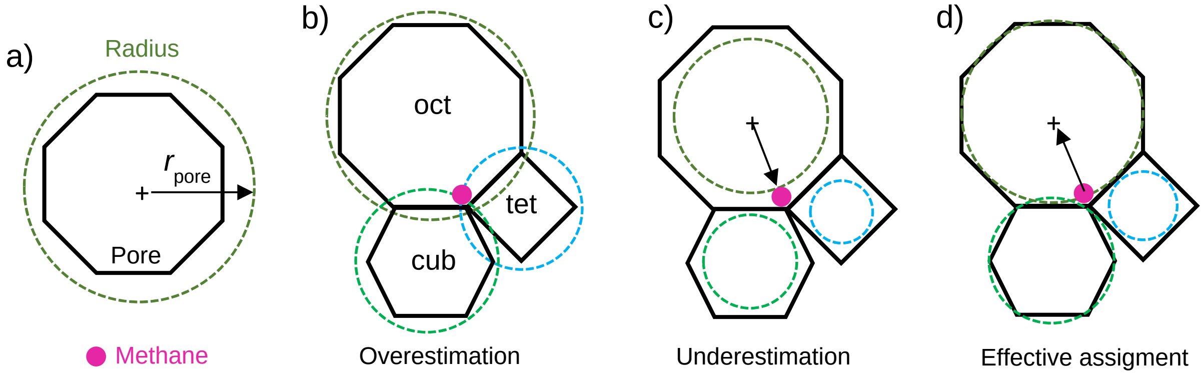

Supplementary Figure 82. Scheme of radial distribution analysis in a trimodal pore structure similar to DUT-49.

Supplementary References

1 Schindelin, J. *et al.* Fiji: an open-source platform for biological-image analysis. *Nat. Methods* **9**, 676-682, doi:10.1038/nmeth.2019 (2012).

2 Krause, S. *et al.* Adsorption Contraction Mechanics: Understanding Breathing Energetics in Isoreticular Metal–Organic Frameworks. *J. Phys. Chem. C* **122**, 19171-19179, doi:10.1021/acs.jpcc.8b04549 (2018).

3 Rowland, C. A. *et al.* Methane Storage in Paddlewheel-Based Porous Coordination Cages. *J. Am. Chem. Soc.* **140**, 11153-11157, doi:10.1021/jacs.8b05780 (2018).

4 Lu, W. *et al.* Highly porous metal-organic framework sustained with 12-connected nanoscopic octahedra. *Dalton Trans.* **42**, 1708-1714, doi:10.1039/C2DT32479B (2013).

5 Weseliński, Ł. J., Luebke, R. & Eddaoudi, M. A Convenient Preparation of 9H-Carbazole-3,6-dicarbonitrile and 9H-Carbazole-3,6-dicarboxylic Acid. *Synthesis* **46**, 596-599, doi:10.1055/s-0033-1340557 (2014).

6 Stoeck, U., Senkovska, I., Bon, V., Krause, S. & Kaskel, S. Assembly of metal-organic polyhedra into highly porous frameworks for ethene delivery. *Chem. Commun.* **51**, 1046-1049, doi:10.1039/C4CC07920E (2015).

7 Krause, S. *et al.* The effect of crystallite size on pressure amplification in switchable porous solids. *Nat. Commun.* **9**, 1573, doi:10.1038/s41467-018-03979-2 (2018).

8 Krause, S. *et al.* A pressure-amplifying framework material with negative gas adsorption transitions. *Nature* **532**, 348-352, doi:10.1038/nature17430 (2016).

9 Helmholtz-Zentrum Berlin für Materialien und Energie. The MX beamlines BL14.1-3 at BESSY II. *JLSRF* **2**, 1-6, doi:10.17815/jlsrf-2-64 (2016).

10 Battye, T. G. G., Kontogiannis, L., Johnson, O., Powell, H. R. & Leslie, A. G. W. iMOSFLM: a new graphical interface for diffraction-image processing with MOSFLM. *Acta Crystallogr. D* **67**, 271-281, doi:10.1107/S0907444910048675 (2011).

11 Winn, M. D. *et al.* Overview of the CCP4 suite and current developments. *Acta Crystallogr. D* **67**, 235-242, doi:10.1107/S0907444910045749 (2011).

12 Sparta, K. M., Krug, M., Heinemann, U., Mueller, U. & Weiss, M. S. XDSAPP2.0. *J. Appl. Crystallogr.* **49**, 1085-1092, doi:10.1107/S1600576716004416 (2016).

13 Sheldrick, G. A short history of SHELX. *Acta Crystallogr. A* **64**, 112-122, doi:10.1107/S0108767307043930 (2008).

14 Sheldrick, G. Crystal structure refinement with SHELXL. *Acta Crystallogr. C* **71**, 3-8, doi:10.1107/S2053229614024218 (2015).

15 Spek, A. Structure validation in chemical crystallography. *Acta Crystallogr. D* **65**, 148-155, doi:10.1107/S090744490804362X (2009).

16 Willems, T. F., Rycroft, C. H., Kazi, M., Meza, J. C. & Haranczyk, M. Algorithms and tools for high-throughput geometry-based analysis of crystalline porous materials. *Microporous Mesoporous Mater.* **149**, 134-141, doi:10.1016/j.micromeso.2011.08.020 (2012).

17 Gómez-Gualdrón, D. A., Moghadam, P. Z., Hupp, J. T., Farha, O. K. & Snurr, R. Q. Application of Consistency Criteria To Calculate BET Areas of Micro- And Mesoporous Metal–Organic Frameworks. *J. Am. Chem. Soc.* **138**, 215-224, doi:10.1021/jacs.5b10266 (2016).

18 Farha, O. K. *et al.* Metal–Organic Framework Materials with Ultrahigh Surface Areas: Is the Sky the Limit? *J. Am. Chem. Soc.* **134**, 15016-15021, doi:10.1021/ja3055639 (2012).

19 Llewellyn, P. L. & Maurin, G. Gas adsorption microcalorimetry and modelling to characterise zeolites and related materials. *C. R. Chim.* **8**, 283-302, doi:10.1016/j.crci.2004.11.004 (2004).

20 NIST REFPROP (National Institute of Standards and Technology, 2018).

21 Beurroies, I. *et al.* Using Pressure to Provoke the Structural Transition of Metal–Organic Frameworks. *Angew. Chem. Int. Ed.* **49**, 7526-7529, doi:10.1002/anie.201003048 (2010).

22 Ramaswamy, P. *et al.* Mechanical properties of a gallium fumarate metal-organic framework: a joint experimental-modelling exploration. *J. Mater. Chem. A* **5**, 11047-11054, doi:10.1039/C7TA01559C (2017).

23 Yot, P. G. *et al.* Metal-organic frameworks as potential shock absorbers: the case of the highly flexible MIL-53(Al). *Chem. Commun.* **50**, 9462-9464, doi:10.1039/C4CC03853C (2014).

24 Yot, P. G. *et al.* Exploration of the mechanical behavior of metal organic frameworks UiO-66(Zr) and MIL-125(Ti) and their NH2 functionalized versions. *Dalton Trans.* **45**, 4283-4288, doi:10.1039/c5dt03621f (2016).

25 Yot, P. G. *et al.* Impact of the Metal Centre and Functionalization on the Mechanical Behaviour of MIL-53 Metal–Organic Frameworks. *Eur. J. Inorg. Chem.* **2016**, 4424-4429, doi:10.1002/ejic.201600263 (2016).

26 Henke, S. *et al.* Pore closure in zeolitic imidazolate frameworks under mechanical pressure. *Chem. Sci.*, doi:10.1039/C7SC04952H (2018).

27 Helmholtz-Zentrum Berlin für Materialien und Energie. KMC-2: an X-ray beamline with dedicated diffraction and XAS endstations at BESSY II. *JLSRF* **2**, A49, doi:10.17815/jlsrf-2-65 (2016).

28 Bon, V. *et al.* In situ monitoring of structural changes during the adsorption on flexible porous coordination polymers by X-ray powder diffraction: Instrumentation and experimental results. *Microporous Mesoporous Mater.* **188**, 190-195, doi:10.1016/j.micromeso.2013.12.024 (2014).

29 Datasqueeze 2.2.9 Graphical Tool for X-ray Data Analysis v. 2.2.9 (2012).

30 Wojdyr, M. Fityk: a general-purpose peak fitting program. *J. Appl. Crystallogr.* **43**, 1126-1128, doi:10.1107/S0021889810030499 (2010).

31 Bail, A. L. Whole powder pattern decomposition methods and applications: A retrospection. *Powder Diffr.* **4**, 316-326, doi:10.1154/1.2135315 (2005).

32 FULLPROF (Laboratoire Le'on Brillouin (CEA–CNRS), France, 2006).

33 Material Studio 5.0 (Accelrys Software Inc., San Diego, USA, 2009).

34 Helmholtz-Zentrum Berlin für Materialien und Energie. E9: The Fine Resolution Powder Diffractometer (FIREPOD) at BER II. *JLSRF* **3**, A103, doi:10.17815/jlsrf-3-127 (2017).

35 Roisnel, T. & Rodríquez-Carvajal, J. WinPLOTR: A Windows Tool for Powder Diffraction Pattern Analysis. *Mater. Sci. Forum* **378-381**, 118-123, doi:10.4028/www.scientific.net/MSF.378-381.118 (2001).

36 Getzschmann, J. *et al.* Methane storage mechanism in the metal-organic framework Cu3(btc)2: An in situ neutron diffraction study. *Microporous Mesoporous Mater.* **136**, 50-58, doi:10.1016/j.micromeso.2010.07.020 (2010).

37 Wu, H. *et al.* Metal-Organic Frameworks with Exceptionally High Methane Uptake: Where and How is Methane Stored? *Chem. Eur. J.* **16**, 5205-5214, doi:10.1002/chem.200902719 (2010).

38 Wu, H., Zhou, W. & Yildirim, T. Methane Sorption in Nanoporous Metal−Organic Frameworks and First-Order Phase Transition of Confined Methane. *J. Phys. Chem. C* **113**, 3029-3035, doi:10.1021/jp8103276 (2009).

39 Bogdan, K., Ege, D., Filip, F., L., L. P. & Lucyna, F. Adsorption-Induced Structural Phase Transformation in Nanopores. *Angew. Chem. Int. Ed.* **56**, 16243-16246, doi:10.1002/anie.201708993 (2017).

40 Carrington, E. J., Vitorica-Yrezabal, I. J. & Brammer, L. Crystallographic studies of gas sorption in metal-organic frameworks. *Acta Crystallogr. B* **70**, 404-422, doi:10.1107/S2052520614009834 (2014).

41 Glaser, T. *et al.* Infrared study of the MoO3 doping efficiency in 4,4′-bis(N-carbazolyl)-1,1′-biphenyl (CBP). *Org. Electron.* **14**, 575-583, doi:10.1016/j.orgel.2012.11.031 (2013).

42 Stoeck, U., Krause, S., Bon, V., Senkovska, I. & Kaskel, S. A highly porous metal-organic framework, constructed from a cuboctahedral super-molecular building block, with exceptionally high methane uptake. *Chem. Commun.* **48**, 10841-10843, doi:10.1039/C2CC34840C (2012).

43 Bureekaew, S. *et al.* MOF-FF – A flexible first-principles derived force field for metal-organic frameworks. *Phys. Status Solidi B* **250**, 1128-1141, doi:10.1002/pssb.201248460 (2013).
