# Supplementary material for: Towards general network architecture design criteria for negative gas adsorption transitions in ultraporous frameworks
Source: Nat Commun. 2019 Aug 12;10:3632. doi: 10.1038/s41467-019-11565-3 (PMC6690989; doi:10.1038/s41467-019-11565-3)
Supplement: Supplementary file 2 — Description of Additional Supplementary Files [file 41467_2019_11565_MOESM2_ESM.pdf]

Description of Additional Supplementary Files

File Name: Supplementary Data 1
Description: Simulated structures of op and cp phases of DUT-48, -46, -49, -50, and -151.

File Name: Supplementary Data 2
Description: Rietveld-refined structures of CD4-filled DUT-48, -49, and -50.

File Name: Supplementary Movie 1
Description: Cartoon of the pore filling mechanism upon methane adsorption in DUT-49op at 111 K.
