# Supplementary figures and images for: Towards general network architecture design criteria for negative gas adsorption transitions in ultraporous frameworks
Source: Nat Commun. 2019 Aug 12;10:3632. doi: 10.1038/s41467-019-11565-3 (PMC6690989; doi:10.1038/s41467-019-11565-3)

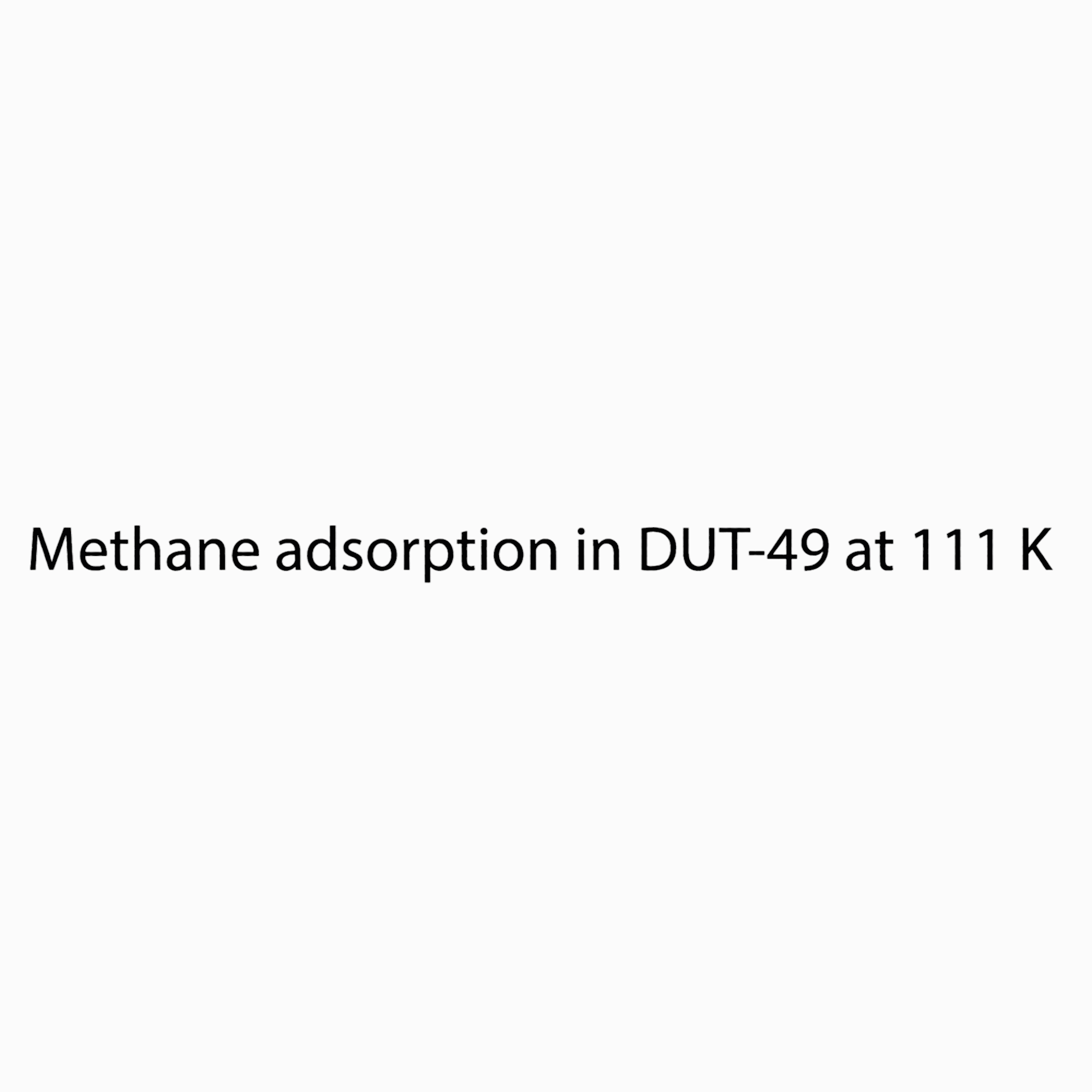

Supplement: Supplementary file 5 — Supplementary Movie 1 [file 41467_2019_11565_MOESM5_ESM.gif]
